# Chalcogen bonds: Hierarchical *ab initio* benchmark and density functional theory performance study

**DOI:** 10.1002/jcc.26489

**Published:** 2021-02-05

**Authors:** Lucas de Azevedo Santos, Teodorico C. Ramalho, Trevor A. Hamlin, F. Matthias Bickelhaupt

**Affiliations:** ^1^ Department of Theoretical Chemistry, Amsterdam Institute of Molecular and Life Sciences (AIMMS), Amsterdam Center for Multiscale Modeling (ACMM) Vrije Universiteit Amsterdam Amsterdam Netherlands; ^2^ Department of Chemistry, Institute of Natural Sciences Federal University of Lavras Lavras Brazil; ^3^ Center for Basic and Applied Research University Hradec Kralove Hradec Kralove Czech Republic; ^4^ Institute for Molecules and Materials Radboud University Nijmegen Nijmegen Netherlands

**Keywords:** benchmark study, chalcogen bonds, coupled‐cluster, density functional calculations, noncovalent interactions

## Abstract

We have performed a hierarchical *ab initio* benchmark and DFT performance study of D_2_Ch•••A^−^ chalcogen bonds (Ch = S, Se; D, A = F, Cl). The *ab initio* benchmark study is based on a series of ZORA‐relativistic quantum chemical methods [HF, MP2, CCSD, CCSD(T)], and all‐electron relativistically contracted variants of Karlsruhe basis sets (ZORA‐def2‐SVP, ZORA‐def2‐TZVPP, ZORA‐def2‐QZVPP) with and without diffuse functions. The highest‐level ZORA‐CCSD(T)/ma‐ZORA‐def2‐QZVPP counterpoise‐corrected complexation energies (Δ*E*_CPC_) are converged within 1.1–3.4 kcal mol^−1^ and 1.5–3.1 kcal mol^−1^ with respect to the method and basis set, respectively. Next, we used the ZORA*‐*CCSD(T)/ma‐ZORA‐def2‐QZVPP (Δ*E*_CPC_) as reference data for analyzing the performance of 13 different ZORA‐relativistic DFT approaches in combination with the Slater‐type QZ4P basis set. We find that the three‐best performing functionals are M06‐2X, B3LYP, and M06, with mean absolute errors (MAE) of 4.1, 4.2, and 4.3 kcal mol^−1^, respectively. The MAE for BLYP‐D3(BJ) and PBE amount to 8.5 and 9.3 kcal mol^−1^, respectively.

## Graphical abstract

**Fast and accurate!** We have, for the first time, computed chalcogen bonds complexation energies in a procedure involving both a hierarchical series of *ab initio* methods and a hierarchical series of Gaussian‐type basis sets. Our best reference data, ZORA‐CCSD(T)/BS3+, identify the M06, M06‐2X, and B3LYP functionals as accurate DFT approaches for investigating chalcogen bonds in more complex systems.

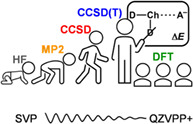

## 1 INTRODUCTION

**SCHEME 1:**
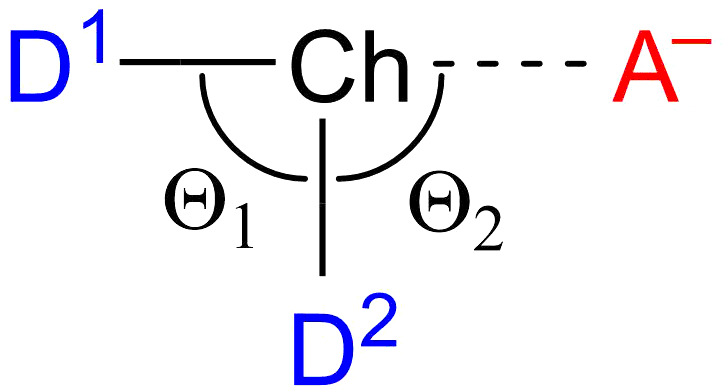
Chalcogen‐bonded D_2_Ch•••A^−^ model complexes (Ch = S, Se; D, A = F, Cl)

Chalcogen bonding has emerged as a key noncovalent interaction with several applications including supramolecular chemistry,^1^ biochemistry,^2^ and catalysis.^3^ The chalcogen‐bond (ChB) is defined as the net‐attractive noncovalent interaction, in a D_2_Ch•••A complex, between a chalcogen‐bond donor D_2_Ch, a Lewis‐acid, and a chalcogen‐bond acceptor A^−^ (or A), a Lewis‐base, in which Ch stands for a chalcogen atom, i.e., an atom of group 16 (Scheme 1).^4a^ The “σ‐hole interaction” between a positive region on the electrostatic potential surface on the chalcogen atom and a negatively charged density on the ChB acceptor is usually invoked to characterize the ChB.^4^ Despite this, recent studies have shown that the strength of the ChB is, instead, correlated to the electron‐accepting capacity of the σ*‐type LUMO of the chalcogen molecule.^5^ The debate over the origin and fundamental bonding mechanism of the ChB continues to stimulate much interest in the literature.

Density functional theory (DFT) based Kohn‐Sham molecular orbital analysis has been paramount for our understanding of bonding mechanisms and the nature of chemical phenomena.^6^ Selection of the appropriate density functional approximation to investigate chalcogen bonding is critical to ensure trust‐worthy results, but unfortunately this is not entirely straightforward, as the question of which approximate functional works best is highly dependent on the property and system of interest.

The first purpose of this work is to provide a detailed benchmark study of high‐level relativistic *ab initio* methods and focus on the investigation of ChB, using the D_2_Ch molecules as chalcogen‐bond donors and the halides A^−^ as chalcogen‐bond acceptors (see Scheme 1). Our model complexes systematically varies the substituent (D), the chalcogen atom (Ch), the acceptor (A^−^), and is the perfect archetype for strongly bound chalcogen systems studied experimentally.^2^, ^3^ This is done by computing the D_2_Ch•••A^−^ complexation energies Δ*E* for the first time in a procedure involving both a hierarchical series of *ab initio* methods [HF, MP2, CCSD, and CCSD(T)]^7^ in combination with a hierarchical series of Gaussian‐type basis sets of increasing flexibility, polarization (up to *g* functions), and diffuseness, thereby eclipsing the two other benchmarks based on a single‐shot CCSD(T) approach.^7i,j^ Interestingly, the predictions of Δ*E* by both benchmarks for the same systems can differ by up to 10 kcal mol^−1^. The basis set superposition error (BSSE) has been accounted for through the counterpoise correction (CPC) of Boys and Bernardi.^8^

The second purpose of this work is to evaluate the performance of 13 different density functionals in combination with ADF's Slater‐type QZ4P basis set (vide infra) for predicting the ChB energy Δ*E* against our best *ab initio* benchmark. Thus, we perform an extensive analysis to highlight the importance of diffuse and polarization functions in the basis set, the role of the BSSE, and the necessity of Coulomb correlation as well as the extent to which the approach has converged with respect to the level of correlation treatment and basis set quality. Our analyses identify the B3LYP and M06‐2X functionals, along with the M06 DFT approach as appropriate and computationally efficient alternatives to expensive high‐level *ab initio* computations of chalcogen‐bonded complexes.

## 2 THEORETICAL METHODS

### 2.1 *Ab initio* geometries and energies

All *ab initio* calculations were carried out using ORCA.^9^ The atomic orbitals were described by the all‐electron scalar relativistically contracted variants of Gaussian‐type def2‐XVP(P) (X = S, TZ, QZ) basis sets with polarization functions (up to *g* functions) in the series BS1 to BS3 (see Table 1).^10^ The series BS1+ to BS3+ result from BS1 to BS3 after adding extra *s* and *p* minimally augmented (ma) diffuse functions (see Table 1).^10c^ For each of the six basis sets (BS#), the equilibrium geometry was computed using coupled‐cluster singles and doubles with perturbative triples, i.e., at CCSD(T)/BS#.^11^ Then, for each BS# and corresponding CCSD(T)/BS# geometry, energies were evaluated along the following hierarchical series of quantum chemical methods: Hartree‐Fock theory (HF/BS#), second‐order Møller‐Plesset perturbation theory (MP2/BS#),^12^ coupled‐cluster with single and double excitations (CCSD/BS#)^13^ and CCSD(T)/BS#.^11^ The scalar relativistic effects were accounted for using the scalar zeroth‐order regular approximation (ZORA).^14^ Inclusion of relativistic effects are necessary for heavier chalcogen‐bonded systems and without ZORA, our counterpoise‐corrected complexation energies Δ*E*_CPC_ are significantly under‐bound. For example, for Cl_2_Se•••Cl^−^ the Δ*E*_CPC_ is −31.2 kcal mol^−1^ at CCSD(T)/BS3+ and −34.3 kcal mol^−1^ at ZORA‐CCSD(T)/BS3+. For the lighter chalcogen systems, such as F_2_S•••F^−^, this effect is smaller and Δ*E*_CPC_ is −45.1 kcal mol^−1^ at CCSD(T)/BS3+ and −45.2 kcal mol^−1^ at ZORA‐CCSD(T)/BS3+.

**TABLE 1:** Number of relativistically contracted basis functions for ZORA‐def2‐ basis sets without (BS) and with (BS+) diffuse functions for F, S, Cl, and Se elements.

Basis set	Label	F	S and Cl	Se
ZORA‐def2‐SVP	BS1	3s2p1d	6s3p1d	9s6p3d
ZORA‐def2‐TZVPP	BS2	6s3p2d1f	8s4p3d1f	10s8p4d1f
ZORA‐def2‐QZVPP	BS3	8s4p3d2f1g	11s7p4d2f1g	14s11p4d4f1g
ma‐ZORA‐def2‐SVP	BS1+	4s3p1d	7s4p1d	10s7p3d
ma‐ZORA‐def2‐TZVPP	BS2+	7s4p2d1f	9s5p3d1f	11s9p4d1f
ma‐ZORA‐def2‐QZVPP	BS3+	9s5p3d2f1g	12s8p4d2f1g	15s12p4d4f1g

### 2.2 DFT geometries and energies

All DFT calculations were carried out using the Amsterdam Density Functional (ADF) program.^15^ The equilibrium geometries and energies of chalcogen‐bonded complexes were computed at different DFT levels using (i) the GGA based functionals: PBE,^16^ BP86,^17^ and BLYP^17^, ^18^; (ii) the hybrid functionals: B3LYP^19^ and BHANDH (50% HF exchange, 50% LDA exchange, and 100% LYP correlation^18^); (iii) the meta‐GGA based functionals: SSB‐D^20^ and M06‐L^21^; (iv) the meta‐hybrid functionals: M06,^21^ M06‐2X,^21^ and M06‐HF.^21^ The long range dispersion corrections were included into the B3LYP, BLYP, and SSB‐D functionals with Grimme's empirical D3 correction using the Becke‐Johnson (BJ) damping function.^22^ Energies and geometries were computed for each of the various DFT approaches with the QZ4P basis set.^23^ This is a large, uncontracted and relativistically optimized, all‐electron (i.e., no frozen core approximation) basis set of Slater‐type orbitals (STOs), which is of quadruple‐*ζ* quality for all atoms and has been augmented with the following sets of polarization and diffuse functions: two 3*d* and two 4*f* on fluorine, three 3*d* and two 4*f* on sulfur and chlorine, two 4*d* and three 4*f* on selenium. The molecular density was fitted by the systematically improvable Zlm fitting scheme. Scalar relativistic effects were accounted for using the zeroth‐order regular approximation (ZORA).^14^

## 3 RESULTS AND DISCUSSION

### 3.1 *Ab initio* geometries

**FIGURE 1:**
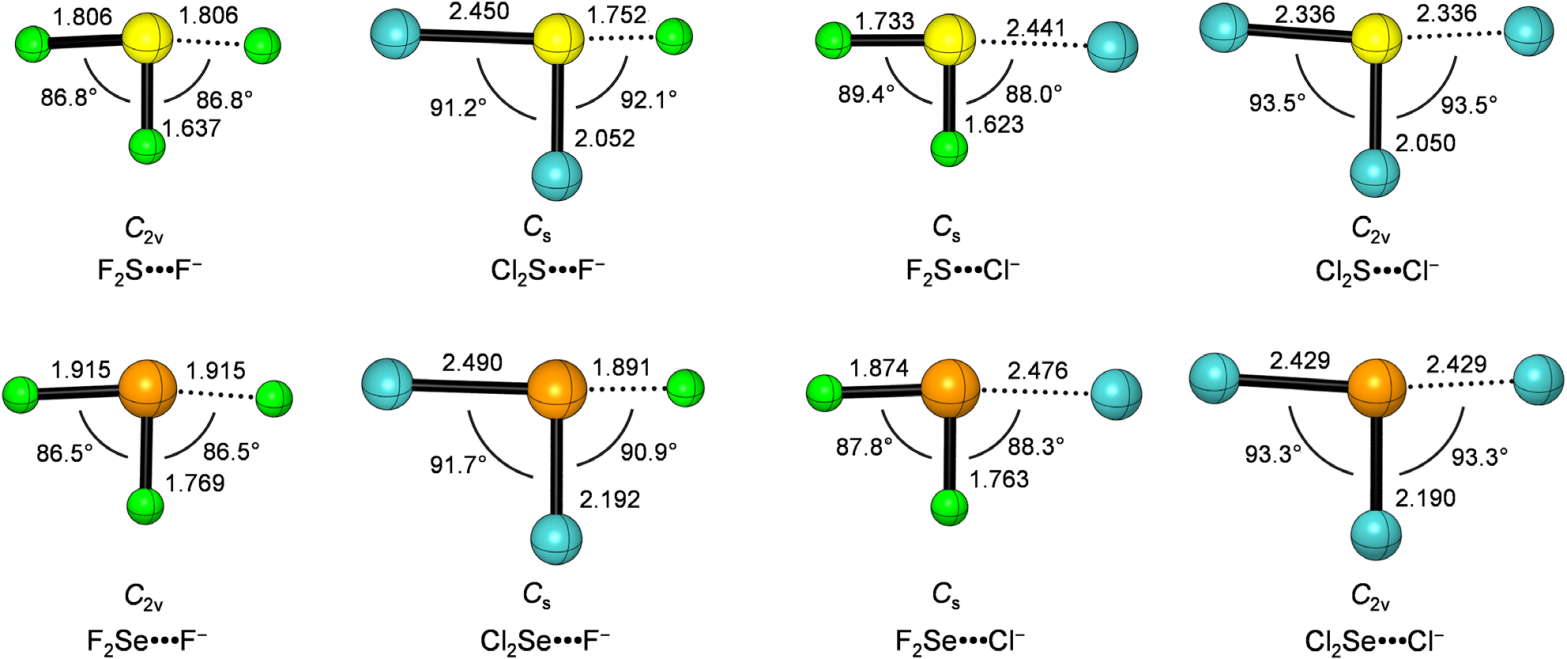
Geometries (in Å and degrees) and point group symmetries of D_2_Ch•••A^−^ complexes computed at ZORA‐CCSD(T)/BS3+.

First, we examine the equilibrium geometries of D_2_Ch•••A^−^ complexes (Ch = S, Se; D, A = F, Cl) which were fully optimized at the ZORA‐CCSD(T) level along with a hierarchic series of Gaussian‐type basis sets both with and without diffuse functions (see Table 1; for optimized Cartesian coordinates see Tables S10, S11 in the Supporting Information). The isolated halide and *C*_2v_ symmetric D_2_Ch neutral fragment form the stable T‐shaped, chalcogen‐bonded complexes D_2_Ch•••A^−^ which are of *C*_2v_ (D = A) or *C*_s_ symmetry (D ≠ A) (see Figure 1). All species have been verified through a vibrational analysis to represent equilibrium structures (no imaginary frequencies). Thus, we have a set of geometries that have been optimized at the same relativistic *ab initio* level along with each basis set considered in this work, without any structural or symmetry constraint (for complete structural details, see Tables S2 and S3 of the Supporting Information).

The chalcogen bond distance in the D_2_Ch•••A^−^ complexes become longer as the chalcogen atom (Ch) varies from S to Se and as the accepting halide (A^−^) varies from F^−^ to Cl^−^, and shorter as the substituent D varies from F to Cl (see Figure 1). Furthermore, the Θ_1_ and Θ_2_ angles (see Scheme 1) are slightly smaller than 90° for D = F and slightly larger than 90° for D = Cl. The key structural parameters (chalcogen bond distance and angles) converge faster as a function of basis‐set flexibility and polarization if diffuse functions are included in the basis set. For example, chalcogen bond lengths converge within 0.004–0.015 Å along the BS1 to BS3 series and within 0.000–0.010 Å along the BS1+ to BS3+ series (see Tables S2 and S3 in the Supporting Information). Interestingly, the differences in bond distances and angles of the D_2_Ch•••A^−^ complexes between using quadruple‐*ζ* basis sets basis sets with (BS3+) or without diffuse functions (BS3) are small, only ca. 0.001 Å and 0.1°. In the following, all ZORA‐CCSD(T) calculated geometries are used in the series of high‐level *ab initio* calculations that constitute our benchmark study of chalcogen bonds (ChB) complexation energies.

### 3.2 *Ab initio* Chalcogen bond energies

**FIGURE 2:**
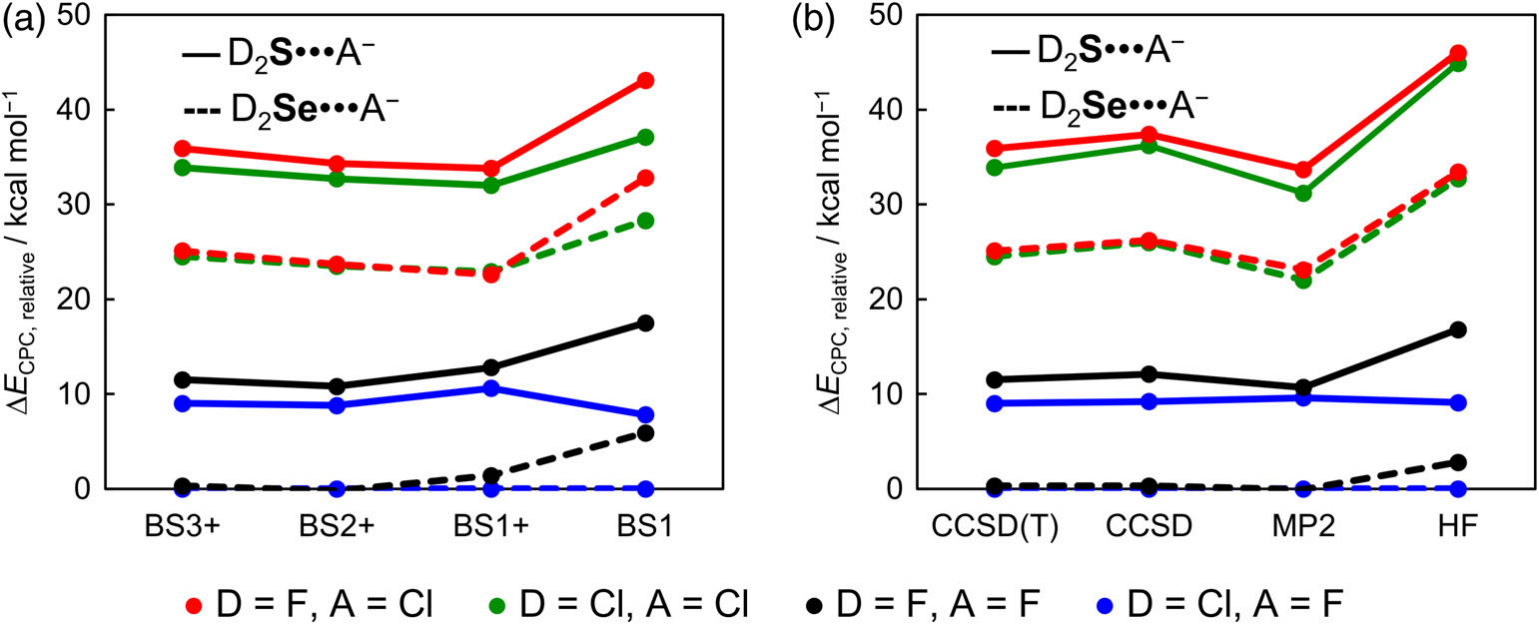
Trends in D_2_Ch•••A^−^ chalcogen‐bond strength relative to the most stable Cl_2_Se•••F^−^ complex along (a) ZORA‐CCSD(T)/BS# and (b) ZORA‐method/BS3+. Sulfur complexes in full lines and selenium complexes in dashed lines

**FIGURE 3:**
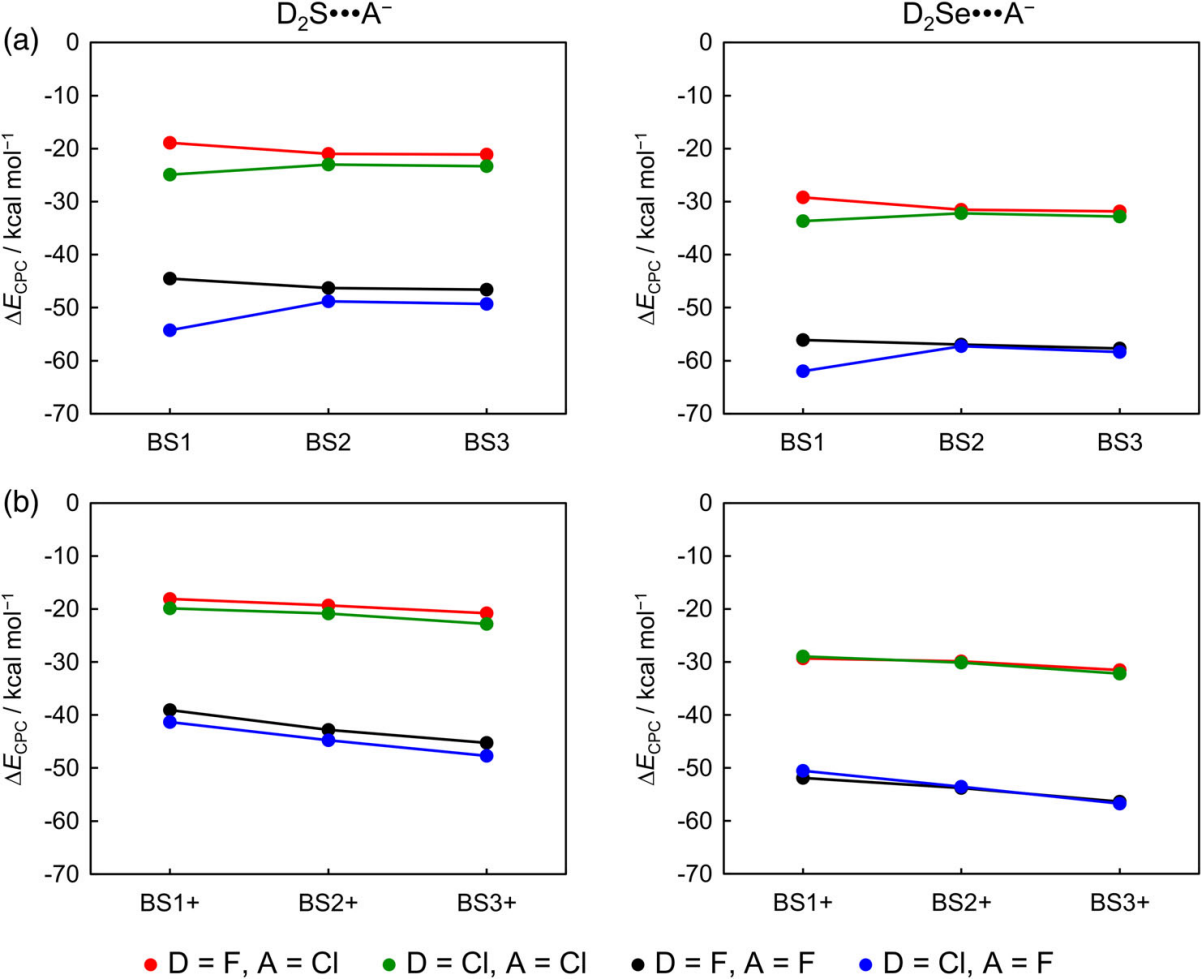
Counterpoise‐corrected ZORA‐CCSD(T) complexation energies (∆*E*_CPC_) for D_2_Ch•••A^−^ chalcogen‐bonded complexes along (a) BS1 to BS3 and (b) BS1+ to BS3+ basis sets

**FIGURE 4:**
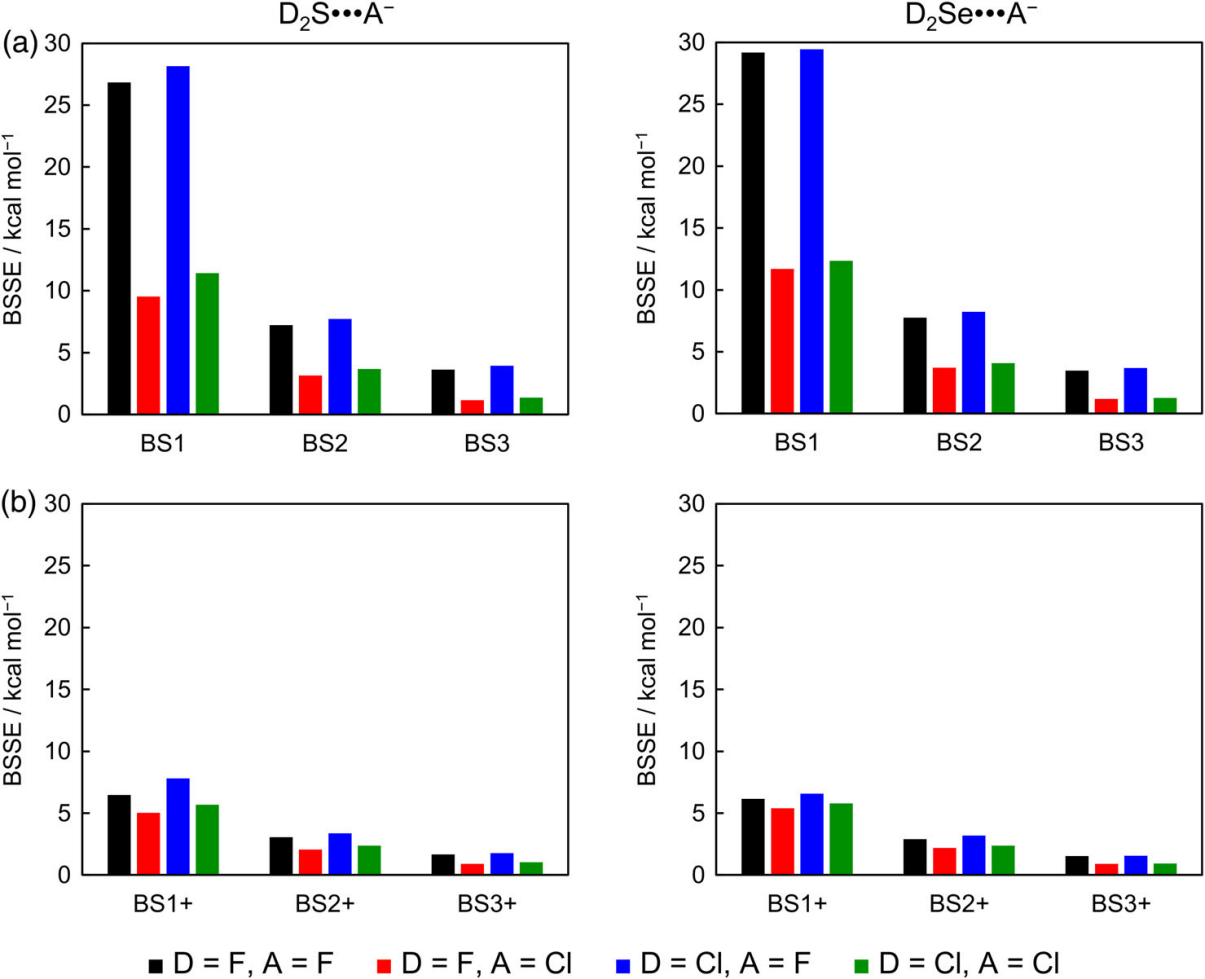
Basis set superposition error (BSSE) calculated at ZORA‐CCSD(T) level for D_2_Ch•••A^−^ chalcogen‐bonded complexes along (a) BS1 to BS3 and (b) BS1+ to BS3+ basis sets

**FIGURE 5:**
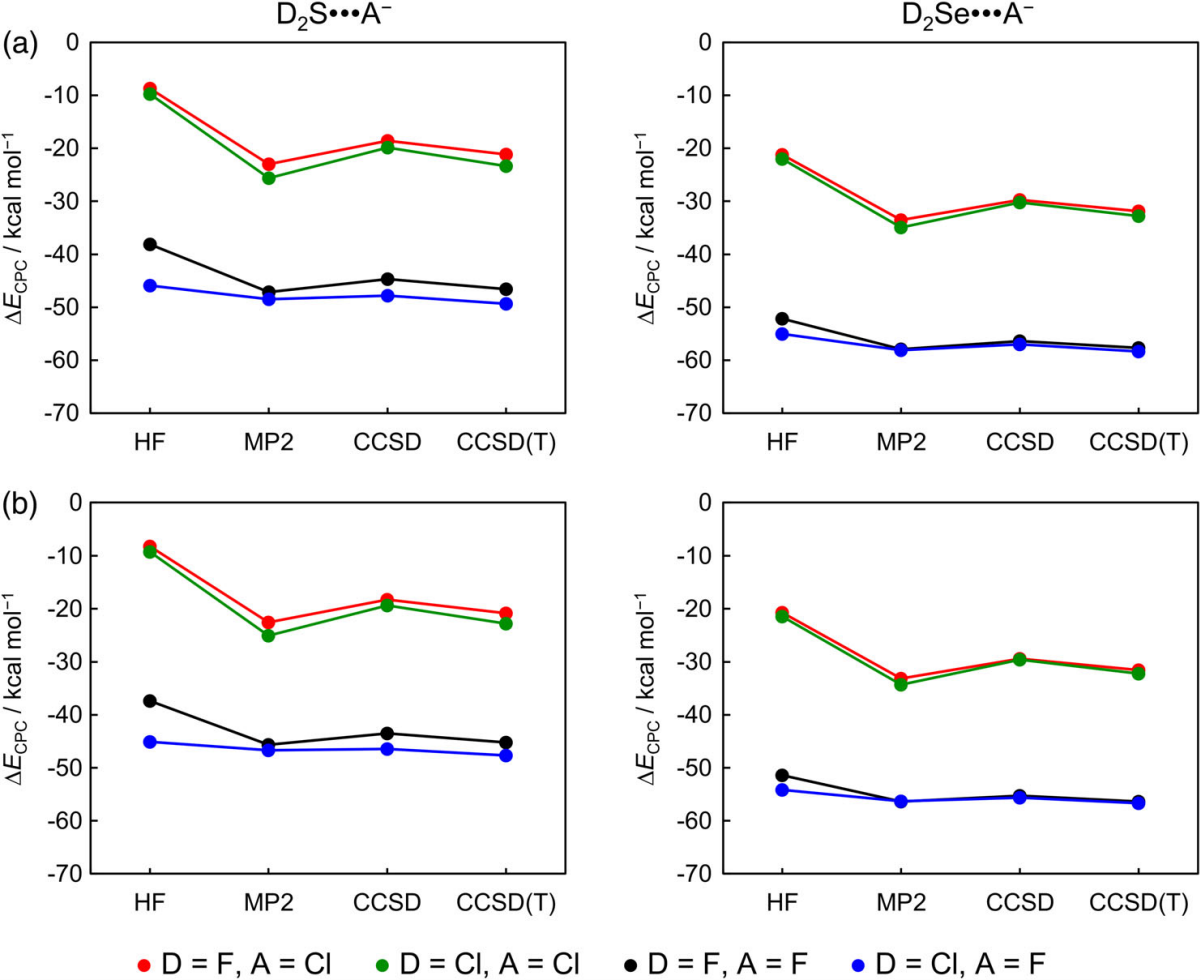
Counterpoise‐corrected ZORA‐CCSD(T) complexation energies (Δ*E*_CPC_) for D_2_Ch•••A^−^ chalcogen‐bonded complexes along the *ab initio* method in combination with (a) BS3 and (b) BS3+

Here, we report the first systematic investigation of the complexation energies, with (Δ*E*_CPC_) and without (Δ*E*) counterpoise corrections, as a function of a hierarchical series of *ab initio* methods and basis sets. The results of our *ab initio* computations are collected in Tables 2, 3, 4, 5 (Δ*E*_CPC_, Δ*E*, and BSSE; for thermodynamic values see Tables S8 and S9 in the Supporting Information) and graphically displayed in Figures 2, 3, 4, 5 (Δ*E*_CPC_ and BSSE). In general, we find that the same trends in chalcogen‐bond strengths emerge at all levels of theory, that is, chalcogen bonds become stronger as the chalcogen Ch varies from S to Se, the halide A^−^ varies from Cl^−^ to F^−^, and the substituents D from F to Cl (see Figure 2). Our best reference data, obtained using counterpoise*‐*corrected ZORA‐CCSD(T)/BS3+ energies, show that the D_2_Ch•••A^−^ chalcogen‐bond strength increases along F_2_S•••F^−^ to F_2_Se•••F^−^ from −45.2 to −56.4 kcal mol^−1^ and along F_2_Se•••Cl^−^ to F_2_Se•••F^−^ from −31.6 to −56.4 kcal mol^−1^. On the other hand, along F_2_S•••Cl^−^ to Cl_2_S•••Cl^−^, the chalcogen‐bond strength only marginally strengthens from −20.8 to −22.8 kcal mol^−1^. For smaller basis sets in combination with ZORA‐CCSD(T), this minor difference in stability along the variation on the substituent D becomes even smaller and, for BS1+ basis sets, the selenium bonds D_2_Se•••F^−^ become marginally stronger for D = F. Our best level ZORA‐CCSD(T)/BS3+ has converged within 1.5–3.1 kcal mol^−1^ in respect to the basis set series and, in combination with the BS3+ basis set, Δ*E*_CPC_ have converged within 1.1–3.4 kcal mol^−1^ along the series of *ab initio* methods.

**TABLE 2:** Complexation energies (in kcal mol^−1^) of D_2_S•••A^−^ chalcogen‐bonded complexes with (Δ*E*_CPC_) and without (Δ*E*) counterpoise corrections^a^

		F_2_S•••F^−^		F_2_S•••Cl^−^		Cl_2_S•••F^−^		Cl_2_S•••Cl^−^	
Method	Basis set	Δ*E*_CPC_	Δ*E*	Δ*E*_CPC_	Δ*E*	Δ*E*_CPC_	Δ*E*	Δ*E*_CPC_	Δ*E*
HF	BS1	−45.0	−63.3	−15.6	−21.9	−60.0	−78.7	−20.3	−27.5
	BS2	−39.7	−42.7	−10.6	−12.0	−47.1	−50.2	−11.7	−13.3
	BS3	−38.1	−39.3	−8.7	−9.0	−45.9	−47.1	−9.8	−10.1
MP2	BS1	−46.3	−72.8	−19.8	−28.9	−56.1	−84.0	−25.9	−36.9
	BS2	−47.6	−54.8	−23.0	−26.0	−49.0	−56.8	−25.4	−28.9
	BS3	−47.2	−50.8	−23.0	−24.2	−48.5	−52.5	−25.6	−27.1
CCSD	BS1	−44.0	−70.0	−18.2	−27.5	−54.0	−81.1	−23.2	−34.2
	BS2	−44.6	−51.0	−18.9	−21.7	−47.5	−54.4	−20.1	−23.4
	BS3	−44.7	−47.7	−18.6	−19.6	−47.8	−51.1	−19.9	−21.0
CCSD(T)	BS1	−44.5	−71.4	−18.9	−28.5	−54.2	−82.4	−24.9	−36.3
	BS2	−46.3	−53.5	−21.0	−24.2	−48.8	−56.5	−23.0	−26.7
	BS3	−46.6	−50.2	−21.1	−22.3	−49.3	−53.2	−23.3	−24.7
HF	BS1+	−37.0	−39.5	−11.2	−12.5	−46.9	−49.6	−12.9	−14.2
	BS2+	−37.3	−37.5	−8.5	−8.6	−44.7	−44.9	−9.4	−9.6
	BS3+	−37.4	−37.4	−8.2	−8.2	−45.1	−45.1	−9.3	−9.3
MP2	BS1+	−40.1	−46.0	−19.1	−23.8	−41.6	−48.9	−21.0	−26.3
	BS2+	−43.6	−46.4	−21.2	−23.0	−44.4	−47.4	−23.3	−25.4
	BS3+	−45.6	−47.2	−22.6	−23.5	−46.7	−48.4	−25.1	−26.1
CCSD	BS1+	−38.0	−44.0	−16.6	−21.3	−40.8	−48.0	−17.5	−22.8
	BS2+	−41.4	−44.0	−17.1	−18.8	−43.9	−46.7	−17.9	−19.8
	BS3+	−43.5	−44.8	−18.2	−19.0	−46.4	−47.8	−19.4	−20.2
CCSD(T)	BS1+	−39.1	−45.6	−18.1	−23.1	−41.3	−49.1	−19.9	−25.5
	BS2+	−42.8	−45.8	−19.3	−21.4	−44.8	−48.1	−20.9	−23.2
	BS3+	−45.2	−46.9	−20.8	−21.7	−47.7	−49.5	−22.8	−23.8

*Note*: ^a^Computed at ZORA‐Method/BS#//ZORA‐CCSD(T)/BS#.

**TABLE 3:** Complexation energies (in kcal mol^−1^) of D_2_Se•••A^*−*^ chalcogen‐bonded complexes with (Δ*E*_CPC_) and without (Δ*E*) counterpoise corrections.^a^

		F_2_Se•••F^−^		F_2_Se•••Cl^−^		Cl_2_Se•••F^−^		Cl_2_Se•••Cl^−^	
Method	Basis set	Δ*E*_CPC_	Δ*E*	Δ*E*_CPC_	Δ*E*	Δ*E*_CPC_	Δ*E*	Δ*E*_CPC_	Δ*E*
HF	BS1	−58.5	−78.5	−26.5	−34.1	−66.9	−86.7	−31.0	−38.8
	BS2	−53.4	−56.8	−23.1	−24.8	−56.0	−59.6	−23.7	−25.6
	BS3	−52.1	−53.3	−21.2	−21.6	−55.0	−56.3	−22.0	−22.4
MP2	BS1	−57.6	−86.6	−30.2	−41.4	−64.1	−93.3	−35.1	−47.0
	BS2	−57.8	−65.7	−33.3	−36.9	−58.0	−66.3	−34.5	−38.4
	BS3	−57.9	−61.4	−33.6	−34.8	−58.1	−61.9	−34.9	−36.3
CCSD	BS1	−55.9	−84.1	−28.7	−40.0	−61.7	−90.1	−32.6	−44.5
	BS2	−55.9	−62.8	−29.8	−33.2	−56.2	−63.5	−30.0	−33.7
	BS3	−56.4	−59.3	−29.8	−30.8	−57.0	−60.1	−30.2	−31.3
CCSD(T)	BS1	−56.1	−85.3	−29.2	−40.9	−62.0	−91.4	−33.7	−46.0
	BS2	−56.9	−64.7	−31.5	−35.3	−57.3	−65.5	−32.2	−36.3
	BS3	−57.7	−61.2	−31.9	−33.0	−58.4	−62.1	−32.8	−34.1
HF	BS1+	−51.8	−54.1	−23.0	−24.2	−54.8	−57.0	−24.7	−25.9
	BS2+	−51.1	−51.3	−21.0	−21.1	−53.6	−53.8	−21.5	−21.6
	BS3+	−51.4	−51.4	−20.8	−20.8	−54.2	−54.2	−21.5	−21.5
MP2	BS1+	−52.7	−58.4	−30.6	−35.6	−51.4	−57.5	−30.6	−36.0
	BS2+	−54.2	−56.8	−31.7	−33.6	−53.8	−56.6	−32.4	−34.6
	BS3+	−56.4	−57.8	−33.2	−34.1	−56.3	−57.8	−34.3	−35.3
CCSD	BS1+	−51.2	−56.8	−28.0	−33.0	−50.0	−56.0	−27.5	−32.9
	BS2+	−52.9	−55.4	−28.1	−29.9	−52.8	−55.5	−27.9	−29.9
	BS3+	−55.3	−56.5	−29.4	−30.1	−55.6	−56.8	−29.6	−30.4
CCSD(T)	BS1+	−51.9	−58.0	−29.3	−34.7	−50.5	−57.1	−29.0	−34.8
	BS2+	−53.8	−56.7	−29.9	−32.1	−53.6	−56.7	−30.1	−32.5
	BS3+	−56.4	−57.9	−31.6	−32.4	−56.7	−58.3	−32.2	−33.2

*Note*: ^a^Computed at ZORA‐Method/BS#//ZORA‐CCSD(T)/BS#.

**TABLE 4:** Basis set superposition error (BSSE, in kcal mol^−1^) of D_2_S•••A^*−*^ chalcogen‐bonded complexes.^a^

Method	Basis set	F_2_S•••F^−^	F_2_S•••Cl^−^	Cl_2_S•••F^−^	Cl_2_S•••Cl^−^
HF	BS1	18.3	6.3	18.7	7.3
	BS2	3.0	1.4	3.1	1.5
	BS3	1.1	0.3	1.2	0.4
MP2	BS1	26.6	9.1	27.9	10.9
	BS2	7.3	3.0	7.8	3.5
	BS3	3.7	1.2	4.0	1.4
CCSD	BS1	25.9	9.2	27.1	11.0
	BS2	6.4	2.8	6.9	3.3
	BS3	3.0	1.0	3.3	1.1
CCSD(T)	BS1	26.8	9.5	28.1	11.4
	BS2	7.2	3.2	7.7	3.7
	BS3	3.6	1.2	3.9	1.4
HF	BS1+	2.5	1.3	2.7	1.3
	BS2+	0.2	0.1	0.2	0.1
	BS3+	0.0	0.0	0.0	0.0
MP2	BS1+	5.9	4.7	7.3	5.3
	BS2+	2.7	1.9	3.0	2.1
	BS3+	1.6	0.9	1.7	1.1
CCSD	BS1+	6.0	4.7	7.2	5.3
	BS2+	2.6	1.7	2.8	2.0
	BS3+	1.3	0.7	1.4	0.8
CCSD(T)	BS1+	6.5	5.0	7.8	5.7
	BS2+	3.0	2.1	3.4	2.4
	BS3+	1.7	0.9	1.8	1.0

*Note*: ^a^Computed at ZORA‐Method/BS#//ZORA‐CCSD(T)/BS#.

**TABLE 5:** Basis set superposition error (BSSE, in kcal mol^−1^) of D_2_Se•••A^*−*^ chalcogen‐bonded complexes.^a^

Method	Basis set	F_2_Se•••F^−^	F_2_Se•••Cl^−^	Cl_2_Se•••F^−^	Cl_2_Se•••Cl^−^
HF	BS1	20.0	7.6	19.8	7.8
	BS2	3.4	1.7	3.6	1.9
	BS3	1.1	0.4	1.2	0.4
MP2	BS1	29.0	11.2	29.2	11.8
	BS2	7.8	3.5	8.3	3.9
	BS3	3.5	1.2	3.8	1.3
CCSD	BS1	28.2	11.3	28.4	11.9
	BS2	6.9	3.3	7.3	3.7
	BS3	2.9	1.0	3.1	1.1
CCSD(T)	BS1	29.2	11.7	29.5	12.3
	BS2	7.8	3.7	8.2	4.1
	BS3	3.5	1.2	3.7	1.3
HF	BS1+	2.3	1.2	2.2	1.2
	BS2+	0.2	0.1	0.3	0.2
	BS3+	0.0	0.0	0.0	0.0
MP2	BS1+	5.7	5.0	6.1	5.4
	BS2+	2.6	2.0	2.9	2.2
	BS3+	1.4	0.9	1.5	1.0
CCSD	BS1+	5.7	5.1	6.0	5.4
	BS2+	2.4	1.8	2.7	2.0
	BS3+	1.2	0.7	1.2	0.7
CCSD(T)	BS1+	6.1	5.4	6.6	5.8
	BS2+	2.9	2.2	3.2	2.4
	BS3+	1.5	0.9	1.6	0.9

*Note*: ^a^Computed at ZORA‐Method/BS#//ZORA‐CCSD(T)/BS#.

Despite the trend in D_2_Ch•••A^−^ chalcogen‐bond strength being qualitatively the same at all levels of *ab initio* theory in our double hierarchical series (in QM method and in basis set), major variations of up to ca. 20 kcal mol^−1^ in absolute values are observed between the various levels (see Tables 2 and 3). For example, with Cl_2_S•••F^−^ the Δ*E*_CPC_ varies from −60.0 to −49.6 kcal mol^−1^ at both ZORA‐HF/BS1 and ZORA‐CCSD(T)/BS3+ levels, respectively. The high accuracy of our best level ZORA‐CCSD(T)/BS3+ can be attributed to four main factors: i) inclusion of additional *s* and *p* diffuse functions to accurately describe anions, as one would expect; ii) use of a highly flexible basis set with diffuse functions to minimize BSSE; iii) introduction of Coulomb correlation; and iv) inclusion of polarization functions especially for highly correlated methods.

We first examine Δ*E*_CPC_ as a function of the basis set. In general, a strengthening of the D_2_Ch•••A^−^ chalcogen bond occurs as the flexibility of the basis set is increased, and Δ*E*_CPC_ is only converged at larger basis sets (see Figure 3). An exception to this trend is observed for ChB Δ*E*_CPC_ values computed with the small basis set BS1, which lacks diffuse functions. For example, the Δ*E*_CPC_ for Cl_2_Se•••F^−^ that is already −62.0 kcal mol^−1^ at ZORA‐CCSD(T)/BS1 slightly weakens to −58.4 kcal mol^−1^ at ZORA‐CCSD(T)/BS3 (see Figure 3(A)), whereas the Δ*E*_CPC_ is −50.5 kcal mol^−1^ at ZORA‐CCSD(T)/BS1+ and strengthens to −56.7 kcal mol^−1^ at ZORA‐CCSD(T)/BS3+ (see Figure 3(B)). This is caused by the breathing orbitals of the anionic halide fragments going from diffuse in the isolated anion to more compact upon forming the ChB complex, which leads to charge delocalization over the molecular system.^24^, ^25^ In the absence of diffuse functions, the complexation energy is overestimated due to the artificially high energy of the anion because the charge density cannot breath, i.e., expand, in order to relieve electron–electron repulsion in the negatively charged species. This explains the possibly misleading conclusion that the Δ*E*_CPC_ converges faster along the BS1 to BS3 series compared to the BS1+ to BS3+ series and, therefore, the use of the basis set series without diffuse functions would be more appropriate. Later on, we illustrate that this is only a consequence of these complexation energies being ‘corrected’ by the BSSE.

The BSSE becomes significantly smaller with the addition of diffuse functions and decreases from 1.2–3.9 kcal mol^−1^ at ZORA‐CCSD(T)/BS3 to 0.9–1.8 kcal mol^−1^ at ZORA‐CCSD(T)/BS3+ (see Tables 4, 5, 6). However, the BSSE is large, in particular, for highly correlated methods and smaller basis sets without diffuse functions, that is, at the ZORA‐CCSD(T)/BS1 level (see Figure 4). As a result, the ZORA‐CCSD(T) Δ*E*_CPC_ are better for the BS1+ to BS3+ series but become similar to the series without diffuse functions as the BSSE simultaneously decreases as the basis sets size increases. Both basis sets series, indeed, converge to a similar value independently of the number of diffuse functions, but this result is fortuitous due to the BSSE correction that damps any fluctuations along the BS1 to BS3 series. In fact, the uncorrected ZORA‐CCSD(T) complexation energies Δ*E* converges significantly faster along the BS1+ to BS3+ series (within 0.3–1.5 kcal mol^−1^) compared to the BS1 to BS3 series (within 1.9–3.5 kcal mol^−1^) (see Tables 2 and 3). This is, again, due to the poor description of the anionic reactants by basis sets without diffuse functions. This effect is particularly apparent at HF where Coulomb correlation is absent, mainly for systems involving the compact atom F^−^.^24a^ For example, the Δ*E* for Cl_2_Se•••F^−^ that is −86.7 kcal mol^−1^ at ZORA‐HF/BS1 significantly weakens to −57.0 kcal mol^−1^ at ZORA‐HF/BS1+, whereas, for Cl_2_Se•••Cl^−^, the Δ*E* is −38.8 kcal mol^−1^ at ZORA‐HF/BS1 and weakens to −25.9 kcal mol^−1^ at ZORA‐HF/BS1+ (see Table 3).

**TABLE 6:** ZORA‐DFT/BS complexation energies (in kcal mol^−1^) of representative D_2_Ch•••A^−^ chalcogen‐bonded complexes.^a^

DFT/BS	F_2_S•••Cl^−^	Cl_2_Se•••F^−^
B3LYP/TZ2P	−26.0	−65.3
M06/TZ2P	−25.6	−66.1
M06‐2X/TZ2P	−25.5	−64.0
B3LYP/QZ4P	−23.5	−62.3
M06/QZ4P	−23.1	−63.6
M06‐2X/QZ4P	−23.7	−61.9
Benchmark^a^	−20.8	−56.7

*Note*: ^a^Δ*E*_CPC_ computed at ZORA‐CCSD(T)/BS3+.

Lastly, inclusion of Coulomb correlation is critical to achieve accurate chalcogen‐bond energies. At HF, the D_2_Ch•••A^−^ complexes are weakly bound and enter into stronger chalcogen bonds as Coulomb correlation is introduced (see Figure 5). For example, from HF to CCSD(T), the Δ*E*_CPC_ for F_2_S•••F^−^ strengthens from −38.1 to −46.6 kcal mol^−1^ for BS3 and from −37.4 to −45.2 kcal mol^−1^ for BS3+ (see Table 2). We also note that the stabilization of Δ*E*_CPC_ due to the increasing of basis set size is more pronounced for high correlated methods. For example, from BS1+ to BS3+, the Δ*E*_CPC_ for F_2_Se•••F^−^ slightly varies from −51.8 to −51.4 kcal mol^−1^ at HF level and strengthens from −51.9 to −56.4 kcal mol^−1^ at CCSD(T) level (see Tables 2 and 3). This is due to the well‐known fact that correlated *ab initio* methods strongly depend on the extent of polarization functions to generate configurations through which the wavefunction can describe the correlation hole.^7c^ On the other hand, at the HF level without Coulomb correlation, there is much less sensitivity of Δ*E*_CPC_ towards increasing the flexibility and polarization functions of the basis set. Taken altogether, our benchmark approach, based on hierarchical series, reveals that our best estimates are converged with regards to correlation and basis set within 1.1–3.4 kcal mol^−1^ and 1.5–3.1 kcal mol^−1^, respectively, and provides the most accurate benchmark to date, surpassing the recently published benchmark based on a single‐shot CCSD(T) approach.^7i^ In the next section, we discuss the ability of DFT to describe Coulomb correlation compared to our ZORA‐CCSD(T)/BS3+ benchmark.

### 3.3 Performance of density functional approximations

**FIGURE 6:**
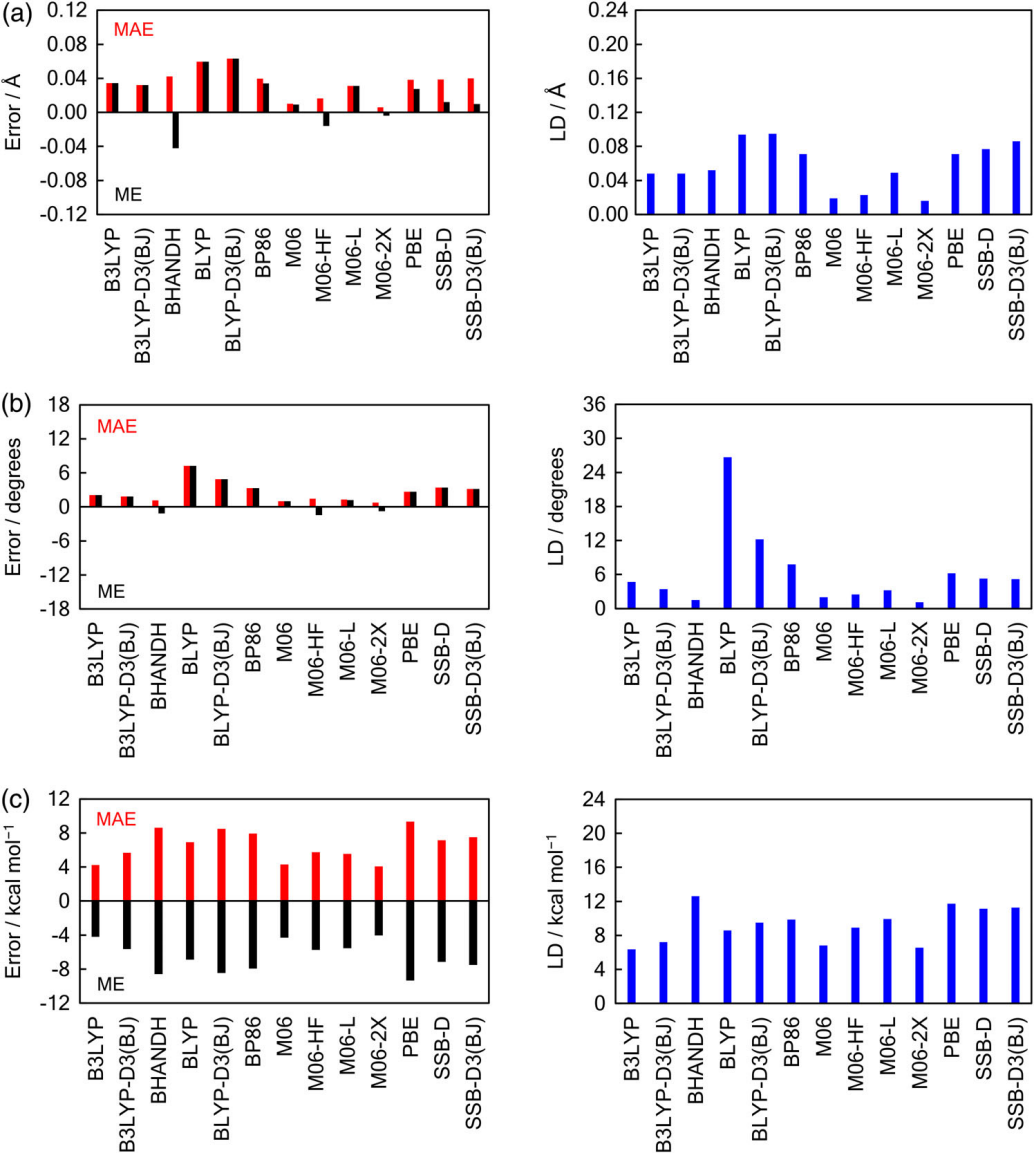
Mean absolute error (MAE, red), mean error (ME, black), and largest deviation (LD, blue) of the ZORA‐DFT/QZ4P functionals relative to the ZORA‐CCSD(T)/BS3+ (a) Ch•••A^−^ bond lengths, (b) bond angles Θ_2_, and (c) D_2_Ch•••A^−^ counterpoise‐corrected complexation energies

Finally, we have computed the complexation energies Δ*E* for various GGAs, meta‐GGAs, hybrid, and meta‐hybrid functionals in combination with the all‐electron QZ4P basis set and ZORA for relativistic effects on optimized geometries at the same level. The performance of the density functionals is discussed by comparing the resulting Δ*E* with our best *ab initio* ZORA‐CCSD(T)/BS3+ level. These results are graphically illustrated by the bar diagrams in Figure 6 (mean absolute error, mean error, and largest deviation) and collected in Tables S4 and S5 (complexation energies, mean absolute error, mean error, and largest deviation, see Supporting Information).

The Δ*E* computed at the DFT levels follow the same trends as those at ZORA‐CCSD(T)/BS3+, that is, chalcogen bonds D_2_Ch•••A^−^ become stronger as the chalcogen Ch varies from S to Se, the halide A^−^ varies from Cl^−^ to F^−^ and the substituents D from F to Cl. SSB‐D and SSB‐D3(BJ) are exceptions, whereby the ChB becomes more stabilizing when D varies from Cl to F (see Table S4 in the Supporting Information). The main trends in bond lengths and angles are also in line with the *ab initio* methods where the D_2_Ch•••A^−^ chalcogen bond becomes longer as Ch varies from S to Se and as A^−^ varies from F^−^ to Cl^−^ and shorter as D varies from F to Cl (see Tables S6 and S7; for optimized Cartesian coordinates see Tables S12‐S27 in the Supporting Information). In general, we find that the density functionals give longer chalcogen bonds and bigger bond angles Θ_2_ (Scheme 1) compared to our best level ZORA‐CCSD(T)/BS3+ geometries (see Figure 6). The best overall agreement with our best *ab initio* level geometries is with the meta‐hybrid M06, M06‐HF, M06‐2X functionals (MAE of 0.006–0.017 Å for bond lengths and MAE of 0.7–1.5 degrees for bond angles). The GGAs BLYP and BLYP‐D3(BJ) perform the worst and have the largest MAEs up to 0.063 Å and 7.2 degrees.

The mean absolute error (MAE), mean error (ME), and largest deviation (LD) for the 13 density functionals are computed relative to ZORA‐CCSD(T)/BS3+. Three main observations emerge: (i) M06‐2X, B3LYP, and M06 perform the best; (ii) BHANDH, BLYP‐D3(BJ), and BP86 perform the worst; and (iii) all 13 density functionals overestimate the Δ*E* compared to ZORA‐CCSD(T)/BS3+. The best overall agreement with the *ab initio* benchmark is with the meta‐hybrid functionals, M06‐2X and M06 (MAE of 4.1–4.3 kcal mol^−1^ and LD of 6.6–6.8 kcal mol^−1^) and by the popular B3LYP hybrid functional (MAE 4.2 kcal mol^−1^ and LD of 6.4 kcal mol^−1^) (see Figure 6(c)). GGAs perform the worst and have the largest MAEs up to 9.3 kcal mol^−1^. BLYP is the best GGA with a MAE of 6.9 kcal mol^−1^ and LD of 8.6 kcal mol^−1^. Addition of an explicit dispersion correction (D3) and damping function (BJ) for the BLYP and B3LYP functionals results in less accurate Δ*E* values and increases the MAE to 8.5 and 5.7 kcal mol^−1^, respectively.

The ME is negative, and its absolute value is equal to the MAE for all density functionals, that is, the stabilization of the D_2_Ch•••A^−^ chalcogen‐bonded complexes is overestimated by all functionals in this study. Nevertheless, our best performing density functionals together with the Slater‐type QZ4P basis set have the same trends in chemical stability and geometry as our ZORA‐CCSD(T)/BS3+ benchmark, with relatively small deviations from the *ab initio* Δ*E*_CPC_. For larger chalcogen‐bonded systems, the smaller Slater‐type TZ2P basis set may be used, which also provides satisfactory results in comparison with our best *ab initio* level. For our three‐best density functionals, B3LYP, M06‐2X, and M06, the Δ*E* is ca. 2 kcal mol^−1^ more over‐binding for TZ2P than for QZ4P (see Table 6), that is, the overestimation on the stability of chalcogen‐bonded systems increases. This results in larger errors relative to our best estimate and the B3LYP, M06‐2X, and M06 density functionals in combination with TZ2P basis set turn out to have similar accuracy as the ZORA‐BLYP/QZ4P. Thus, we identify not only B3LYP and M06‐2X,^7i^ but also M06, in combination with the all‐electron QZ4P basis set, to be reasonable approaches for computing the complexation energies of chalcogen bonds without relying on expensive *ab initio* methods.

## 4 CONCLUSIONS

We have computed a ZORA‐CCSD(T)/BS3+ benchmark for the archetypal chalcogen‐bonded model complexes D_2_Ch•••A^−^ (Ch = S, Se; D, A = F, Cl) that derives from a hierarchical series of relativistic *ab initio* methods and basis sets. The counterpoise‐corrected ZORA‐CCSD(T)/ma‐ZORA‐def2‐QZVPP level is converged within 1.5–3.1 kcal mol^−1^ and 1.1–3.4 kcal mol^−1^ with respect to the basis set size and *ab initio* method, respectively. Our benchmark data show that chalcogen bonds (ChB) in D_2_Ch•••A^−^ become stronger for the heavier chalcogen Ch, the lighter halide A^−^, and for the less electronegative halogen substituent D.

Basis sets including diffuse functions are required for the calculation of accurate complexation energies for the chalcogen‐bonded complexes D_2_Ch•••A^−^ involving anions. Addition of diffuse functions yields smaller BSSE and faster convergence with respect to the basis set size and *ab initio* method. However, as the BSSE simultaneously decreases as the flexibility of the basis set size increases, the uncorrected and counterpoise‐corrected complexation energies become similar for larger basis sets, with or without diffuse functions. Coulomb correlation is also crucial, and, for highly correlated methods, addition of polarization functions is necessary to accurately describe the correlation hole.

The performance of 13 relativistic (ZORA) density functionals for describing the complexation energies of ChB was evaluated. Best agreement with our hierarchical *ab initio* benchmark is achieved by hybrid and meta‐hybrid DFT functions, which overestimate the bond strength with mean absolute errors up to 4.3 kcal mol^−1^. Neither GGA nor meta‐GGA DFT approaches can achieve this accuracy. The BLYP functional, which is the best performing GGA approach, overestimates complexation energies by 6.9 kcal mol^−1^. Taken altogether, M06‐2X and M06 and B3LYP in combination with the all‐electron QZ4P basis are accurate, efficient, and non‐expensive methods for the routine investigation of chalcogen bonds.

## Supporting information


**Table S1** Number of relativistically contracted basis functions for ZORA‐def2‐ basis sets without (BS) and with (BS+) diffuse functions for F, S, Cl and Se elements.
**Table S2.** Ab initio bond lengths and angles (in Å and degrees) of D_2_S∙∙∙A^−^ chalcogen‐bonded complexes.
**Table S3.** Ab initio bond lengths and angles (in Å and degrees) of D_2_Se∙∙∙A^−^ chalcogen‐bonded complexes.
**Table S4.** Complexation energies (in kcal mol^−1^) of D_2_Ch∙∙∙A^−^ chalcogen‐bonded complexes.
**Table S5.** The mean error (ME), mean absolute error (MAE), and largest deviation (LD) of ZORA‐DFT/QZ4P approaches relative to the geometries (in Å and degrees) and counterpoise corrected complexation energies (in kcal mol^−1^) of D_2_Ch∙∙∙A^−^ complexes computed at ZORA‐CCSD(T)/BS3 + .
**Table S6.** Representative DFT bond lengths and angles (in Å and degrees) of D_2_S∙∙∙A^−^ chalcogen‐bonded complexes.
**Table S7.** Representative DFT bond lengths and angles (in Å and degrees) of D_2_Se∙∙∙A^−^ chalcogen‐bonded complexes.
**Table S8.** Thermodynamic values (in kcal mol^−1^ at 298 K) associated with formation of D_2_S∙∙∙A^−^ chalcogen‐bonded complexes for representative methods.
**Table S9.** Thermodynamic values (in kcal mol^−1^ at 298 K) associated with formation of D_2_Se∙∙∙A^−^ chalcogen‐bonded complexes for representative methods.
**Table S10.** Cartesian coordinates, electronic energies, *H*, *TS*, and *G* (in a.u. at 298 K) for all stationary points computed at ZORA‐CCSD(T) with ZORA‐def2 basis sets in the gas phase using ORCA.
**Table S11.** Cartesian coordinates, electronic energies, *H*, *TS*, and *G* (in a.u. at 298 K) for all stationary points computed at ZORA‐CCSD(T) with ma‐ZORA‐def2 basis sets in the gas phase using ORCA.
**Table S12.** Cartesian coordinates, bonding energies, *H*, *TS*, and *G* (in a.u. at 298 K) for all stationary points computed at ZORA‐B3LYP/QZ4P in the gas phase using ADF.
**Table S13.** Cartesian coordinates, bonding energies, *H*, *TS*, and *G* (in a.u. at 298 K) for all stationary points computed at ZORA‐B3LYP‐D3(BJ)/QZ4P in the gas phase using ADF.
**Table S14.** Cartesian coordinates, bonding energies, *H*, *TS*, and *G* (in a.u. at 298 K) for all stationary points computed at ZORA‐BHANDH/QZ4P in the gas phase using ADF.
**Table S15.** Cartesian coordinates, bonding energies, *H*, *TS*, and *G* (in a.u. at 298 K) for all stationary points computed at ZORA‐BLYP/QZ4P in the gas phase using ADF.
**Table S16.** Cartesian coordinates, bonding energies, *H*, *TS*, and *G* (in a.u. at 298 K) for all stationary points computed at ZORA‐BLYP‐D3(BJ)/QZ4P in the gas phase using ADF.
**Table S17.** Cartesian coordinates, bonding energies, *H*, *TS*, and *G* (in a.u. at 298 K) for all stationary points computed at ZORA‐BP86/QZ4P in the gas phase using ADF.
**Table S18.** Cartesian coordinates, bonding energies, *H*, *TS*, and *G* (in a.u. at 298 K) for all stationary points computed at ZORA‐M06/QZ4P in the gas phase using ADF.
**Table S19.** Cartesian coordinates, bonding energies, *H*, *TS*, and *G* (in a.u. at 298 K) for all stationary points computed at ZORA‐M06‐HF/QZ4P in the gas phase using ADF.
**Table S20.** Cartesian coordinates, bonding energies, *H*, *TS*, and *G* (in a.u. at 298 K) for all stationary points computed at ZORA‐M06‐L/QZ4P in the gas phase using ADF.
**Table S21.** Cartesian coordinates, bonding energies, *H*, *TS*, and *G* (in a.u. at 298 K) for all stationary points computed at ZORA‐M06‐2X/QZ4P in the gas phase using ADF.
**Table S22** Cartesian coordinates, bonding energies, *H*, *TS*, and *G* (in a.u. at 298 K) for all stationary points computed at ZORA‐PBE/QZ4P in the gas phase using ADF.
**Table S23.** Cartesian coordinates, bonding energies, *H*, *TS*, and *G* (in a.u. at 298 K) for all stationary points computed at ZORA‐SSB‐D/QZ4P in the gas phase using ADF.
**Table S24.** Cartesian coordinates, bonding energies, *H*, *TS*, and *G* (in a.u. at 298 K) for all stationary points computed at ZORA‐SSB‐D3(BJ)/QZ4P in the gas phase using ADF.
**Table S25.** Cartesian coordinates, bonding energies, *H*, *TS*, and *G* (in a.u. at 298 K) for all stationary points computed at ZORA‐B3LYP/TZ2P in the gas phase using ADF.
**Table S26.** Cartesian coordinates, bonding energies, *H*, *TS*, and *G* (in a.u. at 298 K) for all stationary points computed at ZORA‐M06/TZ2P in the gas phase using ADF.
**Table S27.** Cartesian coordinates, bonding energies, *H*, *TS*, and *G* (in a.u. at 298 K) for all stationary points computed at ZORA‐M06‐2X/TZ2P in the gas phase using ADF.
