# Supplementary material for: Chalcogen bonds: Hierarchical ab initio benchmark and density functional theory performance study
Source: J Comput Chem. 2021 Feb 5;42(10):688–98. doi: 10.1002/jcc.26489 (PMC7986859; doi:10.1002/jcc.26489)
Supplement: Supplementary file 1 — Table S1 Number of relativistically contracted basis functions for ZORA‐def2‐ basis sets without (BS) and with (BS+) diffuse functions for F, S, Cl and Se elements. Table S2. Ab initio bond lengths and angles (in Å and degrees) of D2S∙∙∙A− chalcogen‐bonded complexes. Table S3. Ab initio bond lengths and angles (in Å and degrees) of D2Se∙∙∙A− chalcogen‐bonded complexes. Table S4. Complexation energies (in kcal mol−1) of D2Ch∙∙∙A− chalcogen‐bonded complexes. Table S5. The mean error (ME), mean absolute error (MAE), and largest deviation (LD) of ZORA‐DFT/QZ4P approaches relative to the geometries (in Å and degrees) and counterpoise corrected complexation energies (in kcal mol−1) of D2Ch∙∙∙A− complexes computed at ZORA‐CCSD(T)/BS3 + . Table S6. Representative DFT bond lengths and angles (in Å and degrees) of D2S∙∙∙A− chalcogen‐bonded complexes. Table S7. Representative DFT bond lengths and angles (in Å and degrees) of D2Se∙∙∙A− chalcogen‐bonded complexes. Table S8. Thermodynamic values (in kcal mol−1 at 298 K) associated with formation of D2S∙∙∙A− chalcogen‐bonded complexes for representative methods. Table S9. Thermodynamic values (in kcal mol−1 at 298 K) associated with formation of D2Se∙∙∙A− chalcogen‐bonded complexes for representative methods. Table S10. Cartesian coordinates, electronic energies, H, TS, and G (in a.u. at 298 K) for all stationary points computed at ZORA‐CCSD(T) with ZORA‐def2 basis sets in the gas phase using ORCA. Table S11. Cartesian coordinates, electronic energies, H, TS, and G (in a.u. at 298 K) for all stationary points computed at ZORA‐CCSD(T) with ma‐ZORA‐def2 basis sets in the gas phase using ORCA. Table S12. Cartesian coordinates, bonding energies, H, TS, and G (in a.u. at 298 K) for all stationary points computed at ZORA‐B3LYP/QZ4P in the gas phase using ADF. Table S13. Cartesian coordinates, bonding energies, H, TS, and G (in a.u. at 298 K) for all stationary points computed at ZORA‐B3LYP‐D3(BJ)/QZ4P in the gas phase using ADF. Table S [file JCC-42-688-s001.docx]

# Supporting Information for

# **Chalcogen Bonds: Hierarchical *Ab Initio* Benchmark and DFT Performance Study**

Lucas de Azevedo Santos,^1,2^ Teodorico C. Ramalho,^2,3^ Trevor A. Hamlin,^1^ F. Matthias Bickelhaupt^1,4^

^1^Department of Theoretical Chemistry, Amsterdam Institute of Molecular and Life Sciences (AIMMS), Amsterdam Center for Multiscale Modeling (ACMM), Vrije Universiteit Amsterdam, Amsterdam, The Netherlands.

^2^Department of Chemistry, Institute of Natural Sciences, Federal University of Lavras, Lavras, Brazil.

^3^Center for Basic and Applied Research, University Hradec Kralove, Hradec Kralove, Czech Republic.

^4^Institute for Molecules and Materials, Radboud University Nijmegen, Nijmegen, The Netherlands.

# **Contents**

**Table S1.** Number of relativistically contracted basis functions for ZORA-def2- basis sets without (BS) and with (BS+) diffuse functions for F, S, Cl and Se elements.

**Table S2.** *Ab initio* bond lengths and angles (in Å and degrees) of D_2_S∙∙∙A^–^ chalcogen-bonded complexes.

**Table S3.** *Ab initio* bond lengths and angles (in Å and degrees) of D_2_Se∙∙∙A^–^ chalcogen-bonded complexes.

**Table S4.** Complexation energies (in kcal mol^-1^) of D_2_Ch∙∙∙A^–^ chalcogen-bonded complexes*.*

**Table S5.** The mean error (ME), mean absolute error (MAE), and largest deviation (LD) of ZORA-DFT/QZ4P approaches relative to the geometries (in Å and degrees) and counterpoise corrected complexation energies (in kcal mol^–1^) of D_2_Ch∙∙∙A^–^ complexes computed at ZORA-CCSD(T)/BS3+.

**Table S6.** Representative DFT bond lengths and angles (in Å and degrees) of D_2_S∙∙∙A^–^ chalcogen-bonded complexes.

**Table S7.** Representative DFT bond lengths and angles (in Å and degrees) of D_2_Se∙∙∙A^–^ chalcogen-bonded complexes.

**Table S8.** Thermodynamic values (in kcal mol^–1^ at 298 K) associated with formation of D_2_S∙∙∙A^–^ chalcogen-bonded complexes for representative methods.

**Table S9.** Thermodynamic values (in kcal mol^–1^ at 298 K) associated with formation of D_2_Se∙∙∙A^–^ chalcogen-bonded complexes for representative methods.

**Table S10.** Cartesian coordinates, electronic energies, *H, TS,* and *G* (in a.u. at 298 K) for all stationary points computed at ZORA-CCSD(T) with ZORA-def2 basis sets in the gas phase using ORCA.

**Table S11.** Cartesian coordinates, electronic energies, *H, TS,* and *G* (in a.u. at 298 K) for all stationary points computed at ZORA-CCSD(T) with ma-ZORA-def2 basis sets in the gas phase using ORCA.

**Table S12.** Cartesian coordinates, bonding energies, *H, TS,* and *G* (in a.u. at 298 K) for all stationary points computed at ZORA-B3LYP/QZ4P in the gas phase using ADF.

**Table S13.** Cartesian coordinates, bonding energies, *H, TS,* and *G* (in a.u. at 298 K) for all stationary points computed at ZORA-B3LYP-D3(BJ)/QZ4P in the gas phase using ADF.

**Table S14.** Cartesian coordinates, bonding energies, *H, TS,* and *G* (in a.u. at 298 K) for all stationary points computed at ZORA-BHANDH/QZ4P in the gas phase using ADF.

**Table S15.** Cartesian coordinates, bonding energies, *H, TS,* and *G* (in a.u. at 298 K) for all stationary points computed at ZORA-BLYP/QZ4P in the gas phase using ADF.

**Table S16.** Cartesian coordinates, bonding energies, *H, TS,* and *G* (in a.u. at 298 K) for all stationary points computed at ZORA-BLYP-D3(BJ)/QZ4P in the gas phase using ADF.

**Table S17.** Cartesian coordinates, bonding energies, *H, TS,* and *G* (in a.u. at 298 K) for all stationary points computed at ZORA-BP86/QZ4P in the gas phase using ADF.

**Table S18.** Cartesian coordinates, bonding energies, *H, TS,* and *G* (in a.u. at 298 K) for all stationary points computed at ZORA-M06/QZ4P in the gas phase using ADF.

**Table S19.** Cartesian coordinates, bonding energies, *H, TS,* and *G* (in a.u. at 298 K) for all stationary points computed at ZORA-M06-HF/QZ4P in the gas phase using ADF.

**Table S20.** Cartesian coordinates, bonding energies, *H, TS,* and *G* (in a.u. at 298 K) for all stationary points computed at ZORA-M06-L/QZ4P in the gas phase using ADF.

**Table S21.** Cartesian coordinates, bonding energies, *H, TS,* and *G* (in a.u. at 298 K) for all stationary points computed at ZORA-M06-2X/QZ4P in the gas phase using ADF.

**Table S22** Cartesian coordinates, bonding energies, *H, TS,* and *G* (in a.u. at 298 K) for all stationary points computed at ZORA-PBE/QZ4P in the gas phase using ADF.

**Table S23.** Cartesian coordinates, bonding energies, *H, TS,* and *G* (in a.u. at 298 K) for all stationary points computed at ZORA-SSB-D/QZ4P in the gas phase using ADF.

**Table S24.** Cartesian coordinates, bonding energies, *H, TS,* and *G* (in a.u. at 298 K) for all stationary points computed at ZORA-SSB-D3(BJ)/QZ4P in the gas phase using ADF.

**Table S25.** Cartesian coordinates, bonding energies, *H, TS,* and *G* (in a.u. at 298 K) for all stationary points computed at ZORA-B3LYP/TZ2P in the gas phase using ADF.

**Table S26.** Cartesian coordinates, bonding energies, *H, TS,* and *G* (in a.u. at 298 K) for all stationary points computed at ZORA-M06/TZ2P in the gas phase using ADF.

**Table S27.** Cartesian coordinates, bonding energies, *H, TS,* and *G* (in a.u. at 298 K) for all stationary points computed at ZORA-M06-2X/TZ2P in the gas phase using ADF.

**Table S1.** Number of relativistically contracted basis functions for ZORA-def2- basis sets without (BS) and with (BS+) diffuse functions for F, S, Cl and Se elements.

| **Basis set** | **Label** |  |  | **F** | **S and Cl** | **Se** |
| --- | --- | --- | --- | --- | --- | --- |
| ZORA-def2-SVP | BS1 |  |  | 3s2p1d | 6s3p1d | 9s6p3d |
| ZORA-def2-TZVPP | BS2 |  |  | 6s3p2d1f | 8s4p3d1f | 10s8p4d1f |
| ZORA-def2-QZVPP | BS3 |  |  | 8s4p3d2f1g | 11s7p4d2f1g | 14s11p4d4f1g |
|  |  |  |  |  |  |  |
| ma-ZORA-def2-SVP | BS1+ |  |  | 4s3p1d | 7s4p1d | 10s7p3d |
| ma-ZORA-def2-TZVPP | BS2+ |  |  | 7s4p2d1f | 9s5p3d1f | 11s9p4d1f |
| ma-ZORA-def2-QZVPP | BS3+ |  |  | 9s5p3d2f1g | 12s8p4d2f1g | 15s12p4d4f1g |

**Table S2.** *Ab initio* bond lengths and angles (in Å and degrees) of D_2_S∙∙∙A^–^ chalcogen-bonded complexes.^[a]^

|  | **F_2_S∙∙∙F^–^** | | | | |  | **F_2_S∙∙∙Cl^–^** | | | | |
| --- | --- | --- | --- | --- | --- | --- | --- | --- | --- | --- | --- |
| **Basis set** | ***r*_Ch–A_^–^** | ***r*_Ch–D_^1^** | ***r*_Ch–D_^2^** | **Θ_1_** | **Θ_2_** |  | ***r*_Ch–A_^–^** | ***r*_Ch–D_^1^** | ***r*_Ch–D_^2^** | **Θ_1_** | **Θ_2_** |
| **BS1** | 1.792 | 1.792 | 1.660 | 86.3 | 86.3 |  | 2.533 | 1.719 | 1.637 | 90.1 | 87.7 |
| **BS2** | 1.809 | 1.809 | 1.644 | 86.9 | 86.9 |  | 2.452 | 1.739 | 1.629 | 89.4 | 88.4 |
| **BS3** | 1.805 | 1.805 | 1.637 | 86.8 | 86.8 |  | 2.446 | 1.732 | 1.623 | 89.4 | 88.1 |
| **BS1+** | 1.856 | 1.856 | 1.679 | 87.4 | 87.4 |  | 2.511 | 1.783 | 1.666 | 89.7 | 88.7 |
| **BS2+** | 1.809 | 1.809 | 1.640 | 86.9 | 86.9 |  | 2.450 | 1.736 | 1.626 | 89.4 | 88.1 |
| **BS3+** | 1.806 | 1.806 | 1.637 | 86.8 | 86.8 |  | 2.441 | 1.733 | 1.623 | 89.4 | 88.0 |
|  | **Cl_2_S∙∙∙F^–^** | | | | |  | **Cl_2_S∙∙∙Cl^–^** | | | | |
|  | ***r*_Ch–A_^–^** | ***r*_Ch–D_^1^** | ***r*_Ch–D_^2^** | **Θ_1_** | **Θ_2_** |  | ***r*_Ch–A_^–^** | ***r*_Ch–D_^1^** | ***r*_Ch–D_^2^** | **Θ_1_** | **Θ_2_** |
| **BS1** | 1.734 | 2.526 | 2.108 | 90.7 | 92.2 |  | 2.393 | 2.393 | 2.100 | 93.5 | 93.5 |
| **BS2** | 1.759 | 2.457 | 2.068 | 91.7 | 92.1 |  | 2.349 | 2.349 | 2.064 | 94.0 | 94.0 |
| **BS3** | 1.752 | 2.451 | 2.053 | 91.2 | 92.1 |  | 2.337 | 2.337 | 2.050 | 93.5 | 93.5 |
| **BS1+** | 1.804 | 2.526 | 2.106 | 92.8 | 92.8 |  | 2.416 | 2.416 | 2.107 | 95.2 | 95.2 |
| **BS2+** | 1.755 | 2.460 | 2.062 | 91.6 | 92.2 |  | 2.346 | 2.346 | 2.060 | 93.9 | 93.9 |
| **BS3+** | 1.752 | 2.450 | 2.052 | 91.2 | 92.1 |  | 2.336 | 2.336 | 2.050 | 93.5 | 93.5 |

[a] Computed at ZORA-CCSD(T)/Basis set.

**Table S3.** *Ab initio* bond lengths and angles (in Å and degrees) of D_2_Se∙∙∙A^–^ chalcogen-bonded complexes.^[a]^

|  | **F_2_Se∙∙∙F^–^** | | | | |  | **F_2_Se∙∙∙Cl^–^** | | | | |
| --- | --- | --- | --- | --- | --- | --- | --- | --- | --- | --- | --- |
| **Basis set** | ***r*_Ch–A_^–^** | ***r*_Ch–D_^1^** | ***r*_Ch–D_^2^** | **Θ_1_** | **Θ_2_** |  | ***r*_Ch–A_^–^** | ***r*_Ch–D_^1^** | ***r*_Ch–D_^2^** | **Θ_1_** | **Θ_2_** |
| **BS1** | 1.902 | 1.902 | 1.784 | 86.4 | 86.4 |  | 2.521 | 1.859 | 1.773 | 88.5 | 88.5 |
| **BS2** | 1.919 | 1.919 | 1.775 | 86.5 | 86.5 |  | 2.489 | 1.877 | 1.767 | 88.0 | 88.8 |
| **BS3** | 1.915 | 1.915 | 1.770 | 86.5 | 86.5 |  | 2.479 | 1.873 | 1.763 | 87.9 | 88.5 |
| **BS1+** | 1.956 | 1.956 | 1.810 | 87.5 | 87.5 |  | 2.507 | 1.922 | 1.805 | 88.5 | 89.2 |
| **BS2+** | 1.917 | 1.917 | 1.770 | 86.4 | 86.4 |  | 2.480 | 1.876 | 1.763 | 87.7 | 88.4 |
| **BS3+** | 1.915 | 1.915 | 1.769 | 86.5 | 86.5 |  | 2.476 | 1.874 | 1.763 | 87.8 | 88.3 |
|  | **Cl_2_Se∙∙∙F^–^** | | | | |  | **Cl_2_Se∙∙∙Cl^–^** | | | | |
|  | ***r*_Ch–A_^–^** | ***r*_Ch–D_^1^** | ***r*_Ch–D_^2^** | **Θ_1_** | **Θ_2_** |  | ***r*_Ch–A_^–^** | ***r*_Ch–D_^1^** | ***r*_Ch–D_^2^** | **Θ_1_** | **Θ_2_** |
| **BS1** | 1.871 | 2.525 | 2.246 | 91.8 | 91.5 |  | 2.461 | 2.461 | 2.238 | 93.8 | 93.8 |
| **BS2** | 1.893 | 2.501 | 2.205 | 92.4 | 91.2 |  | 2.439 | 2.439 | 2.203 | 94.1 | 94.1 |
| **BS3** | 1.889 | 2.492 | 2.193 | 91.8 | 91.0 |  | 2.430 | 2.430 | 2.190 | 93.3 | 93.3 |
| **BS1+** | 1.939 | 2.524 | 2.243 | 93.1 | 91.5 |  | 2.476 | 2.476 | 2.243 | 94.6 | 94.6 |
| **BS2+** | 1.891 | 2.496 | 2.200 | 92.0 | 90.9 |  | 2.435 | 2.435 | 2.197 | 93.5 | 93.5 |
| **BS3+** | 1.891 | 2.490 | 2.192 | 91.7 | 90.9 |  | 2.429 | 2.429 | 2.190 | 93.3 | 93.3 |

[a] Computed at ZORA-CCSD(T)/Basis set.

**Table S4.** Complexation energies (in kcal mol^–1^) of D_2_Ch∙∙∙A^–^ chalcogen-bonded complexes.^[a]^

| **A**^–^ | **B3LYP** | **B3LYP^[b]^** | **BHANDH** | **BLYP** | **BLYP^[b]^** | **BP86** | **M06** | **M06-HF** | **M06-L** | **M06-2X** | **PBE** | **SSB-D** | **SSB^[b]^** |
| --- | --- | --- | --- | --- | --- | --- | --- | --- | --- | --- | --- | --- | --- |
|  | F_2_S∙∙∙A^–^ | | | | | | | | | | | | |
| **F**^–^ | –51.2 | –51.9 | –57.4 | –53.8 | –54.6 | –55.1 | –50.1 | –53.8 | –52.6 | –51.5 | –57.0 | –56.4 | –56.5 |
| **Cl**^–^ | –23.5 | –25.3 | –26.0 | –26.7 | –28.9 | –27.9 | –23.1 | –26.8 | –23.6 | –23.7 | –29.6 | –27.6 | –28.2 |
|  | Cl_2_S∙∙∙A^–^ | | | | | | | | | | | | |
| **F**^–^ | –54.1 | –54.9 | –60.3 | –56.3 | –57.2 | –57.4 | –53.1 | –56.6 | –55.4 | –54.3 | –58.5 | –55.4 | –55.5 |
| **Cl**^–^ | –25.8 | –28.2 | –27.5 | –29.6 | –32.3 | –30.4 | –25.3 | –28.3 | –24.9 | –25.0 | –31.4 | –26.8 | –27.5 |
|  | F_2_Se∙∙∙A^–^ | | | | | | | | | | | | |
| **F**^–^ | –61.7 | –62.4 | –68.2 | –63.4 | –64.3 | –64.6 | –62.4 | –62.3 | –63.9 | –61.7 | –66.5 | –66.3 | –66.4 |
| **Cl**^–^ | –33.8 | –35.7 | –37.6 | –36.1 | –38.3 | –37.5 | –34.6 | –36.4 | –34.5 | –33.8 | –39.1 | –37.3 | –37.7 |
|  | Cl_2_Se∙∙∙A^–^ | | | | | | | | | | | | |
| **F**^–^ | –62.3 | –63.1 | –67.9 | –64.4 | –65.2 | –65.3 | –63.6 | –60.0 | –66.6 | –61.9 | –66.5 | –64.8 | –64.9 |
| **Cl**^–^ | –34.8 | –37.1 | –37.3 | –38.4 | –40.4 | –38.7 | –35.7 | –35.3 | –36.3 | –34.0 | –39.7 | –36.1 | –36.7 |

[a] Computed at ZORA-DFT/QZ4P*.* [b] Includes the D3(BJ) dispersion correction.

**Table S5.** The mean error (ME), mean absolute error (MAE), and largest deviation (LD) of ZORA-DFT/QZ4P approaches relative to the geometries (in Å and degrees) and counterpoise corrected complexation energies (in kcal mol^–1^) of D_2_Ch∙∙∙A^–^ complexes computed at ZORA-CCSD(T)/BS3+.

|  |  | ***r*_Ch–A_^–^** | | |  | **Θ_2_** | | |  | **Δ*E*** | | |
| --- | --- | --- | --- | --- | --- | --- | --- | --- | --- | --- | --- | --- |
| **DFT** |  | **ME** | **MAE** | **LD** |  | **ME** | **MAE** | **LD** |  | **ME** | **MAE** | **LD** |
| **B3LYP** |  | 0.034 | 0.034 | 0.048 |  | 2.1 | 2.1 | 4.7 |  | –4.2 | 4.2 | 6.4 |
| **B3LYP-D3(BJ)** |  | 0.032 | 0.032 | 0.048 |  | 1.8 | 1.8 | 3.4 |  | –5.7 | 5.7 | 7.2 |
| **BHANDH** |  | –0.042 | 0.042 | 0.052 |  | –1.2 | 1.2 | 1.5 |  | –8.6 | 8.6 | 12.6 |
| **BLYP** |  | 0.060 | 0.060 | 0.094 |  | 7.2 | 7.2 | 26.7 |  | –6.9 | 6.9 | 8.6 |
| **BLYP-D3(BJ)** |  | 0.063 | 0.063 | 0.095 |  | 4.9 | 4.9 | 12.2 |  | –8.5 | 8.5 | 9.5 |
| **BP86** |  | 0.034 | 0.040 | 0.071 |  | 3.3 | 3.3 | 7.8 |  | –7.9 | 7.9 | 9.9 |
| **M06** |  | 0.009 | 0.010 | 0.019 |  | 1.0 | 1.0 | 2 |  | –4.3 | 4.3 | 6.8 |
| **M06-HF** |  | –0.016 | 0.017 | 0.023 |  | –1.5 | 1.5 | 2.5 |  | –5.8 | 5.8 | 8.9 |
| **M06-L** |  | 0.031 | 0.031 | 0.049 |  | 1.2 | 1.3 | 3.2 |  | –5.5 | 5.5 | 9.9 |
| **M06-2X** |  | –0.004 | 0.006 | 0.016 |  | –0.7 | 0.7 | 1.1 |  | –4.1 | 4.1 | 6.6 |
| **PBE** |  | 0.028 | 0.038 | 0.071 |  | 2.7 | 2.7 | 6.2 |  | –9.3 | 9.3 | 11.7 |
| **SSB-D** |  | 0.012 | 0.039 | 0.077 |  | 3.4 | 3.4 | 5.3 |  | –7.2 | 7.2 | 11.2 |
| **SSB-D3(BJ)** |  | 0.010 | 0.040 | 0.086 |  | 3.2 | 3.2 | 5.2 |  | –7.5 | 7.5 | 11.3 |

**Table S6.** Representative DFT bond lengths and angles (in Å and degrees) of D_2_S∙∙∙A^–^ chalcogen-bonded complexes.^[a]^

|  | **F_2_S∙∙∙F^–^** | | | | |  | **F_2_S∙∙∙Cl^–^** | | | | |
| --- | --- | --- | --- | --- | --- | --- | --- | --- | --- | --- | --- |
| **DFT** | ***r*_Ch–A_^–^** | ***r*_Ch–D_^1^** | ***r*_Ch–D_^2^** | **Θ_1_** | **Θ_2_** |  | ***r*_Ch–A_^–^** | ***r*_Ch–D_^1^** | ***r*_Ch–D_^2^** | **Θ_1_** | **Θ_2_** |
| **B3LYP** | 1.833 | 1.833 | 1.655 | 87.5 | 87.5 |  | 2.466 | 1.769 | 1.640 | 89.4 | 89.1 |
| **BLYP-D3(BJ)** | 1.873 | 1.873 | 1.693 | 88.7 | 88.7 |  | 2.458 | 1.827 | 1.678 | 90.3 | 90.7 |
| **M06** | 1.813 | 1.813 | 1.631 | 87.0 | 87.0 |  | 2.452 | 1.735 | 1.617 | 89.3 | 88.2 |
| **M06-2X** | 1.795 | 1.795 | 1.631 | 86.2 | 86.2 |  | 2.442 | 1.722 | 1.617 | 88.7 | 87.2 |
| **PBE** | 1.843 | 1.843 | 1.675 | 87.9 | 87.9 |  | 2.401 | 1.803 | 1.663 | 89.1 | 89.4 |
| **Benchmark^[b]^** | 1.806 | 1.806 | 1.637 | 86.8 | 86.8 |  | 2.441 | 1.733 | 1.623 | 89.4 | 88.0 |
|  | **Cl_2_S∙∙∙F^–^** | | | | |  | **Cl_2_S∙∙∙Cl^–^** | | | | |
|  | ***r*_Ch–A_^–^** | ***r*_Ch–D_^1^** | ***r*_Ch–D_^2^** | **Θ_1_** | **Θ_2_** |  | ***r*_Ch–A_^–^** | ***r*_Ch–D_^1^** | ***r*_Ch–D_^2^** | **Θ_1_** | **Θ_2_** |
| **B3LYP** | 1.786 | 2.476 | 2.082 | 93.9 | 93.3 |  | 2.384 | 2.384 | 2.074 | 96.3 | 96.3 |
| **BLYP-D3(BJ)** | 1.847 | 2.468 | 2.125 | 95.9 | 95.3 |  | 2.411 | 2.411 | 2.117 | 98.5 | 98.5 |
| **M06** | 1.748 | 2.477 | 2.051 | 92.1 | 93.0 |  | 2.339 | 2.339 | 2.048 | 94.4 | 94.4 |
| **M06-2X** | 1.736 | 2.461 | 2.046 | 90.9 | 91.7 |  | 2.339 | 2.339 | 2.042 | 93.2 | 93.2 |
| **PBE** | 1.823 | 2.415 | 2.083 | 94.2 | 93.5 |  | 2.364 | 2.364 | 2.077 | 96.7 | 96.7 |
| **Benchmark^[b]^** | 1.752 | 2.450 | 2.052 | 91.2 | 92.1 |  | 2.336 | 2.336 | 2.050 | 93.5 | 93.5 |

[a] Computed at ZORA-DFT/QZ4P level. [b] Computed at ZORA-CCSD(T)/BS3+.

**Table S7.** Representative DFT bond lengths and angles (in Å and degrees) of D_2_Se∙∙∙A^–^ chalcogen-bonded complexes.^[a]^

|  | **F_2_Se∙∙∙F^–^** | | | | |  | **F_2_Se∙∙∙Cl^–^** | | | | |
| --- | --- | --- | --- | --- | --- | --- | --- | --- | --- | --- | --- |
| **DFT** | ***r*_Ch–A_^–^** | ***r*_Ch–D_^1^** | ***r*_Ch–D_^2^** | **Θ_1_** | **Θ_2_** |  | ***r*_Ch–A_^–^** | ***r*_Ch–D_^1^** | ***r*_Ch–D_^2^** | **Θ_1_** | **Θ_2_** |
| **B3LYP** | 1.946 | 1.946 | 1.794 | 87.7 | 87.7 |  | 2.511 | 1.912 | 1.788 | 88.9 | 90.4 |
| **BLYP-D3(BJ)** | 1.983 | 1.983 | 1.835 | 89.5 | 89.5 |  | 2.526 | 1.956 | 1.829 | 90.8 | 92.4 |
| **M06** | 1.931 | 1.931 | 1.773 | 87.3 | 87.3 |  | 2.495 | 1.888 | 1.765 | 88.6 | 89.6 |
| **M06-2X** | 1.910 | 1.910 | 1.765 | 85.4 | 85.4 |  | 2.481 | 1.868 | 1.757 | 86.8 | 87.4 |
| **PBE** | 1.958 | 1.958 | 1.815 | 88.3 | 88.3 |  | 2.473 | 1.936 | 1.808 | 89.3 | 90.7 |
| **Benchmark^[b]^** | 1.915 | 1.915 | 1.769 | 86.5 | 86.5 |  | 2.476 | 1.874 | 1.763 | 87.8 | 88.3 |
|  | **Cl_2_Se∙∙∙F^–^** | | | | |  | **Cl_2_Se∙∙∙Cl^–^** | | | | |
|  | ***r*_Ch–A_^–^** | ***r*_Ch–D_^1^** | ***r*_Ch–D_^2^** | **Θ_1_** | **Θ_2_** |  | ***r*_Ch–A_^–^** | ***r*_Ch–D_^1^** | ***r*_Ch–D_^2^** | **Θ_1_** | **Θ_2_** |
| **B3LYP** | 1.927 | 2.520 | 2.231 | 96.1 | 93.7 |  | 2.468 | 2.468 | 2.232 | 98.0 | 98.0 |
| **BLYP-D3(BJ)** | 1.971 | 2.528 | 2.286 | 99.4 | 98.0 |  | 2.483 | 2.483 | 2.307 | 105.5 | 105.5 |
| **M06** | 1.903 | 2.504 | 2.200 | 93.8 | 92.5 |  | 2.438 | 2.438 | 2.198 | 95.3 | 95.3 |
| **M06-2X** | 1.885 | 2.494 | 2.186 | 91.2 | 89.9 |  | 2.428 | 2.428 | 2.190 | 92.5 | 92.5 |
| **PBE** | 1.952 | 2.484 | 2.237 | 96.5 | 94.9 |  | 2.452 | 2.452 | 2.241 | 99.5 | 99.5 |
| **Benchmark^[b]^** | 1.891 | 2.490 | 2.192 | 91.7 | 90.9 |  | 2.429 | 2.429 | 2.190 | 93.3 | 93.3 |

[a] Computed at ZORA-DFT/QZ4P level. [b] Computed at ZORA-CCSD(T)/BS3+.

**Table S8.** Thermodynamic values (in kcal mol^–1^ at 298 K) associated with formation of D_2_S∙∙∙A^–^ chalcogen-bonded complexes for representative methods.^[a]^

|  | **F_2_S∙∙∙F^–^** | | |  | **F_2_S∙∙∙Cl^–^** | | |
| --- | --- | --- | --- | --- | --- | --- | --- |
| **DFT** | **Δ*H*** | **TΔ*S*** | **Δ*G*** |  | **Δ*H*** | **TΔ*S*** | **Δ*G*** |
| **B3LYP** | –51.0 | –5.5 | –45.5 |  | –23.5 | –3.8 | –19.6 |
| **BLYP-D3(BJ)** | –54.5 | –5.3 | –49.2 |  | –28.9 | –3.7 | –25.2 |
| **M06** | –50.0 | –5.5 | –44.5 |  | –23.2 | –3.8 | –19.4 |
| **M06-2X** | –51.2 | –4.0 | –45.5 |  | –23.7 | –4.0 | –19.7 |
| **PBE** | –56.8 | –5.4 | –51.4 |  | –29.6 | –3.9 | –25.7 |
| **Benchmark^[b]^** | –45.7 | –5.2 | –40.5 |  | –20.5 | –3.9 | –16.6 |
|  | **Cl_2_S∙∙∙F^–^** | | |  | **Cl_2_S∙∙∙Cl^–^** | | |
|  | **Δ*H*** | **TΔ*S*** | **Δ*G*** |  | **Δ*H*** | **TΔ*S*** | **Δ*G*** |
| **B3LYP** | –53.7 | –4.5 | –49.2 |  | –25.7 | –3.8 | –21.9 |
| **BLYP-D3(BJ)** | –56.9 | –4.3 | –52.6 |  | –32.1 | –3.6 | –28.6 |
| **M06** | –52.7 | –4.5 | –48.2 |  | –25.1 | –3.9 | –21.2 |
| **M06-2X** | –53.8 | –4.8 | –49.1 |  | –24.8 | –4.1 | –20.7 |
| **PBE** | –58.2 | –4.5 | –53.6 |  | –31.2 | –3.8 | –27.4 |
| **Benchmark^[b]^** | –47.8 | –4.7 | –43.1 |  | –22.1 | –3.7 | –18.4 |

[a] Computed at ZORA-DFT/QZ4P level. [b] Computed at ZORA-CCSD(T)/BS3+.

**Table S9.** Thermodynamic values (in kcal mol^–1^ at 298 K) associated with formation of D_2_Se∙∙∙A^–^ chalcogen-bonded complexes for representative methods.

|  | **F_2_Se∙∙∙F^–^** | | |  | **F_2_Se∙∙∙Cl^–^** | | |
| --- | --- | --- | --- | --- | --- | --- | --- |
| **DFT** | **Δ*H*** | **TΔ*S*** | **Δ*G*** |  | **Δ*H*** | **TΔ*S*** | **Δ*G*** |
| **B3LYP** | –61.4 | –5.3 | –56.1 |  | –33.7 | –3.8 | –29.9 |
| **BLYP-D3(BJ)** | –64.0 | –5.1 | –58.9 |  | –38.2 | –3.6 | –34.7 |
| **M06** | –62.1 | –5.4 | –56.8 |  | –34.6 | –3.8 | –30.8 |
| **M06-2X** | –61.4 | –5.6 | –55.8 |  | –33.7 | –4.0 | –29.7 |
| **PBE** | –66.2 | –5.2 | –60.9 |  | –39.0 | –3.8 | –35.2 |
| **Benchmark^[b]^** | –57.6 | –5.1 | –52.5 |  | –32.3 | –4.0 | –28.3 |
|  | **Cl_2_Se∙∙∙F^–^** | | |  | **Cl_2_Se∙∙∙Cl^–^** | | |
|  | **Δ*H*** | **TΔ*S*** | **Δ*G*** |  | **Δ*H*** | **TΔ*S*** | **Δ*G*** |
| **B3LYP** | –61.9 | –4.4 | –57.5 |  | –34.5 | –3.6 | –30.9 |
| **BLYP-D3(BJ)** | –64.8 | –3.9 | –60.9 |  | –40.8 | –4.7 | –36.0 |
| **M06** | –63.2 | –4.5 | –58.7 |  | –35.5 | –3.8 | –31.7 |
| **M06-2X** | –61.4 | –4.7 | –56.7 |  | –33.8 | –4.1 | –29.6 |
| **PBE** | –66.1 | –4.3 | –61.8 |  | –39.5 | –3.5 | –36.0 |
| **Benchmark^[b]^** | –54.4 | –4.8 | –49.6 |  | –29.0 | –3.6 | –25.4 |

[a] Computed at ZORA-DFT/QZ4P level. [b] Computed at ZORA-CCSD(T)/BS3+.

**Table S10.** Cartesian coordinates, electronic energies, *H, TS,* and *G* (in a.u. at 298 K) for all stationary points computed at ZORA-CCSD(T) with ZORA-def2 basis sets in the gas phase using ORCA.

| **SF_2_** | **SCl_2_** |
| --- | --- |
| **Basis set = ZORA-def2-SVP**  ***E*[HF] =** -597.763880052  ***E*[MP2] =** -598.241367192  ***E*[CCSD] =** -598.264388945  ***E*[CCSD(T)] =** -598.275425859  ***H*[CCSD(T)]** = -598.26569137  **-*TS*[CCSD(T)]** = -0.02893238  ***G*[CCSD(T)]** = -598.29462375  S 0.00000000000000 -0.00000000031755 -1.90681502173278  F 0.00000000000000 1.22651411760688 -2.97323998800539  F 0.00000000000000 -1.22651411728933 -2.97323999026182 | **Basis set = ZORA-def2-SVP**  ***E*[HF] =** -1322.325986505  ***E*[MP2] =** -1322.720996573  ***E*[CCSD] =** -1322.757950388  ***E*[CCSD(T)] =** -1322.769706974  ***H*[CCSD(T)]** = -1322.76205444  **-*TS*[CCSD(T)]** = -0.03184715  ***G*[CCSD(T)]** = -1322.79390159  S 0.03105443658516 -0.00004521844648 0.14399311722788  Cl -0.26255226241268 0.00001701942546 2.19526575423164  Cl 2.09773262091264 0.00000601441673 -0.00689596673690 |
| **Basis set = ZORA-def2-TZVPP**  ***E*[HF] =** -598.471519752  ***E*[MP2] =** -599.144046177  ***E*[CCSD] =** -599.157395495  ***E*[CCSD(T)] =** -599.181543549  ***H*[CCSD(T)]** = -599.17221392  **-*TS*[CCSD(T)]** = -0.02887663  ***G*[CCSD(T)]** = -599.20109054  S 0.00000000000000 0.00000000010109 -1.91984069575599  F 0.00000000000000 1.20659101423053 -2.96672715078580  F 0.00000000000000 -1.20659101433162 -2.96672715345820 | **Basis set = ZORA-def2-TZVPP**  ***E*[HF] =** -1322.792305967  ***E*[MP2] =** -1323.342256798  ***E*[CCSD] =** -1323.385513171  ***E*[CCSD(T)] =** -1323.414254815  ***H*[CCSD(T)]** = -1323.40571386  **-*TS*[CCSD(T)]** = -0.03162412  ***G*[CCSD(T)]** = -1323.43733798  S 0.04613011938110 -0.00004425365440 0.16015122388023  Cl -0.25012776516515 0.00001644395422 2.16856172558223  Cl 2.07023244086917 0.00000562509589 0.00364995526015 |
| **Basis set = ZORA-def2-QZVPP**  ***E*[HF] =** -598.49501744  ***E*[MP2] =** -599.23164377  ***E*[CCSD] =** -599.23903133  ***E*[CCSD(T)] =** -599.267647662  ***H*[CCSD(T)]** = -599.26067879  **-*TS*[CCSD(T)]** = -0.02886327  ***G*[CCSD(T)]** = -599.28954206  S 0.14747020743221 -0.00003776829091 0.26876585029894  F -0.02021629359400 0.00001189305746 1.85247048381683  F 1.73898088124691 0.00000369062915 0.21112657060684 | **Basis set = ZORA-def2-QZVPP**  ***E*[HF] =** -1322.831987123  ***E*[MP2] =** -1323.439184066  ***E*[CCSD] =** -1323.47674212  ***E*[CCSD(T)] =** -1323.510942892  ***H*[CCSD(T)]** = -1323.5027893  **-*TS*[CCSD(T)]** = -0.03158454  ***G*[CCSD(T)]** = -1323.53437384  S 0.04803298689025 -0.00004413187706 0.16219072555150  Cl -0.24393614049400 0.00001634976047 2.16087745220763  Cl 2.06213794868887 0.00000559751230 0.00929472696348 |
| **SeF_2_** | **SeCl_2_** |
| **Basis set = ZORA-def2-SVP**  ***E*[HF] =** -2642.289878513  ***E*[MP2] =** -2642.762852586  ***E*[CCSD] =** -2642.782223911  ***E*[CCSD(T)] =** -2642.793900894  ***H*[CCSD(T)]** = -2642.78839395  **-*TS*[CCSD(T)]** = -0.03047757  ***G*[CCSD(T)]** = -2642.81887152  Se 0.00000000000000 -0.00000000012148 -1.84987476649371  F 0.00000000000000 1.31305262418678 -3.02517511618628  F 0.00000000000000 -1.31305262406532 -3.02517511732001 | **Basis set = ZORA-def2-SVP**  ***E*[HF] =** -3366.867524526  ***E*[MP2] =** -3367.253475443  ***E*[CCSD] =** -3367.287763195  ***E*[CCSD(T)] =** -3367.29964583  ***H*[CCSD(T)]** = -3367.29571263  **-*TS*[CCSD(T)]** = -0.03332676  ***G*[CCSD(T)]** = -3367.32903939  Se 0.05193531358327 -0.00000364657591 0.15563845536956  Cl -0.29909807049056 -0.00000171095910 2.32876770139258  Cl 2.25247320151071 0.00000709680766 0.09772317664789 |
| **Basis set = ZORA-def2-TZVPP**  ***E*[HF] =** -2643.353470539  ***E*[MP2] =** -2644.013207468  ***E*[CCSD] =** -2644.021138825  ***E*[CCSD(T)] =** -2644.044464052  ***H*[CCSD(T)]** = -2644.03104249  **-*TS*[CCSD(T)]** = -0.03042157  ***G*[CCSD(T)]** = -2644.06146407  Se 0.00000000000000 0.00000001234518 -1.86202630106916  F 0.00000000000000 1.29333115692167 -3.01909934400004  F 0.00000000000000 -1.29333116926686 -3.01909935493081 | **Basis set = ZORA-def2-TZVPP**  ***E*[HF] =** -3367.686570503  ***E*[MP2] =** -3368.216060187  ***E*[CCSD] =** -3368.25695534  ***E*[CCSD(T)] =** -3368.283918003  ***H*[CCSD(T)]** = -3368.27077707  **-*TS*[CCSD(T)]** = -0.03314280  ***G*[CCSD(T)]** = -3368.30391987  Se 0.06401293738935 -0.00000356377997 0.16945097807384  Cl -0.28454600444302 -0.00000168127928 2.30385726039600  Cl 2.22584351165708 0.00000698433191 0.10882109494019 |
| **Basis set = ZORA-def2-QZVPP**  ***E*[HF] =** -2643.560800864  ***E*[MP2] =** -2644.281708297  ***E*[CCSD] =** -2644.284101621  ***E*[CCSD(T)] =** -2644.31164307  ***H*[CCSD(T)]** = -2644.30337011  **-*TS*[CCSD(T)]** = -0.03040610  ***G*[CCSD(T)]** = -2644.33377621  Se 0.00000000000000 0.00000018041620 -1.86361003173942  F 0.00000000000000 1.29053282759584 -3.01830740343280  F 0.00000000000000 -1.29053300801204 -3.01830756482779 | **Basis set = ZORA-def2-QZVPP**  ***E*[HF] =** -3367.909825988  ***E*[MP2] =** -3368.49301166  ***E*[CCSD] =** -3368.528872043  ***E*[CCSD(T)] =** -3368.560883367  ***H*[CCSD(T)]** = -3368.586733227  **-*TS*[CCSD(T)]** = -0.03311411  ***G*[CCSD(T)]** = -3368.553619107  Se 0.06503608907494 -0.00000355676620 0.17062094647676  Cl -0.27892505061065 -0.00000166361718 2.29791017387388  Cl 2.21919940613912 0.00000695965605 0.11359821305939 |
| **F_2_S∙∙∙F^–^** | **Cl_2_S∙∙∙F^–^** |
| **Basis set = ZORA-def2-SVP**  ***E*[HF] =** -697.114915868  ***E*[MP2] =** -697.784674655  ***E*[CCSD] =** -697.809961837  ***E*[CCSD(T)] =** -697.823990167  ***H*[CCSD(T)]** = -697.81153846  **-*TS*[CCSD(T)]** = -0.03198485  ***G*[CCSD(T)]** = -697.84352331  S -0.02277016273259 0.00035579504278 -0.00760695355440  F 0.10247506848899 -0.00052808499286 1.77968909030768  F 1.63690412227069 -0.00086855322828 -0.01706749894183  F 0.08209097197293 0.00104084317838 -1.79621463781145 | **Basis set = ZORA-def2-SVP**  ***E*[HF] =** -1421.701610416  ***E*[MP2] =** -1422.28204642  ***E*[CCSD] =** -1422.321247048  ***E*[CCSD(T)] =** -1422.335863698  ***H*[CCSD(T)]** = -1422.32498799  **-*TS*[CCSD(T)]** = -0.03581628  ***G*[CCSD(T)]** = -1422.36080427  S -0.03764659207595 -0.00002118831274 -0.24427930356326  Cl -0.25009703799985 0.00020205240220 2.27282060310067  Cl 2.06443617515229 -0.00016776223405 -0.09277602190258  F 0.02200745492351 -0.00001310185540 -1.97696527763483 |
| **Basis set = ZORA-def2-TZVPP**  ***E*[HF] =** -698.125909638  ***E*[MP2] =** -699.091204949  ***E*[CCSD] =** -699.096567664  ***E*[CCSD(T)] =** -699.13089326  ***H*[CCSD(T)]** = -699.11887001  **-*TS*[CCSD(T)]** = -0.03226743  ***G*[CCSD(T)]** = -699.15113744  S -0.01062299722616 0.00035089324138 -0.00767627405546  F 0.09812197674835 -0.00053204005579 1.79794825557852  F 1.63367097416264 -0.00087174156154 -0.01704913582328  F 0.07753004631519 0.00105288837596 -1.81442284569979 | **Basis set = ZORA-def2-TZVPP**  ***E*[HF] =** -1422.458654848  ***E*[MP2] =** -1423.29252753  ***E*[CCSD] =** -1423.33001688  ***E*[CCSD(T)] =** -1423.36845519  ***H*[CCSD(T)]** = -1423.35690351  **-*TS*[CCSD(T)]** = -0.03578169  ***G*[CCSD(T)]** = -1423.39268520  S -0.02170489294419 0.00005673060714 -0.21377776112087  Cl -0.24296907839047 0.00001153872981 2.23332105087034  Cl 2.04241910565072 -0.00011119471488 -0.08889713628815  F 0.02095486568394 0.00004292537794 -1.97184615346132 |
| **Basis set =ZORA-def2-QZVPP**  ***E*[HF] =** -698.156003298  ***E*[MP2] =** -699.217041564  ***E*[CCSD] =** -699.213336718  ***E*[CCSD(T)] =** -699.255080898  ***H*[CCSD(T)]** = -699.243839598  **-*TS*[CCSD(T)]** = -0.0327742  ***G*[CCSD(T)]** = -699.276613798  S -0.00942637292062 -0.00154503563484 -0.00768360067331  F 0.10034533847810 0.00047691357584 1.79391771316783  F 1.62798118165056 -0.00099019348628 -0.01701747295328  F 0.07979985279198 0.00205831554529 -1.81041663954124 | **Basis set =ZORA-def2-QZVPP**  ***E*[HF] =** -1422.505478303  ***E*[MP2] =** -1423.427181099  ***E*[CCSD] =** -1423.456462873  ***E*[CCSD(T)] =** -1423.503227915  ***H*[CCSD(T)]** = -1423.492049015  **-*TS*[CCSD(T)]** = -0.0363878  ***G*[CCSD(T)]** = -1423.528436815  S -0.02195237542348 0.00022472781853 -0.21608962239125  Cl -0.23304645081413 0.00000043972072 2.22630493714301  Cl 2.02692248442286 -0.00026552727336 -0.08390925933697  F 0.02677634181475 0.00004035973412 -1.96750605541479 |
| **F_2_Se∙∙∙F^–^** | **Cl_2_Se∙∙∙F^–^** |
| **Basis set = ZORA-def2-SVP**  ***E*[HF] =** -2741.66508189  ***E*[MP2] =** -2742.328153225  ***E*[CCSD] =** -2742.350308627  ***E*[CCSD(T)] =** -2742.364634754  ***H*[CCSD(T)]** = -2742.35644671  **-*TS*[CCSD(T)]** = -0.03366268  ***G*[CCSD(T)]** = -2742.39010940  Se -0.08741640632285 -0.04593421301967 -0.00005078517359  F 0.35048601039431 1.80484893507059 0.00002968169691  F 1.67153947717401 -0.34647761035306 -0.00000860699326  F -0.28881108124547 -1.93712611169786 0.00002971046995 | **Basis set = ZORA-def2-SVP**  ***E*[HF] =** -3466.25579062  ***E*[MP2] =** -3466.829448184  ***E*[CCSD] =** -3466.865370766  ***E*[CCSD(T)] =** -3466.880195581  ***H*[CCSD(T)]** = -3466.87319722  **-*TS*[CCSD(T)]** = -0.03732989  ***G*[CCSD(T)]** = -3466.91052712  Se -0.07099998714826 -0.00026033700871 -0.20125771640814  Cl -0.28265500780144 0.00021883768999 2.31451280902901  Cl 2.17211970382583 0.00067544879203 -0.08265197576170  F -0.01976470887612 -0.00063394947331 -2.07180311685914 |
| **Basis set = ZORA-def2-TZVPP**  ***E*[HF] =** -2743.030296539  ***E*[MP2] =** -2743.977626201  ***E*[CCSD] =** -2743.979041848  ***E*[CCSD(T)] =** -2744.011684774  ***H*[CCSD(T)]** = -2743.99545545  **-*TS*[CCSD(T)]** = -0.03396100  ***G*[CCSD(T)]** = -2744.02941645  Se -0.08295550272195 -0.04673343487142 -0.00013962264581  F 0.35374308629786 1.82173078109103 0.00007463195213  F 1.66641903528610 -0.34553477156303 -0.00000966974286  F -0.29140861886201 -1.95415157465658 0.00007466043655 | **Basis set = ZORA-def2-TZVPP**  ***E*[HF] =** -3467.367792942  ***E*[MP2] =** -3468.181462278  ***E*[CCSD] =** -3468.215994542  ***E*[CCSD(T)] =** -3468.252394083  ***H*[CCSD(T)]** = -3468.23630138  **-*TS*[CCSD(T)]** = -0.03735887  ***G*[CCSD(T)]** = -3468.27366024  Se -0.05732553393800 -0.00023179025812 -0.18506088229620  Cl -0.27683479543281 0.00022626052228 2.30614900363456  Cl 2.14497618675083 0.00064655793832 -0.08456658818429  F -0.01211585738001 -0.00064102820248 -2.07772153315405 |
| **Basis set = ZORA-def2-QZVPP**  ***E*[HF] =** -2743.244096209  ***E*[MP2] =** -2744.283993316  ***E*[CCSD] =** -2744.276923098  ***E*[CCSD(T)] =** -2744.316570256  ***H*[CCSD(T)]** = -2744.305495376  **-*TS*[CCSD(T)]** = -0.034215  ***G*[CCSD(T)]** = -2744.339710376  Se -0.08187285329911 -0.04689285606522 -0.00000464137266  F 0.35446826069946 1.81760657996393 0.00000253745679  F 1.66251527537070 -0.34491285792209 -0.00000046505273  F -0.28931268277106 -1.95048986597663 0.00000256896861 | **Basis set = ZORA-def2-QZVPP**  ***E*[HF] =** -3467.597864099  ***E*[MP2] =** -3468.495971048  ***E*[CCSD] =** -3468.522971537  ***E*[CCSD(T)] =** -3468.567214143  ***H*[CCSD(T)]** = -3468.557727723  **-*TS*[CCSD(T)]** = -0.0378867  ***G*[CCSD(T)]** = -3468.595614423  Se -0.06331740775182 -0.00024239221819 -0.18622197640803  Cl -0.26244039234059 0.00023542793337 2.29776951644954  Cl 2.12699892108342 0.00063949734970 -0.07824782987739  F -0.00254112099100 -0.00063253306488 -2.07449971016410 |
| **F_2_S∙∙∙Cl^–^** | **Cl_2_S∙∙∙Cl^–^** |
| **Basis set = ZORA-def2-SVP**  ***E*[HF] =** -1059.424898769  ***E*[MP2] =** -1060.044468575  ***E*[CCSD] =** -1060.077131989  ***E*[CCSD(T)] =** -1060.090723751  ***H*[CCSD(T)]** = -1060.07884138  **-*TS*[CCSD(T)]** = -0.03422380  ***G*[CCSD(T)]** = -1060.11306518  S 0.03000925103364 -0.00127005380662 0.22032969790656  F 0.13637072756159 -0.00145555353081 1.93574463945767  F 1.66382441271506 0.00340911598151 0.11497022848503  Cl -0.03150439131028 -0.00068350864406 -2.31224456584926 | **Basis set = ZORA-def2-SVP**  ***E*[HF] =** -1783.996027063  ***E*[MP2] =** -1784.5367893  ***E*[CCSD] =** -1784.581463416  ***E*[CCSD(T)] =** -1784.597544947  ***H*[CCSD(T)]** = -1784.58735539  **-*TS*[CCSD(T)]** = -0.03756557  ***G*[CCSD(T)]** = -1784.62492096  S -0.00148457195570 -0.00006351764671 -0.02570075112352  Cl -0.23002398464876 -0.00018368188657 2.35638929618025  Cl 2.09709685003630 0.00042798735217 0.04594435174602  Cl -0.06688829343182 -0.00018078781888 -2.41783289680273 |
| **Basis set = ZORA-def2-TZVPP**  ***E*[HF] =** -1060.302463191  ***E*[MP2] =** -1061.195441399  ***E*[CCSD] =** -1061.218799045  ***E*[CCSD(T)] =** -1061.254282332  ***H*[CCSD(T)]** = -1061.24231988  **-*TS*[CCSD(T)]** = -0.03426830  ***G*[CCSD(T)]** = -1061.27658819  S 0.02966923022396 -0.00128638273815 0.18920038563658  F 0.13681030885087 -0.00143649092283 1.92450204731590  F 1.65653669742147 0.00339033369958 0.10724043124022  Cl -0.02431623649629 -0.00066746003858 -2.26214286419271 | **Basis set = ZORA-def2-TZVPP**  ***E*[HF] =** -1784.625338191  ***E*[MP2] =** -1785.398349919  ***E*[CCSD] =** -1785.449553192  ***E*[CCSD(T)] =** -1785.490984639  ***H*[CCSD(T)]** = -1785.47956942  **-*TS*[CCSD(T)]** = -0.03748402  ***G*[CCSD(T)]** = -1785.51705344  S 0.01478825542113 -0.00015803290393 -0.02515624111349  Cl -0.22699277794648 -0.00001019703160 2.31110030438892  Cl 2.07788769441437 0.00017560173355 0.04531959971544  Cl -0.06698317188900 -0.00000737179801 -2.37246366299086 |
| **Basis set = ZORA-def2-QZVPP**  ***E*[HF] =** -1060.341042325  ***E*[MP2] =** -1061.324830303  ***E*[CCSD] =** -1061.340031467  ***E*[CCSD(T)] =** -1061.383261819  ***H*[CCSD(T)]** = -1061.372033119  **-*TS*[CCSD(T)]** = -0.03480292  ***G*[CCSD(T)]** = -1061.406836039  S 0.02936161038877 -0.00102223517770 0.19057105577337  F 0.13945928303364 -0.00158725329233 1.91901902555392  F 1.64966368447717 0.00337916179643 0.10419558030230  Cl -0.01978457789957 -0.00076967332638 -2.25498566162961 | **Basis set = ZORA-def2-QZVPP**  ***E*[HF] =** -1784.679803566  ***E*[MP2] =** -1785.536993932  ***E*[CCSD] =** -1785.580081643  ***E*[CCSD(T)] =** -1785.630394439  ***H*[CCSD(T)]** = -1785.619043439  **-*TS*[CCSD(T)]** = -0.0379875  ***G*[CCSD(T)]** = -1785.657030939  S 0.00933904856951 -0.00013592015085 -0.02533977401678  Cl -0.21420531421644 -0.00006427855066 2.30115965258418  Cl 2.05846958362367 0.00026164605579 0.04465549338881  Cl -0.05490331797673 -0.00006144735426 -2.36167537195620 |
| **F_2_Se∙∙∙Cl^–^** | **Cl_2_Se∙∙∙Cl^–^** |
| **Basis set = ZORA-def2-SVP**  ***E*[HF] =** -3103.970342114  ***E*[MP2] =** -3104.585883209  ***E*[CCSD] =** -3104.614878114  ***E*[CCSD(T)] =** -3104.629029485  ***H*[CCSD(T)]** = -3104.62135096  **-*TS*[CCSD(T)]** = -0.03562300  ***G*[CCSD(T)]** = -3104.65697396  Se -0.01881230502721 0.12423492200961 -0.00007984362835  F 0.39682385879790 1.93592294994524 0.00004752740320  F 1.71915368376740 -0.22554292376496 -0.00000260699023  Cl -0.45136723753810 -2.35930394818988 0.00003492321537 | **Basis set = ZORA-def2-SVP**  ***E*[HF] =** -3828.555481423  ***E*[MP2] =** -3829.08535381  ***E*[CCSD] =** -3829.127684921  ***E*[CCSD(T)] =** -3829.142958145  ***H*[CCSD(T)]** = -3829.13647969  **-*TS*[CCSD(T)]** = -0.03912289  ***G*[CCSD(T)]** = -3829.17560258  Se -0.09400426307604 0.01070630275723 0.00080725327955  Cl 0.09847092130612 2.46406688013145 0.00149863768695  Cl 2.12089699377291 -0.31313667438679 -0.00298753517988  Cl -0.61213065200298 -2.39503650850190 0.00068164421341 |
| **Basis set = ZORA-def2-TZVPP**  ***E*[HF] =** -3105.204927004  ***E*[MP2] =** -3106.082004695  ***E*[CCSD] =** -3106.100836406  ***E*[CCSD(T)] =** -3106.134900935  ***H*[CCSD(T)]** = -3106.11865504  **-*TS*[CCSD(T)]** = -0.03580262  ***G*[CCSD(T)]** = -3106.15445765  Se -0.02216449782643 0.10885672498171 0.00000503244758  F 0.39996943542697 1.93780228802050 -0.00000297014990  F 1.71270737233970 -0.22717035681671 0.00000017612582  Cl -0.44471430994024 -2.34417765618549 -0.00000223842351 | **Basis set = ZORA-def2-TZVPP**  ***E*[HF] =** -3829.539216591  ***E*[MP2] =** -3830.287297998  ***E*[CCSD] =** -3830.337476065  ***E*[CCSD(T)] =** -3830.37594639  ***H*[CCSD(T)]** = -3830.35985436  **-*TS*[CCSD(T)]** = -0.03906673  ***G*[CCSD(T)]** = -3830.39892109  Se -0.08093674748300 0.00879596485391 0.00076927120399  Cl 0.09982360244557 2.44071115911081 0.00148926044961  Cl 2.09845820435057 -0.30988725241411 -0.00293839043593  Cl -0.60411205931313 -2.37301987155061 0.00067985878236 |
| **Basis set = ZORA-def2-QZVPP**  ***E*[HF] =** -3105.426817099  ***E*[MP2] =** -3106.391821278  ***E*[CCSD] =** -3106.402957028  ***E*[CCSD(T)] =** -3106.444388172  ***H*[CCSD(T)]** = -3106.433574572  **-*TS*[CCSD(T)]** = -0.0364215  ***G*[CCSD(T)]** = -3106.469996072  Se -0.02438992085511 0.10886383915030 -0.00001296822102  F 0.40226949495688 1.93283700078296 0.00000768805325  F 1.70535579200528 -0.23040191728939 -0.00000053031022  Cl -0.43743736610704 -2.33598792264386 0.00000581047797 | **Basis set = ZORA-def2-QZVPP**  ***E*[HF] =** -3829.777099362  ***E*[MP2] =** -3830.605504047  ***E*[CCSD] =** -3830.648533388  ***E*[CCSD(T)] =** -3830.695281619  ***H*[CCSD(T)]** = -3830.685387098  **-*TS*[CCSD(T)]** = -0.0396541  ***G*[CCSD(T)]** = -3830.725041198  Se -0.09316206867494 0.01051796504255 0.00086106828818  Cl 0.11726504579773 2.43170873200308 0.00143854858367  Cl 2.07375051368660 -0.30644898031759 -0.00293061246989  Cl -0.58462049080937 -2.36917771672804 0.00063099559808 |

**Table S11.** Cartesian coordinates, electronic energies, *H, TS,* and *G* (in a.u. at 298 K) for all stationary points computed at ZORA-CCSD(T) with ma-ZORA-def2 basis sets in the gas phase using ORCA.

| **SF_2_** | **SCl_2_** |
| --- | --- |
| **Basis set = ma-ZORA-def2-SVP**  ***E*[HF] =** -597.792398897  ***E*[MP2] =** -598.286876991  ***E*[CCSD] =** -598.308213054  ***E*[CCSD(T)] =** -598.321582477  ***H*[CCSD(T)]** = -598.31218408  **-*TS*[CCSD(T)]** = -0.02893967  ***G*[CCSD(T)]** = -598.34112376  S 0.00000000000000 0.00000000240498 -1.90128007425467  F 0.00000000000000 1.23419567052634 -2.97600746055199  F 0.00000000000000 -1.23419567293132 -2.97600746519332 | **Basis set = ma-ZORA-def2-SVP**  ***E*[HF]** = -1322.33862934  ***E*[MP2] =** -1322.742466861  ***E*[CCSD] =** -1322.779398102  ***E*[CCSD(T)] =** -1322.792618378  ***H*[CCSD(T)]** = -1322.78500302  **-*TS*[CCSD(T)]** = -0.03183318  ***G*[CCSD(T)]** = -1322.81683620  S 0.03054309445629 -0.00004525117042 0.14344506710386  Cl -0.26243104705544 0.00001703641424 2.19566522444274  Cl 2.09812274768427 0.00000603015189 -0.00674738682399 |
| **Basis set = ma-ZORA-def2-TZVPP**  ***E*[HF] =** -598.473320725  ***E*[MP2] =** -599.147415238  ***E*[CCSD] =** -599.160531797  ***E*[CCSD(T)] =** -599.184957666  ***H*[CCSD(T)]** = -599.17562456  **-*TS*[CCSD(T)]** = -0.02887817  ***G*[CCSD(T)]** = -599.20450273  S 0.00000000000000 0.00000000337041 -1.92049542936963  F 0.00000000000000 1.20542487338781 -2.96639978256208  F 0.00000000000000 -1.20542487675822 -2.96639978806828 | **Basis set = ma-ZORA-def2-TZVPP**  ***E*[HF] =** -1322.794079785  ***E*[MP2] =** -1323.345043837  ***E*[CCSD] =** -1323.388153701  ***E*[CCSD(T)] =** -1323.41716677  ***H*[CCSD(T)]** = -1323.40862974  **-*TS*[CCSD(T)]** = -0.03162379  ***G*[CCSD(T)]** = -1323.44025352  S 0.04664218514125 -0.00004422088410 0.16070005100182  Cl -0.24966927195861 0.00001642423762 2.16762065680590  Cl 2.06926188190248 0.00000561204219 0.00404219691489 |
| **Basis set =ma-ZORA-def2-QZVPP**  ***E*[HF] =** -598.495306088  ***E*[MP2] =** -599.232277981  ***E*[CCSD] =** -599.239594714  ***E*[CCSD(T)]** = -599.268271232  ***H*[CCSD(T)]** = -599.26129265  **-*TS*[CCSD(T)]** = -0.02886416  ***G*[CCSD(T)]** = -599.29015680  S 0.14746696196643 -0.00003776856143 0.26876053739465  F -0.02021570478323 0.00001189322297 1.85247484726701  F 1.73898353790192 0.00000369073417 0.21112752006095 | **Basis set =ma-ZORA-def2-QZVPP**  ***E*[HF] =** -1322.832199297  ***E*[MP2] =** -1323.439551599  ***E*[CCSD] =** -1323.477058488  ***E*[CCSD(T)] =** -1323.511293853  ***H*[CCSD(T)]** = -1323.50514211  **-*TS*[CCSD(T)]** = -0.03158496  ***G*[CCSD(T)]** = --1323.53672707  S 0.04795473169107 -0.00004413688448 0.16210687059975  Cl -0.24386293926722 0.00001635210506 2.16088758158132  Cl 2.06214300266126 0.00000560017513 0.00936845254154 |
| **SeF_2_** | **SeCl_2_** |
| **Basis set = ma-ZORA-def2-SVP**  ***E*[HF] =** -2642.31758699  ***E*[MP2] =** -2642.807565962  ***E*[CCSD] =** -2642.82445906  ***E*[CCSD(T)] =** -2642.838529662  ***H*[CCSD(T)]** = -2642.83352563  **-*TS*[CCSD(T)]** = -0.03052529  ***G*[CCSD(T)]** = -2642.86405092  Se 0.00000000000000 0.00000002200343 -1.84764096261793  F 0.00000000000000 1.32092963227680 -3.02629200821785  F 0.00000000000000 -1.32092965428024 -3.02629202916422 | **Basis set = ma-ZORA-def2-SVP**  ***E*[HF] =** -3366.879935067  ***E*[MP2] =** -3367.274425401  ***E*[CCSD] =** -3367.308485842  ***E*[CCSD(T)] =** -3367.321734338  ***H*[CCSD(T)]** = -3367.31785593  **-*TS*[CCSD(T)]** = -0.03330636  ***G*[CCSD(T)]** = -3367.35116229  Se 0.05184804526477 -0.00000364717436 0.15553853555006  Cl -0.29874096479794 -0.00000170957773 2.32854360920711  Cl 2.25220336413659 0.00000709602474 0.09804718865286 |
| **Basis set = ma-ZORA-def2-TZVPP**  ***E*[HF] =** -2643.355369875  ***E*[MP2] =** -2644.016680179  ***E*[CCSD] =** -2644.024337486  ***E*[CCSD(T)] =** -2644.047948904  ***H*[CCSD(T)]** = -2644.03452806  **-*TS*[CCSD(T)]** = -0.03042507  ***G*[CCSD(T)]** = -2644.06495313  Se 0.00000000000000 0.00000029037081 -1.86353548865363  F 0.00000000000000 1.29057008186767 -3.01834462583529  F 0.00000000000000 -1.29057037223849 -3.01834488551109 | **Basis set = ma-ZORA-def2-TZVPP**  ***E*[HF] =** -3367.688350719  ***E*[MP2] =** -3368.218803092  ***E*[CCSD] =** -3368.259543178  ***E*[CCSD(T)] =** -3368.286769181  ***H*[CCSD(T)]** = -3368.27363249  **-*TS*[CCSD(T)]** = -0.03314409  ***G*[CCSD(T)]** = -3368.30677659  Se 0.06408927423125 -0.00000356325666 0.16953827958603  Cl -0.28288297659212 -0.00000167566858 2.30232611727161  Cl 2.22410414696428 0.00000697819789 0.11026493655239 |
| **Basis set = ma-ZORA-def2-QZVPP**  ***E*[HF] =** -2643.561177049  ***E*[MP2] =** -2644.282505226  ***E*[CCSD] =** -2644.284810172  ***E*[CCSD(T)] =** -2644.312439179  ***H*[CCSD(T)]** = -2644.304167539  **-*TS*[CCSD(T)]** = -0.03040729  ***G*[CCSD(T)]** = -2644.334574819  Se 0.00000000000000 0.00000028826563 -1.86376843572374  F 0.00000000000000 1.29063501738524 -3.01822815316879  F 0.00000000000000 -1.29063530565087 -3.01822841110748 | **Basis set = ma-ZORA-def2-QZVPP**  ***E*[HF] =** -3367.910001749  ***E*[MP2] =** -3368.493349671  ***E*[CCSD] =** -3368.529165164  ***E*[CCSD(T)] =** -3368.56121542  ***H*[CCSD(T)]** = -3368.56027121  **-*TS*[CCSD(T)]** = -0.0331151  ***G*[CCSD(T)]** = -3368.59336  Se 0.06503249368997 -0.00000355679080 0.17061686475935  Cl -0.27888467274864 -0.00000166347173 2.29787847003217  Cl 2.21916262366208 0.00000695953519 0.11363399861851 |
| **F_2_S∙∙∙F^–^** | **Cl_2_S∙∙∙F^–^** |
| **Basis set = ma-ZORA-def2-SVP**  ***E*[HF] =** -697.191331953  ***E*[MP2] =** -697.9016235  ***E*[CCSD] =** -697.920741444  ***E*[CCSD(T)] =** -697.940931025  ***H*[CCSD(T)]** = -697.92891588  **-*TS*[CCSD(T)]** = -0.03250411  ***G*[CCSD(T)]** = -697.96142000  S -0.01152863368111 0.00049934400026 -0.00767082649706  F 0.08211264527603 -0.00041723447089 1.84557708164708  F 1.66713772760237 -0.00129150218806 -0.01723939119627  F 0.06097826080273 0.00120939265869 -1.86186686395375 | **Basis set = ma-ZORA-def2-SVP**  ***E*[HF] =** -1421.753718816  ***E*[MP2] =** -1422.36182771  ***E*[CCSD] =** -1422.398400479  ***E*[CCSD(T)] =** -1422.417666477  ***H*[CCSD(T)]** = -1422.40702135  **-*TS*[CCSD(T)]** = -0.03645455  ***G*[CCSD(T)]** = -1422.44347590  S -0.02019779917366 -0.00023760872617 -0.21643479440729  Cl -0.27144399457853 -0.00035720283612 2.29654720732163  Cl 2.08315066549720 0.00083730838519 -0.10132717196878  F 0.00719112825499 -0.00024249682289 -2.01998524094557 |
| **Basis set = ma-ZORA-def2-TZVPP**  ***E*[HF] =** -698.132045623  ***E*[MP2] =** -699.103298373  ***E*[CCSD] =** -699.107233905  ***E*[CCSD(T)] =** -699.14287274  ***H*[CCSD(T)]** = -699.13088345  **-*TS*[CCSD(T)]** = -0.03230925  ***G*[CCSD(T)]** = -699.16319270  S -0.00994186600220 0.00030307588982 -0.00767980337016  F 0.09967886653510 -0.00050780984216 1.79803564667100  F 1.62987649939243 -0.00087246872389 -0.01702825243406  F 0.07908650007469 0.00107720267624 -1.81452759086678 | **Basis set = ma-ZORA-def2-TZVPP**  ***E*[HF] =** -1422.464525549  ***E*[MP2] =** -1423.30261018  ***E*[CCSD] =** -1423.339150449  ***E*[CCSD(T)] =** -1423.378695585  ***H*[CCSD(T)]** = -1423.36715825  **-*TS*[CCSD(T)]** = -0.03583811  ***G*[CCSD(T)]** = -1423.40299636  S -0.01985739628822 -0.00009950539576 -0.21637030967554  Cl -0.24142188636821 0.00008779649589 2.23392487564073  Cl 2.03855420407126 -0.00016915980429 -0.08771123819707  F 0.02142507858517 0.00018086870416 -1.97104332776813 |
| **Basis set = ma-ZORA-def2-QZVPP**  ***E*[HF] =** -698.157359987  ***E*[MP2] =** -699.220069248  ***E*[CCSD] =** -699.215937757  ***E*[CCSD(T)] =** -699.258089478  ***H*[CCSD(T)]** = -699.246848178  **-*TS*[CCSD(T)]** = -0.032900715  ***G*[CCSD(T)]** = -699.279748893  S -0.00931975349779 0.00023979945472 -0.00773187404433  F 0.10042044655865 -0.00047349245276 1.79440677472209  F 1.62771939278189 -0.00087461601943 -0.01703096525971  F 0.07987991415727 0.00110830901748 -1.81084393541805 | **Basis set = ma-ZORA-def2-QZVPP**  ***E*[HF] =** -1422.506480127  ***E*[MP2] =** -1423.42920219  ***E*[CCSD] =** -1423.458159279  ***E*[CCSD(T)] =** -1423.505197817  ***H*[CCSD(T)]** = -1423.494064607  **-*TS*[CCSD(T)]** = -0.03644117  ***G*[CCSD(T)]** = -1423.530505777  S -0.02159959841418 -0.00005576553709 -0.21575142023777  Cl -0.23254760396175 0.00011389402469 2.22511697820195  Cl 2.02646927091325 -0.00025995700514 -0.08312779315625  F 0.02637793146268 0.00020182851754 -1.96743776480793 |
| **F_2_Se∙∙∙F^–^** | **Cl_2_Se∙∙∙F^–^** |
| **Basis set = ma-ZORA-def2-SVP**  ***E*[HF] =** -2741.739867874  ***E*[MP2] =** -2742.442046103  ***E*[CCSD] =** -2742.457516839  ***E*[CCSD(T)] =** -2742.47773522  ***H*[CCSD(T)]** = -2742.46988806  **-*TS*[CCSD(T)]** = -0.03414569  ***G*[CCSD(T)]** = -2742.50403375  Se -0.07702523575222 -0.04772242559895 0.00005810380659  F 0.33666762148867 1.86363708562300 -0.00003155815760  F 1.70755243552697 -0.35259654199076 0.00000498042602  F -0.32139682126343 -1.98800711803329 -0.00003152607500 | **Basis set = ma-ZORA-def2-SVP**  ***E*[HF] =** -3466.306834762  ***E*[MP2] =** -3466.907526176  ***E*[CCSD] =** -3466.940247275  ***E*[CCSD(T)] =** -3466.959497647  ***H*[CCSD(T)]** = -3466.95266180  **-*TS*[CCSD(T)]** = -0.03762158  ***G*[CCSD(T)]** = -3466.99028337  Se -0.05779937965250 -0.00020398455046 -0.17630158476616  Cl -0.29664600987941 0.00020277593635 2.33630350497410  Cl 2.18317913020187 0.00058896941166 -0.08620755057560  F -0.03003374066995 -0.00058776079754 -2.11499436963231 |
| **Basis set = ma-ZORA-def2-TZVPP**  ***E*[HF] =** -2743.036135722  ***E*[MP2] =** -2743.989207913  ***E*[CCSD] =** -2743.989188316  ***E*[CCSD(T)] =** -2744.023109164  ***H*[CCSD(T)]** = -2744.00690943  **-*TS*[CCSD(T)]** = -0.03400293  ***G*[CCSD(T)]** = -2744.04091236  Se -0.08378408648530 -0.04656789310180 0.00020463322434  F 0.35683207323100 1.81943144822165 -0.00010974645134  F 1.66044933062171 -0.34454930755658 0.00001482815496  F -0.28769931736742 -1.95300324756327 -0.00010971492795 | **Basis set = ma-ZORA-def2-TZVPP**  ***E*[HF] =** -3467.373087613  ***E*[MP2] =** -3468.191044206  ***E*[CCSD] =** -3468.224541892  ***E*[CCSD(T)] =** -3468.262037049  ***H*[CCSD(T)]** = -3468.24596185  **-*TS*[CCSD(T)]** = -0.03738831  ***G*[CCSD(T)]** = -3468.28335015  Se -0.06320626951979 0.00691641737147 -0.18533540001394  Cl -0.26799179621663 -0.00277789713958 2.30184329236320  Cl 2.13443708834967 0.00046835458849 -0.08205474759484  F -0.00453902261324 -0.00460687482038 -2.07565314475440 |
| **Basis set = ma-ZORA-def2-QZVPP**  ***E*[HF] =** -2743.245547154  ***E*[MP2] =** -2744.287185647  ***E*[CCSD] =** -2744.279674009  ***E*[CCSD(T)] =** -2744.319777487  ***H*[CCSD(T)]** = -2744.308693387  **-*TS*[CCSD(T)]** = -0.0345901  ***G*[CCSD(T)]** = -2744.343283487  Se -0.08211060509964 -0.04685229966989 0.00001099177273  F 0.35486351101994 1.81768420561091 -0.00000582894804  F 1.66201264267343 -0.34482506571461 0.00000063464552  F -0.28896754859374 -1.95069584022642 -0.00000579747021 | **Basis set = ma-ZORA-def2-QZVPP**  ***E*[HF] =** -3467.598763057  ***E*[MP2] =** -3468.497961369  ***E*[CCSD] =** -3468.52463208  ***E*[CCSD(T)] =** -3468.569170168  ***H*[CCSD(T)]** = -3468.559695647  **-*TS*[CCSD(T)]** = -0.0378735  ***G*[CCSD(T)]** = -3468.597569147  Se -0.06377343375586 -0.00041115920619 -0.18522865888484  Cl -0.26086181438428 0.00030630028524 2.29632161861232  Cl 2.12520169851929 0.00064350338123 -0.07724428990921  F -0.00186645037914 -0.00053864446028 -2.07504866981825 |
| **F_2_S∙∙∙Cl^–^** | **Cl_2_S∙∙∙Cl^–^** |
| **Basis set = ma-ZORA-def2-SVP**  ***E*[HF] =** -1059.485208495  ***E*[MP2] =** -1060.137033774  ***E*[CCSD] =** -1060.166296215  ***E*[CCSD(T)] =** -1060.185040492  ***H*[CCSD(T)]** = -1060.17344966  **-*TS*[CCSD(T)]** = -0.03457717  ***G*[CCSD(T)]** = -1060.20802683  S 0.02595678448040 -0.00110423830920 0.19277574070541  F 0.12295217883214 -0.00160692283114 1.97266844161078  F 1.69022123724884 0.00349167422509 0.11027834839375  Cl -0.04043020056136 -0.00078051308474 -2.31692253070996 | **Basis set = ma-ZORA-def2-SVP**  ***E*[HF] =** -1784.034215735  ***E*[MP2] =** -1784.596659458  ***E*[CCSD] =** -1784.639776448  ***E*[CCSD(T)] =** -1784.659910002  ***H*[CCSD(T)]** = -1784.64983511  **-*TS*[CCSD(T)]** = -0.03821706  ***G*[CCSD(T)]** = -1784.68805216  S 0.03273301640862 -0.00002666954677 -0.02454129984864  Cl -0.26830540011606 -0.00029532582965 2.37214326317120  Cl 2.13830717801714 0.00061440024640 0.04738510471711  Cl -0.10403479430969 -0.00029240486997 -2.43618706803966 |
| **Basis set = ma-ZORA-def2-TZVPP**  ***E*[HF] =** -1060.308275252  ***E*[MP2] =** -1061.205833958  ***E*[CCSD] =** -1061.228219636  ***E*[CCSD(T)] =** -1061.264832466  ***H*[CCSD(T)]** = -1061.25288736  **-*TS*[CCSD(T)]** = -0.03431770  ***G*[CCSD(T)]** = -1061.28720507  S 0.02869224931582 -0.00145707288236 0.19059695299056  F 0.13859318050237 -0.00133048153596 1.92271163037927  F 1.65245490947549 0.00337521067270 0.10464160272880  Cl -0.02104033929367 -0.00058765625436 -2.25915018609865 | **Basis set = ma-ZORA-def2-TZVPP**  ***E*[HF] =** -1784.630586139  ***E*[MP2] =** -1785.407238014  ***E*[CCSD] =** -1785.457534865  ***E*[CCSD(T)] =** -1785.500021456  ***H*[CCSD(T)]** = -1785.48862003  **-*TS*[CCSD(T)]** = -0.03754372  ***G*[CCSD(T)]** = -1785.52616375  S 0.01383879232242 -0.00032161803804 -0.02518462387039  Cl -0.22401638574590 0.00003463775943 2.30921471685438  Cl 2.07301866400379 0.00024949464036 0.04515168112735  Cl -0.06414107058030 0.00003748563827 -2.37038177411134 |
| **Basis set = ma-ZORA-def2-QZVPP**  ***E*[HF] =** -1060.342057078  ***E*[MP2] =** -1061.327102034  ***E*[CCSD] =** -1061.341942033  ***E*[CCSD(T)] =** -1061.385487102  ***H*[CCSD(T)]** = -1061.374258402  **-*TS*[CCSD(T)]** = -0.0348866  ***G*[CCSD(T)]** = -1061.409145002  S 0.02808586857875 -0.00139816955887 0.18926982581687  F 0.14013250324361 -0.00136066261795 1.91811740902621  F 1.64876503135008 0.00336592161701 0.10286400083652  Cl -0.01828340317242 -0.00060708944018 -2.25145123567962 | **Basis set = ma-ZORA-def2-QZVPP**  ***E*[HF] =** -1784.680653798  ***E*[MP2] =** -1785.538557811  ***E*[CCSD] =** -1785.581364668  ***E*[CCSD(T)] =** -1785.631878468  ***H*[CCSD(T)]** = -1785.620527468  **-*TS*[CCSD(T)]** = -0.0381343  ***G*[CCSD(T)]** = -1785.658661768  S 0.00929669357455 -0.00007004081391 -0.02534113796889  Cl -0.21397992738411 -0.00009508533710 2.29996770484633  Cl 2.05814417225589 0.00025738146156 0.04464562583126  Cl -0.05476093844632 -0.00009225531053 -2.36047219270869 |
| **F_2_Se∙∙∙Cl^–^** | **Cl_2_Se∙∙∙Cl^–^** |
| **Basis set = ma-ZORA-def2-SVP**  ***E*[HF] =** -3104.029055955  ***E*[MP2] =** -3104.676552613  ***E*[CCSD] =** -3104.701216657  ***E*[CCSD(T)] =** -3104.720435624  ***H*[CCSD(T)]** = -3104.71301331  **-*TS*[CCSD(T)]** = -0.03597643  ***G*[CCSD(T)]** = -3104.74898974  Se -0.02537792657679 0.10156951990009 0.00010894881924  F 0.38663300684745 1.97919713257350 -0.00006308030579  F 1.74705897843725 -0.23839695114400 0.00000253363985  Cl -0.46251605870789 -2.36705870132958 -0.00004840215333 | **Basis set = ma-ZORA-def2-SVP**  ***E*[HF] =** -3828.594062151  ***E*[MP2] =** -3829.144041333  ***E*[CCSD] =** -3829.184954452  ***E*[CCSD(T)] =** -3829.203750117  ***H*[CCSD(T)]** = -3829.19733508  **-*TS*[CCSD(T)]** = -0.03937125  ***G*[CCSD(T)]** = -3829.23670633  Se -0.07908955496946 0.00853401542376 0.00080747400931  Cl 0.08332536178131 2.47931514394226 0.00157879104424  Cl 2.13998123464525 -0.31595287040420 -0.00314420722963  Cl -0.63098404145708 -2.40529628896182 0.00075794217612 |
| **Basis set = ma-ZORA-def2-TZVPP**  ***E*[HF] =** -3105.210259872  ***E*[MP2] =** -3106.091967604  ***E*[CCSD] =** -3106.109734333  ***E*[CCSD(T)] =** -3106.144918723  ***H*[CCSD(T)]** = -3106.12868790  **-*TS*[CCSD(T)]** = -0.03582787  ***G*[CCSD(T)]** = -3106.16451577  Se -0.02835058219351 0.10888972645757 0.00003228917100  F 0.40659504052812 1.93356738564527 -0.00001920211783  F 1.70210109867344 -0.22979507220432 0.00000143225453  Cl -0.43454755700804 -2.33735103989851 -0.00001451930772 | **Basis set = ma-ZORA-def2-TZVPP**  ***E*[HF] =** -3829.544099363  ***E*[MP2] =** -3830.295576554  ***E*[CCSD] =** -3830.344918622  ***E*[CCSD(T)] =** -3830.384360931  ***H*[CCSD(T)]** = -3830.36827780  **-*TS*[CCSD(T)]** = -0.03909701  ***G*[CCSD(T)]** = -3830.40737480  Se -0.09246587563093 0.01046354355087 0.00081402627955  Cl 0.11377501814161 2.43721289529819 0.00146933105167  Cl 2.08167245646689 -0.30742457250405 -0.00294372103916  Cl -0.58974859897755 -2.37365186634500 0.00066036370798 |
| **Basis set = ma-ZORA-def2-QZVPP**  ***E*[HF] =** -3105.427900605  ***E*[MP2] =** -3106.394182674  ***E*[CCSD] =** -3106.404976525  ***E*[CCSD(T)] =** -3106.446748594  ***H*[CCSD(T)]** = -3106.435936494  **-*TS*[CCSD(T)]** = -0.0364111  ***G*[CCSD(T)]** = -3106.472347594  Se -0.02707278840205 0.10837455717131 0.00004394085733  F 0.40460683166111 1.93220398628878 -0.00002609567624  F 1.70227573229622 -0.23149152116773 0.00000192059845  Cl -0.43401177555527 -2.33377602229236 -0.00001976577956 | **Basis set = ma-ZORA-def2-QZVPP**  ***E*[HF] =** -3829.777834576  ***E*[MP2] =** -3830.606917526  ***E*[CCSD] =** -3830.649702729  ***E*[CCSD(T)] =** -3830.696649821  ***H*[CCSD(T)]** = -3830.686675208  **-*TS*[CCSD(T)]** = -0.0397001  ***G*[CCSD(T)]** = -3830.726375308  Se -0.09329463771378 0.01056462950705 0.00337375363095  Cl 0.11749079187135 2.43006862951851 0.00025775124445  Cl 2.07316467627356 -0.30619014031292 -0.00308235200666  Cl -0.58412783043112 -2.36784311871264 -0.00054915286871 |

**Table S12.** Cartesian coordinates, bonding energies, *H, TS,* and *G* (in a.u. at 298 K) for all stationary points computed at ZORA-B3LYP/QZ4P in the gas phase using ADF.

| **SF_2_** | **SCl_2_** |
| --- | --- |
| ***E =*** -0.53569500  ***H =*** -0.526956374789431  ***-TS =*** -0.029318928255989  ***G =*** -0.556275303045421  S 0.249444 0.195389 0.000000  F 0.187788 1.800172 0.000000  F 1.845633 0.019548 0.000000 | ***E =*** -0.37602146  ***H =*** -0.368501683227408  ***-TS =*** -0.032063197327365  ***G =*** -0.400564880554773  S 0.177652 0.163724 0.000000  Cl 0.020687 2.196718 0.000000  Cl 2.186918 -0.179554 0.000000 |
| **SeF_2_** | **SeCl_2_** |
| ***E =*** -0.51110517  ***H =*** -0.502965463369913  ***-TS =*** -0.030912114659390  ***G =*** -0.533877578029302  Se 0.167514 0.078280 0.000000  F 0.110142 1.827031 0.000000  F 1.911514 -0.067928 0.000000 | ***E =*** -0.35877294  ***H =*** -0.351561488433860  ***-TS =*** -0.033618418328409  ***G =*** -0.385179906762269  Se 0.120867 0.063378 0.000000  Cl -0.033752 2.235442 0.000000  Cl 2.275932 -0.246106 0.000000 |
| **F_2_S∙∙∙F^–^** | **Cl_2_S∙∙∙F^–^** |
| ***E =*** -0.82395095  ***H =*** -0.812520496523375  ***-TS =*** -0.032969276660324  ***G =*** -0.845489773183699  S 0.000000 0.000000 -0.013616  F 0.000000 1.831277 0.066433  F 0.000000 0.000000 1.641123  F 0.000000 -1.831277 0.066433 | ***E =*** -0.66883921  ***H =*** -0.658367467200417  ***-TS =*** -0.037218228704924  ***G =*** -0.695585695905341  S 0.002267 0.203307 0.000000  Cl -0.280875 -2.256044 0.000000  Cl 2.082209 0.108215 0.000000  F -0.019693 1.989403 0.000000 |
| **F_2_Se∙∙∙F^–^** | **Cl_2_Se∙∙∙F^–^** |
| ***E =*** -0.81607636  ***H =*** -0.805124350237976  ***-TS =*** -0.034764472375443  ***G =*** -0.839888822613419  Se 0.000000 0.000000 -0.486402  F 0.000000 -1.944073 -0.407400  F 0.000000 0.000000 1.307892  F 0.000000 1.944073 -0.407400 | ***E =*** -0.66466657  ***H =*** -0.654478030579991  ***-TS =*** -0.038989516935611  ***G =*** -0.693467547515602  Se 0.018047 -0.090010 0.000000  Cl 0.015271 2.430331 0.000000  Cl 2.236885 -0.324136 0.000000  F -0.308252 -1.988771 0.000000 |
| **F_2_S∙∙∙Cl^–^** | **Cl_2_S∙∙∙Cl^–^** |
| ***E =*** -0.75096027  ***H =*** -0.739867359460986  ***-TS =*** -0.035560180286612  ***G =*** -0.775427539747598  S -0.008236 0.134737 0.000000  F 0.344765 1.867796 0.000000  F 1.602208 -0.176673 0.000000  Cl -0.439837 -2.293584 0.000000 | ***E =*** -0.59505704  ***H =*** -0.584908624635141  ***-TS =*** -0.038387348604287  ***G =*** -0.623295973239428  S 0.000000 0.000000 -0.387177  Cl 0.000000 -2.369892 -0.649597  Cl 0.000000 0.000000 1.686381  Cl 0.000000 2.369892 -0.649597 |
| **F_2_Se∙∙∙Cl^–^** | **Cl_2_Se∙∙∙Cl^–^** |
| ***E =*** -0.74279152  ***H =*** -0.732139993525887  ***-TS =*** -0.037211364430890  ***G =*** -0.769351357956777  Se -0.024981 0.126013 0.000000  F 0.338759 2.002947 0.000000  F 1.736318 -0.180928 0.000000  Cl -0.471167 -2.344762 0.000000 | ***E =*** -0.59199906  ***H =*** -0.582060516982489  ***-TS =*** -0.040200165116368  ***G =*** -0.622260682098857  Se 0.000000 0.000000 -0.385450  Cl 0.000000 -2.443874 -0.730512  Cl 0.000000 0.000000 1.846755  Cl 0.000000 2.443874 -0.730512 |

**Table S13.** Cartesian coordinates, bonding energies, *H, TS,* and *G* (in a.u. at 298 K) for all stationary points computed at ZORA-B3LYP-D3(BJ)/QZ4P in the gas phase using ADF.

| **SF_2_** | **SCl_2_** |
| --- | --- |
| ***E =*** -0.53751912  ***H =*** -0.528744358702172  ***-TS =*** -0.029318261837260  ***G =*** -0.558062620539432  S 0.000000 0.000000 -1.940378  F 0.000000 1.216654 -2.987840  F 0.000000 -1.216654 -2.987840 | ***E =*** -0.38211803  ***H =*** -0.374585793321928  ***-TS =*** -0.032051619991102  ***G =*** -0.406637413313030  S 0.000000 0.000000 -1.824415  Cl 0.000000 1.603741 -3.081213  Cl 0.000000 -1.603741 -3.081213 |
| **SeF_2_** | **SeCl_2_** |
| ***E =*** -0.51312548  ***H =*** -0.504962063697121  ***-TS =*** -0.030915443656473  ***G =*** -0.535877507353594  Se 0.000000 0.000000 -1.861263  F 0.000000 1.309792 -3.021715  F 0.000000 -1.309792 -3.021715 | ***E =*** -0.36522646  ***H =*** -0.358009695360797  ***-TS =*** -0.033619255938308  ***G =*** -0.391628951299105  Se 0.000000 0.000000 -1.774124  Cl 0.000000 1.689700 -3.146177  Cl 0.000000 -1.689700 -3.146177 |
| **F_2_S∙∙∙F^–^** | **Cl_2_S∙∙∙F^–^** |
| ***E =*** -0.82685979  ***H =*** -0.815426888070528  ***-TS =*** -0.032963653463016  ***G =*** -0.848390541533544  S 0.000000 0.000000 -0.014324  F 0.000000 1.831008 0.067415  F 0.000000 0.000000 1.640351  F 0.000000 -1.831008 0.067415 | ***E =*** -0.67623905  ***H =*** -0.665751592295563  ***-TS =*** -0.037156810775752  ***G =*** -0.702908403071315  S -0.002866 -0.140589 0.000000  Cl 0.048423 2.329399 0.000000  Cl 2.068241 -0.310752 0.000000  F -0.251224 -1.909511 0.000000 |
| **F_2_Se∙∙∙F^–^** | **Cl_2_Se∙∙∙F^–^** |
| ***E =*** -0.81925427  ***H =*** -0.808297226288133  ***-TS =*** -0.034761303728467  ***G =*** -0.843058530016600  Se 0.000000 0.000000 -0.040919  F 0.000000 1.944162 0.038547  F 0.000000 0.000000 1.753074  F 0.000000 -1.944162 0.038547 | ***E =*** -0.67243461  ***H =*** -0.662233719825299  ***-TS =*** -0.038885649325475  ***G =*** -0.701119369150773  Se -0.079949 -0.121396 0.000000  Cl 0.078475 2.389735 0.000000  Cl 2.120862 -0.447640 0.000000  F -0.471172 -2.007621 0.000000 |
| **F_2_S∙∙∙Cl^–^** | **Cl_2_S∙∙∙Cl^–^** |
| ***E =*** -0.75565811  ***H =*** -0.744558762355124  ***-TS =*** -0.035549952167987  ***G =*** -0.780108714523111  S -0.006575 0.134190 0.000000  F 0.350041 1.868159 0.000000  F 1.601466 -0.183039 0.000000  Cl -0.443820 -2.289822 0.000000 | ***E =*** -0.60493516  ***H =*** -0.594770279118590  ***-TS =*** -0.038367574140997  ***G =*** -0.633137853259587  S 0.000000 0.000000 -0.387287  Cl 0.000000 -2.369920 -0.649427  Cl 0.000000 0.000000 1.686142  Cl 0.000000 2.369920 -0.649427 |
| **F_2_Se∙∙∙Cl^–^** | **Cl_2_Se∙∙∙Cl^–^** |
| ***E =*** -0.74787680  ***H =*** -0.737213212516704  ***-TS =*** -0.037182619349818  ***G =*** -0.774395831866521  Se -0.007990 0.096805 0.000000  F 0.377994 1.968838 0.000000  F 1.748550 -0.229372 0.000000  Cl -0.472756 -2.360960 0.000000 | ***E =*** -0.60219719  ***H =*** -0.592255525230789  ***-TS =*** -0.040003089292676  ***G =*** -0.632258614523465  Se 0.000000 0.000000 -0.404638  Cl 0.000000 -2.446882 -0.694020  Cl 0.000000 0.000000 1.817623  Cl 0.000000 2.446882 -0.694020 |

**Table S14.** Cartesian coordinates, bonding energies, *H, TS,* and *G* (in a.u. at 298 K) for all stationary points computed at ZORA-BHANDH/QZ4P in the gas phase using ADF.

| **SF_2_** | **SCl_2_** |
| --- | --- |
| ***E =*** -0.81453102  ***H =*** -0.805301877809458  ***-TS =*** -0.029088903580469  ***G =*** -0.834390781389927  S 0.000000 0.000000 0.326381  F 0.000000 1.172061 1.355192  F 0.000000 -1.172061 1.355192 | ***E =*** -0.58835499  ***H =*** -0.580576907970876  ***-TS =*** -0.031757733301278  ***G =*** -0.612334641272154  S 0.000000 0.000000 0.249959  Cl 0.000000 1.551130 1.491369  Cl 0.000000 -1.551130 1.491369 |
| **SeF_2_** | **SeCl_2_** |
| ***E =*** -0.77636909  ***H =*** -0.767837531903627  ***-TS =*** -0.030643464080752  ***G =*** -0.798480995984379  Se 0.000000 0.000000 0.182582  F 0.000000 1.255838 1.324442  F 0.000000 -1.255838 1.324442 | ***E =*** -0.56153620  ***H =*** -0.554157343237090  ***-TS =*** -0.033288133170602  ***G =*** -0.587445476407692  Se 0.000000 0.000000 0.136330  Cl 0.000000 1.628675 1.493059  Cl 0.000000 -1.628675 1.493059 |
| **F_2_S∙∙∙F^–^** | **Cl_2_S∙∙∙F^–^** |
| ***E =*** -1.18713039  ***H =*** -1.175108078746544  ***-TS =*** -0.032259310684597  ***G =*** -1.207367389431140  S 0.000000 0.000000 -0.460772  F 0.000000 -1.754001 -0.330131  F 0.000000 0.000000 1.140122  F 0.000000 1.754001 -0.330131 | ***E =*** -0.96559588  ***H =*** -0.954751882283297  ***-TS =*** -0.036389918226012  ***G =*** -0.991141800509310  S -0.027571 -0.152055 0.000000  Cl 0.112135 2.270027 0.000000  Cl 1.984847 -0.275622 0.000000  F -0.173421 -1.845594 0.000000 |
| **F_2_Se∙∙∙F^–^** | **Cl_2_Se∙∙∙F^–^** |
| ***E =*** -1.16618233  ***H =*** -1.154778317230570  ***-TS =*** -0.033980184325441  ***G =*** -1.188758501556012  Se 0.000000 0.000000 -0.485972  F 0.000000 -1.867058 -0.323221  F 0.000000 0.000000 1.245920  F 0.000000 1.867058 -0.323221 | ***E =*** -0.95080502  ***H =*** -0.940364903785158  ***-TS =***  -0.037934368195503  ***G =*** -0.978299271980660  Se -0.020884 -0.094601 0.000000  Cl 0.126076 2.348188 0.000000  Cl 2.127705 -0.257257 0.000000  F -0.147485 -1.941364 0.000000 |
| **F_2_S∙∙∙Cl^–^** | **Cl_2_S∙∙∙Cl^–^** |
| ***E =*** -1.08118284  ***H =*** -1.069593802962102  ***-TS =*** -0.034889119162053  ***G =*** -1.104482922124154  S -0.021023 0.149632 0.000000  F 0.379533 1.789421 0.000000  F 1.531263 -0.185403 0.000000  Cl -0.388661 -2.224162 0.000000 | ***E =*** -0.85748630  ***H =*** -0.847119893169980  ***-TS =*** -0.037459526199657  ***G =*** -0.884579419369636  S 0.000000 0.000000 -0.449966  Cl 0.000000 -2.299096 -0.558220  Cl 0.000000 0.000000 1.561034  Cl 0.000000 2.299096 -0.558220 |
| **F_2_Se∙∙∙Cl^–^** | **Cl_2_Se∙∙∙Cl^–^** |
| ***E =*** -1.06157044  ***H =*** -1.050549336868798  ***-TS =*** -0.036401829630381  ***G =*** -1.086951166499179  Se -0.054229 0.127188 0.000000  F 0.418348 1.901237 0.000000  F 1.638985 -0.208142 0.000000  Cl -0.394684 -2.277404 0.000000 | ***E =*** -0.84629949  ***H =*** -0.836184719096211  ***-TS =*** -0.038946222974243  ***G =*** -0.875130942070454  Se 0.000000 0.000000 -0.464375  Cl 0.000000 -2.388859 -0.559751  Cl 0.000000 0.000000 1.685607  Cl 0.000000 2.388859 -0.559751 |

**Table S15.** Cartesian coordinates, bonding energies, *H, TS,* and *G* (in a.u. at 298 K) for all stationary points computed at ZORA-BLYP/QZ4P in the gas phase using ADF.

| **SF_2_** | **SCl_2_** |
| --- | --- |
| ***E =*** -0.37535631  ***H =*** -0.366867802687547  ***-TS =*** -0.029480664236479  ***G =*** -0.396348466924026  S 0.244713 0.192338 0.000000  F 0.173831 1.825011 0.000000  F 1.867552 -0.000156 0.000000 | ***E =*** -0.25797703  ***H =*** -0.250577829530876  ***-TS =*** -0.032910463558409  ***G =*** -0.283488293089285  S 0.176028 0.162238 0.000000  Cl -0.000033 2.223212 0.000000  Cl 2.209124 -0.204690 0.000000 |
| **SeF_2_** | **SeCl_2_** |
| ***E =*** -0.35844874  ***H =*** -0.350493642219296  ***-TS =*** -0.031097210646916  ***G =*** -0.381590852866212  Se 0.165618 0.077312 0.000000  F 0.091194 1.856634 0.000000  F 1.938440 -0.093457 0.000000 | ***E =*** -0.24623523  ***H =*** -0.239107460859860  ***-TS =*** -0.033834439547864  ***G =*** -0.272941900407724  Se 0.120253 0.063559 0.000000  Cl -0.057976 2.265177 0.000000  Cl 2.301559 -0.276254 0.000000 |
| **F_2_S∙∙∙F^–^** | **Cl_2_S∙∙∙F^–^** |
| ***E =*** -0.61139494  ***H =*** -0.600287049987374  ***-TS =*** -0.033439598863849  ***G =*** -0.633726648851223  S 0.000000 0.000000 -0.004682  F 0.000000 1.872757 0.035231  F 0.000000 0.000000 1.688493  F 0.000000 -1.872757 0.035231 | ***E =*** -0.49798402  ***H =*** -0.487685592933710  ***-TS =*** -0.037815032326078  ***G =*** -0.525500625259788  S 0.025915 -0.125618 0.000000  Cl -0.013248 2.353613 0.000000  Cl 2.141659 -0.332925 0.000000  F -0.321280 -1.938465 0.000000 |
| **F_2_Se∙∙∙F^–^** | **Cl_2_Se∙∙∙F^–^** |
| ***E =*** -0.60982157  ***H =*** -0.599122381686064  ***-TS =*** -0.035313510645768  ***G =*** -0.634435892331832  Se 0.000000 0.000000 -0.473148  F 0.000000 -1.984133 -0.462798  F 0.000000 0.000000 1.362937  F 0.000000 1.984133 -0.462798 | ***E =*** -0.49913392  ***H =*** -0.489080283565852  ***-TS =*** -0.040449181142465  ***G =*** -0.529529464708318  Se 0.081259 -0.080206 0.000000  Cl -0.144003 2.446462 0.000000  Cl 2.363556 -0.379666 0.000000  F -0.514142 -1.957493 0.000000 |
| **F_2_S∙∙∙Cl^–^** | **Cl_2_S∙∙∙Cl^–^** |
| ***E =*** -0.55802612  ***H =*** -0.547198327124024  ***-TS =*** -0.035982697205806  ***G =*** -0.583181024329830  S 0.010643 0.117602 0.000000  F 0.326929 1.917635 0.000000  F 1.660722 -0.189504 0.000000  Cl -0.479199 -2.298024 0.000000 | ***E =*** -0.44524815  ***H =*** -0.435234860428883  ***-TS =*** -0.039107794594125  ***G =*** -0.474342655023008  S 0.000000 0.000000 -0.326000  Cl 0.000000 -2.383257 -0.733206  Cl 0.000000 0.000000 1.797665  Cl 0.000000 2.383257 -0.733206 |
| **F_2_Se∙∙∙Cl^–^** | **Cl_2_Se∙∙∙Cl^–^** |
| ***E =*** -0.55601473  ***H =*** -0.545555032392124  ***-TS =*** -0.037803822163987  ***G =*** -0.583358854556111  Se -0.004162 0.117236 0.000000  F 0.267883 2.055627 0.000000  F 1.803750 -0.166113 0.000000  Cl -0.516874 -2.361372 0.000000 | ***E =*** -0.44750687  ***H =*** -0.437714475275520  ***-TS =*** -0.042081729424703  ***G =*** -0.479796204700223  Se 0.000000 0.000000 -0.093307  Cl 0.000000 -2.109892 -1.312603  Cl 0.000000 0.000000 2.343286  Cl 0.000000 2.109892 -1.312603 |

**Table S16.** Cartesian coordinates, bonding energies, *H, TS,* and *G* (in a.u. at 298 K) for all stationary points computed at ZORA-BLYP-D3(BJ)/QZ4P in the gas phase using ADF.

| **SF_2_** | **SCl_2_** |
| --- | --- |
| ***E =*** -0.37757290  ***H =*** -0.369082556755511  ***-TS =*** -0.029478972667953  ***G =*** -0.398561529423464  S 0.244536 0.192300 0.000000  F 0.174412 1.824825 0.000000  F 1.867269 0.000094 0.000000 | ***E =*** -0.26521668  ***H =*** -0.257807222234983  ***-TS =*** -0.032237877778122  ***G =*** -0.290045100013106  S 0.173222 0.160190 0.000000  Cl 0.004710 2.219032 0.000000  Cl 2.206946 -0.198637 0.000000 |
| **SeF_2_** | **SeCl_2_** |
| ***E =*** -0.36087683  ***H =*** -0.352920042225678  ***-TS =*** -0.031094847612001  ***G =*** -0.384014889837678  Se 0.165516 0.077144 0.000000  F 0.091782 1.856297 0.000000  F 1.938278 -0.092414 0.000000 | ***E =*** -0.25380379  ***H =*** -0.246670300073341  ***-TS =*** -0.033813977671181  ***G =*** -0.280484277744522  Se 0.117970 0.060999 0.000000  Cl -0.050273 2.261129 0.000000  Cl 2.299074 -0.266357 0.000000 |
| **F_2_S∙∙∙F^–^** | **Cl_2_S∙∙∙F^–^** |
| ***E =*** -0.61493503  ***H =*** -0.603823883010586  ***-TS =*** -0.033431576176247  ***G =*** -0.637255459186833  S 0.000000 0.000000 -0.005253  F 0.000000 1.872101 0.036071  F 0.000000 0.000000 1.687773  F 0.000000 -1.872101 0.036071 | ***E =*** -0.50671788  ***H =*** -0.496411892558714  ***-TS =*** -0.037740780261392  ***G =*** -0.534152672820106  S 0.020403 -0.123559 0.000000  Cl -0.001279 2.343899 0.000000  Cl 2.135374 -0.324821 0.000000  F -0.322466 -1.938964 0.000000 |
| **F_2_Se∙∙∙F^–^** | **Cl_2_Se∙∙∙F^–^** |
| ***E =*** -0.61364687  ***H =*** -0.602931605945884  ***-TS =*** -0.035283986095544  ***G =*** -0.638215592041428  Se 0.000000 0.000000 -0.467560  F 0.000000 -1.982700 -0.449780  F 0.000000 0.000000 1.367120  F 0.000000 1.982700 -0.449780 | ***E =*** -0.50803026  ***H =*** -0.497957535972183  ***-TS =*** -0.039882549154044  ***G =*** -0.537840085126227  Se 0.051606 -0.084600 0.000000  Cl -0.074481 2.440353 0.000000  Cl 2.322459 -0.344243 0.000000  F -0.441732 -1.992971 0.000000 |
| **F_2_S∙∙∙Cl^–^** | **Cl_2_S∙∙∙Cl^–^** |
| ***E =*** -0.56366528  ***H =*** -0.552827548021767  ***-TS =*** -0.035939457217106  ***G =*** -0.588767005238873  S 0.006614 0.116614 0.000000  F 0.331882 1.914243 0.000000  F 1.655814 -0.191785 0.000000  Cl -0.475540 -2.294038 0.000000 | ***E =*** -0.45677490  ***H =*** -0.446741089026122  ***-TS =*** -0.038907361597159  ***G =*** -0.485648450623281  S 0.000000 0.000000 -0.349776  Cl 0.000000 -2.384052 -0.707125  Cl 0.000000 0.000000 1.767241  Cl 0.000000 2.384052 -0.707125 |
| **F_2_Se∙∙∙Cl^–^** | **Cl_2_Se∙∙∙Cl^–^** |
| ***E =*** -0.56203184  ***H =*** -0.551565196383884  ***-TS =*** -0.037763628588778  ***G =*** -0.589328824972662  Se -0.007349 0.117703 0.000000  F 0.275106 2.053365 0.000000  F 1.798801 -0.171881 0.000000  Cl -0.510826 -2.358078 0.000000 | ***E =*** -0.45824389  ***H =*** -0.449359101349574  ***-TS =*** -0.038660408023489  ***G =*** -0.488019509373063  Se 0.000000 0.000000 -0.278856  Cl 0.000000 -2.392768 -0.943091  Cl 0.000000 0.000000 2.028309  Cl 0.000000 2.392768 -0.943091 |

**Table S17.** Cartesian coordinates, bonding energies, *H, TS,* and *G* (in a.u. at 298 K) for all stationary points computed at ZORA-BP86/QZ4P in the gas phase using ADF.

| **SF_2_** | **SCl_2_** |
| --- | --- |
| ***E =***  -0.39132620  ***H =*** -0.382717210547094  ***-TS =*** -0.029410250155590  ***G =*** -0.412127460702683  S 0.248527 0.195408 0.000000  F 0.177266 1.814079 0.000000  F 1.857699 0.005610 0.000000 | ***E =*** -0.28105185  ***H =*** -0.273565289008674  ***-TS =*** -0.032116813313952  ***G =*** -0.305682102322625  S 0.181711 0.168187 0.000000  Cl 0.012971 2.202184 0.000000  Cl 2.190923 -0.189101 0.000000 |
| **SeF_2_** | **SeCl_2_** |
| ***E =*** -0.37201622  ***H =*** -0.363974535249983  ***-TS =*** -0.031014656692074  ***G =*** -0.394989191942056  Se 0.168149 0.079685 0.000000  F 0.094557 1.842313 0.000000  F 1.924429 -0.089119 0.000000 | ***E =*** -0.26769882  ***H =*** -0.260515831560801  ***-TS =*** -0.033685205219599  ***G =*** -0.294201036780400  Se 0.124567 0.067518 0.000000  Cl -0.044927 2.240125 0.000000  Cl 2.278650 -0.260249 0.000000 |
| **F_2_S∙∙∙F^–^** | **Cl_2_S∙∙∙F^–^** |
| ***E =*** -0.63419296  ***H =*** -0.622929903115086  ***-TS =*** -0.033169337781319  ***G =*** -0.656099240896405  S 0.000000 0.000000 -0.010948  F 0.000000 1.844495 0.051679  F 0.000000 0.000000 1.666142  F 0.000000 -1.844495 0.051679 | ***E =*** -0.52759180  ***H =*** -0.517184475130055  ***-TS =*** -0.037347210181979  ***G =*** -0.554531685312035  S 0.009256 -0.118289 0.000000  Cl 0.031436 2.310769 0.000000  Cl 2.091816 -0.307988 0.000000  F -0.283748 -1.917837 0.000000 |
| **F_2_Se∙∙∙F^–^** | **Cl_2_Se∙∙∙F^–^** |
| ***E =*** -0.63001681  ***H =*** -0.619201451277690  ***-TS =*** -0.035010391445824  ***G =*** -0.654211842723513  Se 0.000000 0.000000 -0.481517  F 0.000000 -1.956854 -0.431008  F 0.000000 0.000000 1.334563  F 0.000000 1.956854 -0.431008 | ***E =*** -0.52674529  ***H =*** -0.516607028663383  ***-TS =*** -0.039345513308083  ***G =*** -0.555952541971467  Se 0.034738 -0.082024 0.000000  Cl -0.022053 2.412031 0.000000  Cl 2.268292 -0.322866 0.000000  F -0.367571 -1.991019 0.000000 |
| **F_2_S∙∙∙Cl^–^** | **Cl_2_S∙∙∙Cl^–^** |
| ***E =*** -0.58418797  ***H =*** -0.573226689623070  ***-TS =*** -0.035640298845443  ***G =*** -0.608866988468513  S -0.010261 0.110332 0.000000  F 0.336847 1.880052 0.000000  F 1.624268 -0.194500 0.000000  Cl -0.445670 -2.268243 0.000000 | ***E =*** -0.47786968  ***H =*** -0.467767242294662  ***-TS =*** -0.038527396246618  ***G =*** -0.506294638541280  S 0.000000 0.000000 -0.367433  Cl 0.000000 -2.356845 -0.673825  Cl 0.000000 0.000000 1.716785  Cl 0.000000 2.356845 -0.673825 |
| **F_2_Se∙∙∙Cl^–^** | **Cl_2_Se∙∙∙Cl^–^** |
| ***E =*** -0.58008718  ***H =*** -0.569529918629254  ***-TS =*** -0.037434764772720  ***G =*** -0.606964683401974  Se -0.019716 0.115021 0.000000  F 0.310102 2.022771 0.000000  F 1.763370 -0.183147 0.000000  Cl -0.482328 -2.329205 0.000000 | ***E =*** -0.47777541  ***H =*** -0.467889659349917  ***-TS =*** -0.040732003845060  ***G =*** -0.508621663194977  Se 0.000000 0.000000 -0.341459  Cl 0.000000 -2.411206 -0.813243  Cl 0.000000 0.000000 1.911683  Cl 0.000000 2.411206 -0.813243 |

**Table S18.** Cartesian coordinates, bonding energies, *H, TS,* and *G* (in a.u. at 298 K) for all stationary points computed at ZORA-M06/QZ4P in the gas phase using ADF.

| **SF_2_** | **SCl_2_** |
| --- | --- |
| ***E =*** -0.58317540  ***H =*** -0.574240358752756  ***-TS =*** -0.029216884179750  ***G =*** -0.603457242932505  S 0.253774 0.198863 0.000000  F 0.195813 1.783485 0.000000  F 1.830322 0.030390 0.000000 | ***E =*** -0.41876385  ***H =*** -0.411137431558732  ***-TS =*** -0.031924127693741  ***G =*** -0.443061559252473  S 0.181126 0.166161 0.000000  Cl 0.034470 2.177390 0.000000  Cl 2.169959 -0.162455 0.000000 |
| **SeF_2_** | **SeCl_2_** |
| ***E =*** -0.56648637  ***H =*** -0.558221614722090  ***-TS =*** -0.030826470619182  ***G =*** -0.589048085341273  Se 0.169527 0.080201 0.000000  F 0.119332 1.809577 0.000000  F 1.893857 -0.058553 0.000000 | ***E =*** -0.40649054  ***H =*** -0.399230446720274  ***-TS =*** -0.033484998460658  ***G =*** -0.432715445180932  Se 0.120609 0.062402 0.000000  Cl -0.013631 2.212761 0.000000  Cl 2.256400 -0.221195 0.000000 |
| **F_2_S∙∙∙F^–^** | **Cl_2_S∙∙∙F^–^** |
| ***E =*** -0.88105615  ***H =*** -0.869523018527910  ***-TS =*** -0.032807763799113  ***G =*** -0.902330782327023  S 0.000000 0.000000 -0.454718  F 0.000000 -1.810328 -0.360719  F 0.000000 0.000000 1.176226  F 0.000000 1.810328 -0.360719 | ***E =*** -0.72132055  ***H =*** -0.710759146070105  ***-TS =*** -0.037053681005026  ***G =*** -0.747812827075131  S -0.012196 -0.152726 0.000000  Cl 0.081504 2.322126 0.000000  Cl 2.032902 -0.304473 0.000000  F -0.231367 -1.887256 0.000000 |
| **F_2_Se∙∙∙F^–^** | **Cl_2_Se∙∙∙F^–^** |
| ***E =*** -0.88390297  ***H =*** -0.872903803300214  ***-TS =*** -0.034626856731236  ***G =*** -0.907530660031449  Se 0.000000 0.000000 -0.488185  F 0.000000 1.928585 -0.399480  F 0.000000 0.000000 1.284980  F 0.000000 -1.928585 -0.399480 | ***E =*** -0.72576893  ***H =*** -0.715528296319326  ***-TS =*** -0.038685971511491  ***G =*** -0.754214267830817  Se 0.001729 -0.093928 0.000000  Cl 0.070099 2.409373 0.000000  Cl 2.192185 -0.299727 0.000000  F -0.258254 -1.978647 0.000000 |
| **F_2_S∙∙∙Cl^–^** | **Cl_2_S∙∙∙Cl^–^** |
| ***E =*** -0.80536408  ***H =*** -0.794145511130727  ***-TS =*** -0.035459615729927  ***G =*** -0.829605126860654  S 0.154985 -0.438348 0.000000  F 1.888345 -0.512288 0.000000  F 0.244045 1.176172 0.000000  Cl -2.287375 -0.225538 0.000000 | ***E =*** -0.64434833  ***H =*** -0.634135255272223  ***-TS =*** -0.038092984728241  ***G =*** -0.672228240000465  S 0.000000 0.000000 -0.421408  Cl 0.000000 -2.332398 -0.602411  Cl 0.000000 0.000000 1.626215  Cl 0.000000 2.332398 -0.602411 |
| **F_2_Se∙∙∙Cl^–^** | **Cl_2_Se∙∙∙Cl^–^** |
| ***E =*** -0.80699683  ***H =*** -0.796285240850989  ***-TS =*** -0.037091266542003  ***G =*** -0.833376507392992  Se -0.028702 0.127766 0.000000  F 0.354562 1.975965 0.000000  F 1.707769 -0.187626 0.000000  Cl -0.455738 -2.330469 0.000000 | ***E =*** -0.64873503  ***H =*** -0.638758356654638  ***-TS =*** -0.039744447818169  ***G =*** -0.678502804472807  Se 0.000000 0.000000 -0.436147  Cl 0.000000 -2.427787 -0.662885  Cl 0.000000 0.000000 1.761645  Cl 0.000000 2.427787 -0.662885 |

**Table S19.** Cartesian coordinates, bonding energies, *H, TS,* and *G* (in a.u. at 298 K) for all stationary points computed at ZORA-M06-HF/QZ4P in the gas phase using ADF.

| **SF_2_** | **SCl_2_** |
| --- | --- |
| ***E =*** -1.07773090  ***H =*** -1.068596677512026  ***-TS =*** -0.029176198629097  ***G =*** -1.097772876141123  S 0.249326 0.194489 0.000000  F 0.202712 1.782944 0.000000  F 1.830908 0.038291 0.000000 | ***E =*** -0.77309290  ***H =*** -0.765463944590128  ***-TS =*** -0.031895227815797  ***G =*** -0.797359172405925  S 0.164164 0.149022 0.000000  Cl 0.056178 2.165449 0.000000  Cl 2.163759 -0.134844 0.000000 |
| **SeF_2_** | **SeCl_2_** |
| ***E =*** -1.02928265  ***H =*** -1.020788334635598  ***-TS =*** -0.030684165986310  ***G =*** -1.051472500621908  Se 0.168229 0.079155 0.000000  F 0.131271 1.802158 0.000000  F 1.887379 -0.046735 0.000000 | ***E =*** -0.74132886  ***H =*** -0.734022432728190  ***-TS =*** -0.033415891608282  ***G =*** -0.767438324336472  Se 0.114821 0.055483 0.000000  Cl 0.005761 2.203134 0.000000  Cl 2.250237 -0.195756 0.000000 |
| **F_2_S∙∙∙F^–^** | **Cl_2_S∙∙∙F^–^** |
| ***E =*** -1.56095245  ***H =*** -1.548958412332494  ***-TS =*** -0.032367284216927  ***G =*** -1.581325696549421  S 0.000000 0.000000 -0.023238  F 0.000000 1.781516 0.091432  F 0.000000 0.000000 1.607318  F 0.000000 -1.781516 0.091432 | ***E =*** -1.26079088  ***H =*** -1.249956121567594  ***-TS =*** -0.036358141842250  ***G =*** -1.286314263409844  S -0.105002 -0.141361 0.000000  Cl 0.143149 2.290029 0.000000  Cl 1.933433 -0.350678 0.000000  F -0.306592 -1.858996 0.000000 |
| **F_2_Se∙∙∙F^–^** | **Cl_2_Se∙∙∙F^–^** |
| ***E =*** -1.52604530  ***H =*** -1.514549142144410  ***-TS =*** -0.033909312298200  ***G =*** -1.548458454442611  Se 0.000000 0.000000 -0.505589  F 0.000000 -1.885172 -0.338376  F 0.000000 0.000000 1.250556  F 0.000000 1.885172 -0.338376 | ***E =*** -1.23448560  ***H =*** -1.224006539583429  ***-TS =*** -0.037832467282292  ***G =*** -1.261839006865721  Se -0.143944 -0.075174 0.000000  Cl 0.170737 2.374822 0.000000  Cl 2.015109 -0.342360 0.000000  F -0.332618 -1.935016 0.000000 |
| **F_2_S∙∙∙Cl^–^** | **Cl_2_S∙∙∙Cl^–^** |
| ***E =*** -1.41635909  ***H =*** -1.404776932857814  ***-TS =*** -0.034935299691356  ***G =*** -1.439712232549169  S -0.020518 0.153384 0.000000  F 0.302865 1.838180 0.000000  F 1.574817 -0.117739 0.000000  Cl -0.314583 -2.261233 0.000000 | ***E =*** -1.11422934  ***H =*** -1.103868782765284  ***-TS =*** -0.037383164054842  ***G =*** -1.141251946820125  S 0.000000 0.000000 -0.470808  Cl 0.000000 -2.336895 -0.555741  Cl 0.000000 0.000000 1.575132  Cl 0.000000 2.336895 -0.555741 |
| **F_2_Se∙∙∙Cl^–^** | **Cl_2_Se∙∙∙Cl^–^** |
| ***E =*** -1.38324704  ***H =*** -1.372164189179677  ***-TS =*** -0.036390705224109  ***G =*** -1.408554894403786  Se -0.056455 0.132745 0.000000  F 0.427609 1.922033 0.000000  F 1.658860 -0.212799 0.000000  Cl -0.405427 -2.298870 0.000000 | ***E =*** -1.09356784  ***H =*** -1.083450550234903  ***-TS =*** -0.038827746293287  ***G =*** -1.122278296528190  Se 0.000000 0.000000 -0.492484  Cl 0.000000 -2.414884 -0.525251  Cl 0.000000 0.000000 1.680847  Cl 0.000000 2.414884 -0.525251 |

**Table S20.** Cartesian coordinates, bonding energies, *H, TS,* and *G* (in a.u. at 298 K) for all stationary points computed at ZORA-M06-L/QZ4P in the gas phase using ADF.

| **SF_2_** | **SCl_2_** |
| --- | --- |
| ***E =*** -0.42801302  ***H =*** -0.419223454262439  ***-TS =*** -0.029285326475516  ***G =*** -0.448508780737955  S 0.251329 0.197999 0.000000  F 0.194039 1.791516 0.000000  F 1.836211 0.023813 0.000000 | ***E =*** -0.31069558  ***H =*** -0.303056098612944  ***-TS =*** -0.032541638901486  ***G =*** -0.335597737514429  S 0.191585 0.176074 0.000000  Cl 0.029881 2.174917 0.000000  Cl 2.164985 -0.169045 0.000000 |
| **SeF_2_** | **SeCl_2_** |
| ***E =*** -0.41405672  ***H =*** -0.405906318245825  ***-TS =*** -0.030929676186973  ***G =*** -0.436835994432798  Se 0.168353 0.079067 0.000000  F 0.111021 1.823125 0.000000  F 1.907109 -0.067331 0.000000 | ***E =*** -0.29726232  ***H =*** -0.290032286493731  ***-TS =*** -0.033514449676856  ***G =*** -0.323546736170587  Se 0.124911 0.067082 0.000000  Cl -0.022941 2.214193 0.000000  Cl 2.255880 -0.233323 0.000000 |
| **F_2_S∙∙∙F^–^** | **Cl_2_S∙∙∙F^–^** |
| ***E =*** -0.66948402  ***H =*** -0.658041659842259  ***-TS =*** -0.032909834636190  ***G =*** -0.690951494478449  S 0.000000 0.000000 -0.465230  F 0.000000 -1.820710 -0.356860  F 0.000000 0.000000 1.178950  F 0.000000 1.820710 -0.356860 | ***E =*** -0.55658838  ***H =*** -0.546065345645256  ***-TS =*** -0.037215170669685  ***G =*** -0.583280516314941  S -0.000450 -0.167520 0.000000  Cl 0.073287 2.354370 0.000000  Cl 2.029116 -0.306978 0.000000  F -0.229042 -1.917098 0.000000 |
| **F_2_Se∙∙∙F^–^** | **Cl_2_Se∙∙∙F^–^** |
| ***E =*** -0.67356798  ***H =*** -0.662656897588206  ***-TS =*** -0.034773523922519  ***G =*** -0.697430421510725  Se 0.000000 0.000000 0.002756  F 0.000000 1.941621 0.112234  F 0.000000 0.000000 1.793520  F 0.000000 -1.941621 0.112234 | ***E =*** -0.56109661  ***H =*** -0.550897930163459  ***-TS =*** -0.038936719601079  ***G =*** -0.589834649764538  Se 0.009850 -0.100841 0.000000  Cl 0.047946 2.440355 0.000000  Cl 2.204615 -0.306927 0.000000  F -0.274517 -1.993340 0.000000 |
| **F_2_S∙∙∙Cl^–^** | **Cl_2_S∙∙∙Cl^–^** |
| ***E =*** -0.61712618  ***H =*** -0.606020673391157  ***-TS =*** -0.035657501155985  ***G =*** -0.641678174547142  S -0.017337 0.150882 0.000000  F 0.350373 1.857678 0.000000  F 1.577170 -0.171457 0.000000  Cl -0.420960 -2.305682 0.000000 | ***E =*** -0.50181158  ***H =*** -0.491676555712136  ***-TS =*** -0.038746658382597  ***G =*** -0.530423214094734  S 0.000000 0.000000 -0.399306  Cl 0.000000 -2.362016 -0.616158  Cl 0.000000 0.000000 1.630592  Cl 0.000000 2.362016 -0.616158 |
| **F_2_Se∙∙∙Cl^–^** | **Cl_2_Se∙∙∙Cl^–^** |
| ***E =*** -0.62048364  ***H =*** -0.609850824677313  ***-TS =*** -0.037278871871803  ***G =*** -0.647129696549115  Se -0.031463 0.132862 0.000000  F 0.360759 1.993024 0.000000  F 1.720528 -0.187838 0.000000  Cl -0.459728 -2.351268 0.000000 | ***E =*** -0.50653955  ***H =*** -0.496612759579723  ***-TS =*** -0.040105892748117  ***G =*** -0.536718652327840  Se 0.000000 0.000000 -0.404772  Cl 0.000000 -2.450284 -0.682249  Cl 0.000000 0.000000 1.794389  Cl 0.000000 2.450284 -0.682249 |

**Table S21.** Cartesian coordinates, bonding energies, *H, TS,* and *G* (in a.u. at 298 K) for all stationary points computed at ZORA-M06-2X/QZ4P in the gas phase using ADF.

| **SF_2_** | **SCl_2_** |
| --- | --- |
| ***E =*** -0.77816601  ***H =*** -0.769127715171731  ***-TS =***  -0.029197536207457  ***G =*** -0.798325251379187  S 0.251096 0.196622 0.000000  F 0.200526 1.783127 0.000000  F 1.830116 0.034519 0.000000 | ***E =*** -0.55969109  ***H =*** -0.552042493980340  ***-TS =*** -0.031890788959357  ***G =*** -0.583933282939697  S 0.175535 0.161230 0.000000  Cl 0.043445 2.171259 0.000000  Cl 2.166096 -0.151815 0.000000 |
| **SeF_2_** | **SeCl_2_** |
| ***E =*** -0.74742750  ***H =*** -0.739057714375466  ***-TS =***  -0.030766536906481  ***G =*** -0.769824251281947  Se 0.168901 0.079083 0.000000  F 0.123237 1.806742 0.000000  F 1.892588 -0.051018 0.000000 | ***E =*** -0.53707186  ***H =*** -0.529775802662624  ***-TS =*** -0.033428150627500  ***G =*** -0.563203953290124  Se 0.119375 0.060602 0.000000  Cl -0.005962 2.206872 0.000000  Cl 2.251553 -0.211192 0.000000 |
| **F_2_S∙∙∙F^–^** | **Cl_2_S∙∙∙F^–^** |
| ***E =*** -1.14703847  ***H =*** -1.135247741459142  ***-TS =*** -0.032525841361272  ***G =*** -1.167773582820415  S 0.000000 0.000000 -0.025735  F 0.000000 1.790928 0.094557  F 0.000000 0.000000 1.605294  F 0.000000 -1.790928 0.094557 | ***E =*** -0.93302867  ***H =*** -0.922314302646153  ***-TS =*** -0.036650656691159  ***G =*** -0.958964959337312  S -0.031140 -0.150407 0.000000  Cl 0.107627 2.307071 0.000000  Cl 2.009874 -0.296785 0.000000  F -0.205179 -1.877600 0.000000 |
| **F_2_Se∙∙∙F^–^** | **Cl_2_Se∙∙∙F^–^** |
| ***E =*** -1.13266165  ***H =*** -1.121412959999046  ***-TS =***  -0.034213399254894  ***G =*** -1.155626359253940  Se 0.000000 0.000000 -0.005475  F 0.000000 1.903818 0.146671  F 0.000000 0.000000 1.759269  F 0.000000 -1.903818 0.146671 | ***E =*** -0.92250172  ***H =*** -0.912142748815562  ***-TS =*** -0.038210513796404  ***G =*** -0.950353262611966  Se -0.026816 -0.095392 0.000000  Cl 0.137694 2.393666 0.000000  Cl 2.151279 -0.283620 0.000000  F -0.187307 -1.973225 0.000000 |
| **F_2_S∙∙∙Cl^–^** | **Cl_2_S∙∙∙Cl^–^** |
| ***E =*** -1.04548645  ***H =***  -1.034073056308179  ***-TS =***  -0.035141192128983  ***G =*** -1.069214248437161  S -0.027817 0.142554 0.000000  F 0.361651 1.820167 0.000000  F 1.555451 -0.186082 0.000000  Cl -0.405706 -2.269742 0.000000 | ***E =*** -0.82910658  ***H =*** -0.818821615666766  ***-TS =*** -0.037710810146058  ***G =*** -0.856532425812825  S 0.000000 0.000000 -0.446132  Cl 0.000000 -2.335094 -0.577264  Cl 0.000000 0.000000 1.595618  Cl 0.000000 2.335094 -0.577264 |
| **F_2_Se∙∙∙Cl^–^** | **Cl_2_Se∙∙∙Cl^–^** |
| ***E =*** -1.03086840  ***H =*** -1.019970859456504  ***-TS =***  -0.036715993753954  ***G =***  -1.056686853210458  Se -0.050170 0.134519 0.000000  F 0.412535 1.944038 0.000000  F 1.673768 -0.206313 0.000000  Cl -0.419700 -2.318405 0.000000 | ***E =*** -0.82082048  ***H =*** -0.810766020398492  ***-TS =*** -0.039222065097230  ***G =*** -0.849988085495722  Se 0.000000 0.000000 -0.467336  Cl 0.000000 -2.426002 -0.571815  Cl 0.000000 0.000000 1.716505  Cl 0.000000 2.426002 -0.571815 |

**Table S22.** Cartesian coordinates, bonding energies, *H, TS,* and *G* (in a.u. at 298 K) for all stationary points computed at ZORA-PBE/QZ4P in the gas phase using ADF.

| **SF_2_** | **SCl_2_** |
| --- | --- |
| ***E =*** -0.39487300  ***H =*** -0.386249569123912  ***-TS =*** -0.029403767642794  ***G =*** -0.415653336766706  S 0.248063 0.194888 0.000000  F 0.178931 1.812911 0.000000  F 1.856815 0.007653 0.000000 | ***E =*** -0.28845627  ***H =*** -0.280944595474955  ***-TS =*** -0.032079477795825  ***G =*** -0.313024073270779  S 0.183577 0.169609 0.000000  Cl 0.015836 2.197159 0.000000  Cl 2.186352 -0.185377 0.000000 |
| **SeF_2_** | **SeCl_2_** |
| ***E =*** -0.37576582  ***H =*** -0.367719441949402  ***-TS =*** -0.031012305908682  ***G =*** -0.398731747858083  Se 0.167736 0.079178 0.000000  F 0.096690 1.841603 0.000000  F 1.924035 -0.086279 0.000000 | ***E =*** -0.27472322  ***H =*** -0.267527522825922  ***-TS =*** -0.033653720060688  ***G =*** -0.301181242886611  Se 0.124709 0.067514 0.000000  Cl -0.040775 2.235011 0.000000  Cl 2.274175 -0.255127 0.000000 |
| **F_2_S∙∙∙F^–^** | **Cl_2_S∙∙∙F^–^** |
| ***E =*** -0.63561031  ***H =*** -0.624319441375037  ***-TS =*** -0.033128495581564  ***G =*** -0.657447936956600  S 0.000000 0.000000 -0.453100  F 0.000000 -1.841570 -0.384230  F 0.000000 0.000000 1.221560  F 0.000000 1.841570 -0.384230 | ***E =*** -0.53164379  ***H =*** -0.521214487341472  ***-TS =*** -0.037232959010144  ***G =*** -0.558447446351616  S 0.003342 -0.114227 0.000000  Cl 0.044592 2.299976 0.000000  Cl 2.078008 -0.301569 0.000000  F -0.272598 -1.916622 0.000000 |
| **F_2_Se∙∙∙F^–^** | **Cl_2_Se∙∙∙F^–^** |
| ***E =*** -0.63161111  ***H =*** -0.620783098082067  ***-TS =*** -0.034993895583614  ***G =*** -0.655776993665681  Se 0.000000 0.000000 -0.482110  F 0.000000 -1.956970 -0.425420  F 0.000000 0.000000 1.332950  F 0.000000 1.956970 -0.425420 | ***E =*** -0.53062245  ***H =*** -0.520469895344406  ***-TS =*** -0.039189439337891  ***G =*** -0.559659334682296  Se 0.026007 -0.080869 0.000000  Cl 0.001424 2.403105 0.000000  Cl 2.250843 -0.313126 0.000000  F -0.341941 -1.997379 0.000000 |
| **F_2_S∙∙∙Cl^–^** | **Cl_2_S∙∙∙Cl^–^** |
| ***E =*** -0.58654937  ***H =*** -0.575568817964437  ***-TS =*** -0.035558093967666  ***G =*** -0.611126911932103  S -0.020423 0.106017 0.000000  F 0.336538 1.873222 0.000000  F 1.614411 -0.196489 0.000000  Cl -0.430855 -2.259507 0.000000 | ***E =*** -0.48295666  ***H =*** -0.472832326338703  ***-TS =*** -0.038374889408333  ***G =*** -0.511207215747036  S 0.000000 0.000000 -0.381491  Cl 0.000000 -2.347903 -0.656789  Cl 0.000000 0.000000 1.695566  Cl 0.000000 2.347903 -0.656789 |
| **F_2_Se∙∙∙Cl^–^** | **Cl_2_Se∙∙∙Cl^–^** |
| ***E =*** -0.58260624  ***H =*** -0.572040990881811  ***-TS =*** -0.037365288170934  ***G =*** -0.609406279052745  Se -0.025723 0.113403 0.000000  F 0.322961 2.017923 0.000000  F 1.757076 -0.190225 0.000000  Cl -0.472166 -2.319029 0.000000 | ***E =*** -0.48253064  ***H =*** -0.472630323164822  ***-TS =*** -0.040452600910262  ***G =*** -0.513082924075084  Se 0.000000 0.000000 -0.363911  Cl 0.000000 -2.418105 -0.770315  Cl 0.000000 0.000000 1.877137  Cl 0.000000 2.418105 -0.770315 |

**Table S23.** Cartesian coordinates, bonding energies, *H, TS,* and *G* (in a.u. at 298 K) for all stationary points computed at ZORA-SSB-D/QZ4P in the gas phase using ADF.

| **SF_2_** | **SCl_2_** |
| --- | --- |
| ***E =*** -0.40233090  ***H =*** -0.393662179252451  ***-TS =*** -0.029393653980170  ***G =*** -0.423055833232621  S 0.257848 0.204333 0.000000  F 0.163727 1.813447 0.000000  F 1.855552 -0.008779 0.000000 | ***E =*** -0.30951477  ***H =*** -0.301879761437627  ***-TS =*** -0.031894453312315  ***G =*** -0.333774214749942  S 0.200163 0.185080 0.000000  Cl 0.022052 2.176154 0.000000  Cl 2.164972 -0.178517 0.000000 |
| **SeF_2_** | **SeCl_2_** |
| ***E =*** -0.38424104  ***H =*** -0.376209908042637  ***-TS =*** -0.031018362025553  ***G =*** -0.407228270068190  Se 0.174147 0.087423 0.000000  F 0.073754 1.842940 0.000000  F 1.920003 -0.122297 0.000000 | ***E =*** -0.29332689  ***H =*** -0.286076520507690  ***-TS =*** -0.033485248319066  ***G =*** -0.319561768826756  Se 0.136012 0.078629 0.000000  Cl -0.044521 2.214216 0.000000  Cl 2.252088 -0.259734 0.000000 |
| **F_2_S∙∙∙F^–^** | **Cl_2_S∙∙∙F^–^** |
| ***E =*** -0.64477971  ***H =*** -0.633390909325526  ***-TS =*** -0.033152718003792  ***G =*** -0.666543627329317  S 0.000000 0.000000 0.003989  F 0.000000 1.836123 0.037701  F 0.000000 0.000000 1.668959  F 0.000000 -1.836123 0.037701 | ***E =*** -0.55034158  ***H =*** -0.539841426111028  ***-TS =*** -0.037009244287091  ***G =*** -0.576850670398118  S 0.012555 -0.108424 0.000000  Cl 0.069162 2.273138 0.000000  Cl 2.042402 -0.286078 0.000000  F -0.267786 -1.905503 0.000000 |
| **F_2_Se∙∙∙F^–^** | **Cl_2_Se∙∙∙F^–^** |
| ***E =*** -0.64253276  ***H =*** -0.631656388702690  ***-TS =*** -0.035119502658675  ***G =*** -0.666775891361365  Se 0.000000 0.000000 -0.447893  F 0.000000 -1.960140 -0.455913  F 0.000000 0.000000 1.359718  F 0.000000 1.960140 -0.455913 | ***E =*** -0.54921765  ***H =*** -0.539031033205054  ***-TS =*** -0.038869481628371  ***G =*** -0.577900514833425  Se 0.027356 -0.083115 0.000000  Cl 0.029589 2.385948 0.000000  Cl 2.213360 -0.289957 0.000000  F -0.330466 -1.998994 0.000000 |
| **F_2_S∙∙∙Cl^–^** | **Cl_2_S∙∙∙Cl^–^** |
| ***E =*** -0.59933656  ***H =*** -0.588334141685269  ***-TS =*** -0.035580585836598  ***G =*** -0.623914727521867  S -0.109762 -0.411309 0.000000  F -1.898590 -0.574191 0.000000  F -0.242494 1.236938 0.000000  Cl 2.248347 -0.250709 0.000000 | ***E =*** -0.50519774  ***H =*** -0.494946649043992  ***-TS =*** -0.038071272345413  ***G =*** -0.533017921389405  S 0.000000 0.000000 -0.380807  Cl 0.000000 -2.312485 -0.636016  Cl 0.000000 0.000000 1.653395  Cl 0.000000 2.312485 -0.636016 |
| **F_2_Se∙∙∙Cl^–^** | **Cl_2_Se∙∙∙Cl^–^** |
| ***E =*** -0.59659952  ***H =*** -0.586047158694791  ***-TS =*** -0.037523080457112  ***G =*** -0.623570239151903  Se 0.003229 0.102161 0.000000  F 0.237266 2.024529 0.000000  F 1.783208 -0.181786 0.000000  Cl -0.505971 -2.301510 0.000000 | ***E =*** -0.50382616  ***H =*** -0.493854383159471  ***-TS =*** -0.040045753610955  ***G =*** -0.533900136770426  Se 0.000000 0.000000 -0.372300  Cl 0.000000 -2.404808 -0.734263  Cl 0.000000 0.000000 1.824207  Cl 0.000000 2.404808 -0.734263 |

**Table S24.** Cartesian coordinates, bonding energies, *H, TS,* and *G* (in a.u. at 298 K) for all stationary points computed at ZORA-SSB-D3(BJ)/QZ4P in the gas phase using ADF.

| **SF_2_** | **SCl_2_** |
| --- | --- |
| ***E =*** -0.40301968  ***H =*** -0.394342200047010  ***-TS =*** -0.029390055913329  ***G =*** -0.423732255960338  S 0.256961 0.203287 0.000000  F 0.166329 1.811758 0.000000  F 1.854442 -0.005328 0.000000 | ***E =*** -0.31204067  ***H =*** -0.304399128689208  ***-TS =*** -0.031888510165210  ***G =*** -0.336287638854418  S 0.197655 0.182479 0.000000  Cl 0.026976 2.172801 0.000000  Cl 2.162341 -0.172785 0.000000 |
| **SeF_2_** | **SeCl_2_** |
| ***E =*** -0.38487046  ***H =*** -0.376836031723442  ***-TS =*** -0.031031499035735  ***G =*** -0.407867530759177  Se 0.173634 0.086977 0.000000  F 0.076672 1.841687 0.000000  F 1.919242 -0.119166 0.000000 | ***E =*** -0.29576560  ***H =*** -0.288515473297825  ***-TS =*** -0.033502805901019  ***G =*** -0.322018279198844  Se 0.134757 0.077430 0.000000  Cl -0.040195 2.212413 0.000000  Cl 2.250632 -0.255192 0.000000 |
| **F_2_S∙∙∙F^–^** | **Cl_2_S∙∙∙F^–^** |
| ***E =*** -0.64568610  ***H =*** -0.634270196570444  ***-TS =*** -0.033139857492129  ***G =*** -0.667410054062573  S 0.000000 0.000000 -0.432818  F 0.000000 -1.833610 -0.399398  F 0.000000 0.000000 1.231613  F 0.000000 1.833610 -0.399398 | ***E =*** -0.55308649  ***H =*** -0.542573742952524  ***-TS =*** -0.036945064560737  ***G =*** -0.579518807513261  S 0.003090 -0.106966 0.000000  Cl 0.080778 2.266038 0.000000  Cl 2.031494 -0.279514 0.000000  F -0.253163 -1.906969 0.000000 |
| **F_2_Se∙∙∙F^–^** | **Cl_2_Se∙∙∙F^–^** |
| ***E =*** -0.64326527  ***H =*** -0.632397819404737  ***-TS =*** -0.035166655703827  ***G =*** -0.667564475108563  Se 0.000000 0.000000 -0.450573  F 0.000000 -1.959000 -0.452873  F 0.000000 0.000000 1.356318  F 0.000000 1.959000 -0.452873 | ***E =*** -0.55172979  ***H =*** -0.541546556185637  ***-TS =*** -0.038916479231043  ***G =*** -0.580463035416680  Se 0.024376 -0.083246 0.000000  Cl 0.036987 2.382743 0.000000  Cl 2.208402 -0.284675 0.000000  F -0.322421 -2.002265 0.000000 |
| **F_2_S∙∙∙Cl^–^** | **Cl_2_S∙∙∙Cl^–^** |
| ***E =*** -0.60101206  ***H =*** -0.586565509088114  ***-TS =*** -0.034536506557815  ***G =*** -0.621102015645928  S -0.008097 0.085791 0.000000  F 0.287516 1.856154 0.000000  F 1.621650 -0.193941 0.000000  Cl -0.419424 -2.233124 0.000000 | ***E =*** -0.50892149  ***H =*** -0.498654839117531  ***-TS =*** -0.037985124236988  ***G =*** -0.536639963354520  S 0.000000 0.000000 -0.396368  Cl 0.000000 -2.307262 -0.619519  Cl 0.000000 0.000000 1.634628  Cl 0.000000 2.307262 -0.619519 |
| **F_2_Se∙∙∙Cl^–^** | **Cl_2_Se∙∙∙Cl^–^** |
| ***E =*** -0.59796452  ***H =*** -0.587430658881753  ***-TS =*** -0.037641040877416  ***G =*** -0.625071699759169  Se 0.006720 0.101938 0.000000  F 0.228092 2.027481 0.000000  F 1.788092 -0.180664 0.000000  Cl -0.511619 -2.303215 0.000000 | ***E =*** -0.50730818  ***H =*** -0.497337572482161  ***-TS =*** -0.040131621829964  ***G =*** -0.537469194312125  Se 0.000000 0.000000 -0.373301  Cl 0.000000 -2.403834 -0.732515  Cl 0.000000 0.000000 1.822998  Cl 0.000000 2.403834 -0.732515 |

**Table S25.** Cartesian coordinates, bonding energies, *H, TS,* and *G* (in a.u. at 298 K) for all stationary points computed at ZORA-B3LYP/TZ2P in the gas phase using ADF.

| **SF_2_** | **SeCl_2_** |
| --- | --- |
| ***E =*** -0.53257131  ***H =*** -0.523788978506833  ***-TS =*** -0.029335335153773  ***G =*** -0.553124313660606  S 0.000000 0.000000 0.316144  F 0.000000 1.222552 1.363900  F 0.000000 -1.222552 1.363900 | ***E =*** -0.35877858  ***H =*** -0.351567513562911  ***-TS =*** -0.033620294282663  ***G =*** -0.385187807845575  Se 0.000000 0.000000 0.131755  Cl 0.000000 1.696399 1.498254  Cl 0.000000 -1.696399 1.498254 |
| **F_2_S∙∙∙Cl^–^** | **Cl_2_Se∙∙∙F^–^** |
| ***E =*** -0.74679875  ***H =*** -0.735690621959029  ***-TS =*** -0.035536704950241  ***G*** = -0.771227326909269  S -0.004694 0.129782 0.000000  F 0.336770 1.870885 0.000000  F 1.613007 -0.177672 0.000000  Cl -0.446183 -2.290719 0.000000 | ***E =*** -0.66319995  ***H =*** -0.653000820768184  ***-TS =*** -0.038983594172407  ***G =*** -0.691984414940590  Se 0.022529 -0.089042 0.000000  Cl 0.010036 2.430742 0.000000  Cl 2.245235 -0.324597 0.000000  F -0.315849 -1.989689 0.000000 |

**Table S26.** Cartesian coordinates, bonding energies, *H, TS,* and *G* (in a.u. at 298 K) for all stationary points computed at ZORA-M06/TZ2P in the gas phase using ADF.

| **SF_2_** | **SeCl_2_** |
| --- | --- |
| ***E*** *= -*0.58150533  ***H =*** -0.572548373549198  ***-TS =*** -0.029236395101058  ***G =*** -0.601784768650256  S 0.000000 0.000000 0.320642  F 0.000000 1.203288 1.359732  F 0.000000 -1.203288 1.359732 | ***E =*** -0.40681306  ***H =*** -0.399531878638012  ***-TS =*** -0.033478859751333  ***G =*** -0.433010738389345  Se 0.000000 0.000000 0.130580  Cl 0.000000 1.664328 1.499340  Cl 0.000000 -1.664328 1.499340 |
| **F_2_S∙∙∙Cl^–^** | **Cl_2_Se∙∙∙F^–^** |
| ***E =*** -0.80326031  ***H =*** -0.792039830839742  ***-TS*** = -0.035398967842932  ***G =*** -0.827438798682674  S 0.146732 -0.437245 0.000000  F 1.890619 -0.517477 0.000000  F 0.242265 1.183086 0.000000  Cl -2.279616 -0.228364 0.000000 | ***E =*** -0.72576138  ***H =*** -0.715511670967266  ***-TS = -***0.038665359468609  ***G =*** -0.754177030435875  Se 0.004663 -0.091549 0.000000  Cl 0.066152 2.408845 0.000000  Cl 2.199134 -0.299961 0.000000  F -0.264190 -1.980264 0.000000 |

**Table S27.** Cartesian coordinates, bonding energies, *H, TS,* and *G* (in a.u. at 298 K) for all stationary points computed at ZORA-M06-2X/TZ2P in the gas phase using ADF.

| **SF_2_** | **SeCl_2_** |
| --- | --- |
| ***E =*** -0.77474743  ***H = -***0.765699534228579  ***-TS =*** -0.029217201558719  ***G =*** -0.794916735787298  S 0.000000 0.000000 0.317422  F 0.000000 1.199789 1.362544  F 0.000000 -1.199789 1.362544 | ***E =*** -0.53721338  ***H =*** -0.529906630945633  ***-TS =*** -0.033423600315376  ***G =*** -0.563330231261009  Se 0.000000 0.000000 0.128552  Cl 0.000000 1.654678 1.501689  Cl 0.000000 -1.654678 1.501689 |
| **F_2_S∙∙∙Cl^–^** | **Cl_2_Se∙∙∙F^–^** |
| ***E =*** -1.04144939  ***H =*** -1.030023629201243  ***-TS =*** -0.035089058951826  ***G*** = -1.065112688153069  S -0.027119 0.135223 0.000000  F 0.356555 1.821743 0.000000  F 1.562473 -0.187561 0.000000  Cl -0.408330 -2.262508 0.000000 | ***E =*** -0.92143565  ***H =*** -0.911069723602177  ***-TS*** = -0.038192518683670  ***G =*** -0.949262242285847  Se -0.025617 -0.094294 0.000000  Cl 0.136531 2.393224 0.000000  Cl 2.154487 -0.282527 0.000000  F -0.190551 -1.974975 0.000000 |
